# Maternal Obesity and Outcomes During Pregnancy and the Puerperium: A Single-Center Retrospective Observational Study

**DOI:** 10.3390/medsci14060688

**Published:** 2026-10-20

**Authors:** Denise Souza Ribeiro, Luiz Felipe Azevedo Marques, Fernando Augusto Lima Marson

**Affiliations:** 1 Postgraduate Program in Health Sciences, Postgraduate Program in Health Data Science, Laboratory of Molecular Biology and Genetics, São Francisco University (Universidade São Francisco, USF), Bragança Paulista 12916-900, SP, Brazil; ribeiro.denise@mail.usf.edu.br (D.S.R.); luiz.azevedo@mail.usf.edu.br (L.F.A.M.); 2 Postgraduate Program in Health Sciences, Postgraduate Program in Health Data Science, Laboratory of Clinical and Molecular Microbiology, São Francisco University (Universidade São Francisco, USF), Bragança Paulista 12916-900, SP, Brazil; 3 Postgraduate Program in Health Sciences, Postgraduate Program in Health Data Science, LunGuardian Research Group–Epidemiology of Respiratory and Infectious Diseases, São Francisco University (Universidade São Francisco, USF), Bragança Paulista 12916-900, SP, Brazil

**Keywords:** body mass index, cesarean delivery, gestational diabetes mellitus, hypertensive disorders of pregnancy, large for gestational age, maternal obesity, multiple imputation, perinatal outcomes

## Abstract

**Background:** Maternal obesity is associated with pregnancy complications and adverse perinatal outcomes, although observational associations may be influenced by confounding, exposure ascertainment, and missing data. This study investigated associations between recorded maternal body mass index (BMI) and maternal clinical conditions, obstetric complications, delivery characteristics, and perinatal outcomes in a referral center. **Methods:** A retrospective study was conducted using medical records of pregnant women at a referral center. Recorded maternal BMI categories were classified according to the World Health Organization. Database standardization harmonized equivalent diagnostic terms, preserved maternal clinical conditions and obstetric complications as separate domains, and grouped uncommon conditions for descriptive and exploratory analyses. Missing non-BMI variables underwent multiple imputation across 50 completed datasets; maternal BMI was not imputed. Twin pregnancies were excluded, and women with and without recorded BMI were compared before and after imputation. All singleton pregnancies with recorded BMI were included descriptively, whereas association analyses excluded women with underweight because of their small number and absence of recorded primary-outcome events. The primary composite perinatal outcome comprised neonatal intensive care unit admission, recorded fetal death or stillbirth, or a 5-min Apgar score <7 among liveborn infants. Logistic regression models compared overweight and obesity with normal weight, adjusting for maternal age and parity. Selected outcomes underwent additional adjustment for smoking, chronic hypertension, and pre-existing diabetes mellitus. Complete-case analyses assessed the sensitivity of the core findings. **Results**: The source database comprised 1012 records. After excluding 13 twin pregnancies, 999 singleton pregnancies remained; BMI was recorded for 787 women and missing for 212 (21.2%). Among women with recorded BMI, 27 (3.4%) had underweight, 282 (35.8%) normal weight, 210 (26.7%) overweight, and 268 (34.1%) obesity. Association analyses included 760 women. Compared with normal weight, obesity was associated with maternal clinical conditions (adjusted odds ratio, 3.04; 95% confidence interval, 2.06–4.49), chronic hypertension (8.96; 4.14–19.38), obstetric complications (4.12; 2.83–5.99), hypertensive disorders of pregnancy (6.12; 3.60–10.40), gestational diabetes mellitus (2.43; 1.48–3.97), cesarean delivery (3.18; 2.12–4.75), and large-for-gestational-age birth (5.99; 2.74–13.13). Overweight was also associated with obstetric complications, gestational diabetes mellitus, cesarean delivery, and large-for-gestational-age birth. The associations between obesity and overall obstetric complications, hypertensive disorders of pregnancy, and large-for-gestational-age birth remained evident in expanded models. No statistically significant association was observed with the primary composite outcome for overweight (0.58; 0.21–1.57) or obesity (1.16; 0.54–2.53), and complete-case analyses were similarly inconclusive. Core models did not identify statistically significant associations with preterm birth, small-for-gestational-age birth, or low birth weight. The exploratory association with perineal tears was attenuated after adjustment. Women without recorded BMI had fewer recorded maternal clinical conditions, obstetric complications, and cesarean deliveries, but more composite perinatal events; these differences persisted after imputation. **Conclusions:** Higher recorded maternal BMI was associated with maternal morbidity, cesarean delivery, and large-for-gestational-age birth after adjustment for age and parity. The composite perinatal findings remained inconclusive because of limited event frequency and imprecise estimates, rather than demonstrating absence of increased risk. Differences according to BMI availability indicate potential selection bias, while uncertain exposure timing and residual confounding further constrain interpretation. The findings support individualized preconception and antenatal assessment and standardized clinical documentation, without establishing causal relationships or the effectiveness of a specific intervention.

## 1. Introduction

Obesity is characterized by excess body fat that can impair health and is a major contributor to noncommunicable chronic diseases, such as type 2 diabetes mellitus, cardiovascular disease, and systemic arterial hypertension [1,2]. Obesity affects all age groups, including women of reproductive age, and has become an important public health concern during pregnancy. Maternal obesity is associated with adverse maternal and fetal outcomes, including pregnancy complications, preterm birth, fetal macrosomia, and cesarean delivery [3,4,5].

During pregnancy, women experience a physiological increase in insulin resistance. In women with obesity, pre-existing insulin resistance may increase the metabolic demands on pancreatic function and contribute to the development of gestational diabetes mellitus [6,7]. Consequently, gestational diabetes mellitus is more frequent among women with obesity than among those with normal weight [8]. Maternal obesity is also associated with hypertensive disorders of pregnancy, which may involve metabolic, inflammatory, and vascular alterations [3,8,9]. These complications can contribute to maternal and fetal morbidity and may require medically indicated preterm delivery, whereas associations with spontaneous preterm birth vary across populations and maternal characteristics [3,10].

Women with obesity may experience difficulties in labor progression and a greater likelihood of cesarean delivery. Factors investigated in this context include labor arrest, cephalopelvic disproportion, induction failure, and fetal macrosomia [5,11]. However, these associations may also reflect differences in maternal age, parity, previous cesarean delivery, comorbidities, and obstetric management. Obesity can additionally complicate anesthetic and surgical care, with concerns including increased intraoperative blood loss, venous thromboembolism, technical difficulties, and wound complications [5,9,12]. These considerations emphasize the importance of evaluating delivery outcomes within their clinical context rather than attributing them exclusively to maternal body mass index (BMI).

Fetal macrosomia is an important outcome associated with maternal obesity and gestational diabetes mellitus [13,14]. Maternal hyperglycemia increases fetal glucose exposure, stimulating fetal insulin secretion and promoting growth and fat deposition [6,7]. Alterations in adipokine signaling, including leptin, have also been investigated in relation to the metabolic disturbances associated with gestational diabetes mellitus [15]. Nevertheless, fetal growth is influenced by several maternal and pregnancy-related factors. Macrosomia, defined by an absolute birth-weight threshold, should therefore be distinguished from large-for-gestational-age birth, which considers birth weight relative to gestational age [14,16].

The increasing prevalence of obesity among women of reproductive age has implications for pregnancy, delivery, and maternal and newborn health [3]. Prepregnancy obesity and excessive gestational weight gain are related but distinct exposures, both of which have been associated with adverse pregnancy outcomes [3,17,18]. Although BMI is widely used to characterize maternal weight status, its interpretation depends on the timing and source of the anthropometric measurements. Measurements obtained after pregnancy-related weight gain may not represent prepregnancy weight status. Similarly, differences in maternal characteristics and the availability of BMI information can influence associations observed in retrospective medical-record studies.

For this reason, the objective of this study was to retrospectively analyze pregnant women’s medical records in a referral center, describe their sociodemographic and clinical characteristics, and investigate associations between recorded maternal BMI and maternal clinical conditions, obstetric complications, delivery characteristics, and perinatal outcomes. The primary outcome was a composite of neonatal intensive care unit admission, recorded death, or a low Apgar score at five minutes. Secondary analyses examined individual maternal, obstetric, delivery, and newborn markers, with adjustment for clinically relevant characteristics where feasible. Women with and without recorded BMI were also compared to assess differences associated with exposure availability.

## 2. Materials and Methods

### 2.1. Study Design and Ethical Approval

We conducted a single-center retrospective observational study using existing records of women admitted for delivery at the University Hospital from January 2019 to January 2020. The available cohort represented a convenience sample of hospital activity; the sample size was determined by the records available for review, and no prospective sample-size calculation was performed. The analyses assess associations between recorded maternal BMI categories and maternal, obstetric, and perinatal characteristics.

Data collection followed authorization from the hospital administration and approval by the Research Ethics Committee of the São Francisco University (approval number 4.096.853). The ethics committee waived informed consent because the study used existing medical records without direct participant contact. Data were handled confidentially and the analytical files used record identifiers rather than participant names.

### 2.2. Study Population and Analytical Populations

The source database contained 1012 records. All records were retained during missing-data assessment and multiple imputation. Thirteen records identified as twin pregnancies were subsequently excluded, leaving 999 singleton pregnancies. Among these, maternal BMI was available for 787 women and unavailable for 212. Records with missing BMI were retained for the assessment of BMI availability but were excluded from analyses requiring an observed BMI category. No participant was excluded solely because another study variable required imputation.

The descriptive analyses by BMI included all 787 women with recorded BMI: 27 with underweight, 282 with normal weight, 210 with overweight, and 268 with obesity. The BMI-based association analyses included the 760 women in the normal-weight, overweight, and obesity groups. The underweight group remained visible in descriptive tables but was excluded from the association analyses because the subgroup contains only 27 women and no recorded or imputed event of the primary composite outcome. This revision-stage restriction defines the population to which the association estimates apply; it does not establish that underweight is protective. Its small size would also produce imprecise comparisons, and the absence of primary events creates separation in conventional logistic regression.

### 2.3. Data Source and Database Organization

Data were extracted using the authors’ structured collection forms, provided as Supplementary Material S1. Sources included prenatal cards, hospital admission documents, medical-record identification forms, and the clinical records available for the index pregnancy and delivery. The database was organized in Microsoft Excel (version 16.113.2; Microsoft Corporation, Redmond, WA, USA), with one row per participant and separate fields for BMI, sociodemographic information, clinical conditions, obstetric complications, reproductive history, delivery characteristics, and perinatal outcomes. Original record positions were retained to permit verification of corrections and imputed cells.

Maternal clinical conditions and obstetric complications were retained as separate domains. Label harmonization addressed spelling, capitalization, accents, abbreviations, synonymous terms, units, and category boundaries. Clinically related diagnoses were organized into broader descriptive groups without treating them as interchangeable diseases. Recorded combinations of coexisting diagnoses were retained as single categories for descriptive reporting within each categorical field. Disease-specific binary indicators were not mutually exclusive: a woman with more than one documented condition could contribute once to each relevant indicator. However, she contributed only once to the “any maternal clinical condition” indicator, regardless of the number of clinical diagnoses. The same principle applied separately to the “any obstetric complication” indicator. Thus, disease-specific frequencies may overlap and must not be summed to obtain the number of affected women. The mapping between recorded categories and the binary indicators used in the analyses is provided in Table S1. The mapping of recorded diagnostic combinations to the binary indicators used in the analyses is detailed in Table S1a. A participant could contribute once to each applicable condition-specific or grouped indicator but only once to the corresponding overall clinical or obstetric indicator.

Hypertensive disorders of pregnancy were defined from the recorded obstetric categories: “hypertensive disorder of pregnancy,” “hypertensive disorder of pregnancy; preeclampsia,” “hypertensive disorder of pregnancy; HELLP (hemolysis, elevated liver enzymes, and low platelet count) syndrome,” “eclampsia,” and “preeclampsia.” Each woman with any of these entries was classified as having the outcome and counted once. Chronic hypertension alone was excluded from this outcome and retained as a separate maternal clinical indicator. Women with chronic hypertension could nevertheless have a positive obstetric outcome if a pregnancy-related hypertensive complication was also recorded. Superimposed preeclampsia was not separately identifiable in the available categories and was not inferred solely from the coexistence of chronic hypertension and preeclampsia indicators. The nonspecific “hypertensive disorder of pregnancy” category was retained as recorded and was not retrospectively assigned a more specific diagnosis.

Blank cells and entries recorded as ignored or not informed were treated as missing. Explicit entries of no, absent, or not applicable were retained as observed information. Missing values were not generally recoded as absence of disease.

The cleaned data were checked for permitted values, mutually exclusive categories, consistency between gravidity, parity and previous pregnancy losses, agreement between delivery route and the applicability of perineal variables, and consistency of derived outcomes with their components. Observed values were preserved except for the documented author-adjudicated corrections. Imputation was restricted to genuinely missing fields, with structural restrictions described below.

#### 2.3.1. Maternal BMI and Anthropometric Reference

Maternal BMI was the exposure defining the study groups and was calculated as weight in kilograms divided by height in meters squared when the required anthropometric information was available in the source records. The preferred weight reference is reported pre-pregnancy weight; if unavailable, a measurement obtained by the 13th gestational week is the preferred early-pregnancy substitute, consistent with Brazilian Ministry of Health guidance [19]. Weight at delivery or during later pregnancy cannot be assumed to represent pre-pregnancy weight because gestational weight gain affects the resulting BMI.

Anthropometric information was obtained from the first prenatal assessment documented in the reviewed records. A first appointment did not establish that the measurement occurred by 13 weeks. The analytical database retained BMI categories but did not contain raw weight and height values, record-level gestational age at anthropometric assessment, or a consistently verified distinction between measured and self-reported measurements. The recorded classifications were therefore used as the exposure, with uncertainty about timing and provenance acknowledged as a potential source of exposure misclassification. A recorded first-visit BMI was not universally classified as verified pre-pregnancy BMI.

BMI categories were underweight (<18.5 kg/m^2^), normal weight (18.5 to <25.0 kg/m^2^), overweight (25 to <30.0 kg/m^2^), and obesity (≥30 kg/m^2^) [1,2]. The obesity cutoff includes exactly 30.0 kg/m^2^. BMI and BMI-group membership were not imputed or reconstructed through imputation of weight or height. Missing BMI remained missing in every completed dataset. Continuous BMI, obesity classes, gestational weight gain, and gestational-age-specific maternal nutritional classifications were not derived from category-only data.

#### 2.3.2. Sociodemographic Maternal and Reproductive Markers

Sociodemographic and lifestyle markers comprised recorded race, maternal age, marital status, educational attainment, smoking, alcohol consumption, and illicit drug use. Race labels were standardized as White, Mixed race, and Black for the categories represented in the cleaned database; Mixed race corresponds to the original Brazilian parda category. Age was recorded in seven bands: 15–19, 20–24, 25–29, 30–34, 35–39, 40–44, and 45–49 years. Educational categories were retained at their recorded level for descriptive reporting. Further collapses for association models followed an explicit frequency and clinical rationale rather than observed *p*-values.

Maternal clinical markers included the overall presence of a recorded clinical condition and separate indicators for chronic hypertension, hypothyroidism, urinary tract infection, asthma, pre-existing diabetes, diabetes type, syphilis, and depression. Less frequent diagnoses were retained under the following descriptive headings: noninfectious renal and urological conditions; other cardiovascular conditions; hematological conditions and thrombophilias; autoimmune conditions; gastrointestinal and hepatobiliary conditions and hernias; neurological conditions and other mental disorders; gynecological conditions, neoplasms, and masses of undetermined nature; other maternal infections; and other specified clinical conditions and medical history. These headings organize heterogeneous diagnoses and are not interpreted as single disease entities.

Reproductive and delivery markers comprised gravidity, parity, previous pregnancy losses, delivery route, episiotomy, recorded surgery/labor duration, and perineal tear. Gravidity was coded as one, two, or at least three pregnancies; parity as one, two, or at least three deliveries. These categories describe the database coding and do not redefine the broader clinical meanings of multigravida or multipara. Previous pregnancy losses were retained as no recorded loss, one, two, or three losses; a spontaneous or induced mechanism was not inferred.

Delivery route distinguished cesarean and vaginal delivery in the cleaned analytical database. Episiotomy was a separate procedure marker and was not coded as a spontaneous perineal tear. Tear categories represented no tear, first-degree tear, and second-degree tear in the available records. The original collection form allowed additional degrees, but unobserved categories were not added as actual cases. Perineal variables were structurally not applicable to cesarean deliveries. Recorded duration categories were <30, 30–60, 60–120, or >120 min. Because this field combines surgery and labor and does not identify a verified labor stage, it was described as recorded surgery/labor duration rather than a validated diagnosis of prolonged labor.

#### 2.3.3. Obstetric, Fetal, and Perinatal Markers

Obstetric and fetal markers comprised the overall presence of an obstetric complication; gestational diabetes; hypertensive disorders of pregnancy and their recorded type; fetal growth abnormalities and their recorded type; intrauterine growth restriction; recorded fetal macrosomia; fetal status abnormalities and their recorded type; fetal distress; other fetal heart-rate abnormalities; amniotic-fluid abnormalities and their recorded type; polyhydramnios; oligohydramnios; rupture of membranes; fetal congenital anomalies; placental abnormalities; and other obstetric, delivery, and puerperal conditions. Rupture of membranes was retained in the obstetric domain. Congenital anomalies, including omphalocele, were classified as fetal anomalies and were not automatically considered events of the primary composite outcome.

Diagnoses were based on the classifications recorded in the clinical documents and harmonized during data cleaning. The current analytical file does not permit retrospective confirmation of all diagnoses against independent laboratory, imaging, or guideline-based criteria. The distinction between baseline maternal disease and a complication arising during pregnancy was retained where documented. The broad clinical-condition indicator is not automatically a baseline confounder because some of its constituent diagnoses may have been recorded during pregnancy.

Perinatal markers comprised gestational-age category at birth, birth-weight category, birth weight for gestational age, the numerical 5-min Apgar score and its grouped classification, neonatal intensive care unit (NICU) admission, recorded death, NICU admission or death, and the composite adverse perinatal outcome. Birth was classified as preterm (<37 weeks), term (37 weeks to 41 weeks and 6 days), or post-term (≥42 completed weeks). Birth-weight categories were mutually exclusive: <1000 g, 1000–1499 g, 1500–2499 g, 2500–3999 g, ≥4000 g. The 2500–3999 g category was not equated with appropriate weight for gestational age. The recorded categories of small, appropriate, and large for gestational age corresponded to birth weights below the 10th percentiles, between the 10th and 90th percentiles, and above the 90th percentile, respectively; the underlying growth reference and gestational-age ascertainment method could not be independently verified from the category-only analytical file.

Preterm birth was analyzed as delivery before 37 completed weeks, irrespective of the pathway leading to delivery. The available analytical fields did not reliably distinguish spontaneous preterm labor, medically indicated preterm delivery, or preterm prelabor rupture of membranes. The recorded rupture-of-membranes indicator was not sufficient to reconstruct these phenotypes.

The Apgar measurement used was the score at 5 min. Scores were grouped as low (0–3), moderately abnormal (4–6), or reassuring (7–10). A score <7 was used as one component of the primary composite. Low Apgar was not treated as a diagnosis of neonatal asphyxia, and no inference about hypoxic injury was made without corresponding clinical and biochemical evidence. Structural non-applicability was retained as a separate recorded category where appropriate.

#### 2.3.4. Primary Outcome and Secondary Comparisons

The primary outcome was a composite adverse perinatal outcome, defined as NICU admission, recorded fetal death or stillbirth, or a 5-min Apgar score < 7 among liveborn infants. Each participant contributed at most one composite event. The death field contained entries labeled “fetal death” or “stillbirth”; these labels were retained as recorded and were not interpreted as distinct biological outcomes. No death was identified as neonatal in the available analytical database, which did not document systematic neonatal mortality surveillance or a defined postnatal follow-up period. The death component therefore represents recorded fetal deaths or stillbirths rather than neonatal mortality. Apgar scores associated with these deaths were structurally not applicable, were excluded from numerical-score imputation, and remained not applicable in all 50 completed datasets. Their death status established a composite event without requiring an Apgar score. NICU admission or recorded fetal death/stillbirth was retained as an additional, narrower composite.

The primary composite does not include maternal clinical disease, obstetric complications, episiotomy, rupture of membranes, uterine atony, oligohydramnios, delivery duration, or congenital anomaly solely because the condition is adverse. Maternal, obstetric, fetal, and neonatal markers were reported in their respective domains. For incomplete composite information, a positive observed component defines an event; absence of an event requires sufficiently observed negative components. Otherwise, the pre-imputation composite remains missing and is derived after imputing its missing components. All-negative classification was not inferred solely from blank components.

Other BMI associations were secondary or exploratory. Secondary comparisons considered for the main article included cesarean delivery, overall obstetric complications, gestational diabetes, hypertensive disorders of pregnancy, preterm birth, birth-weight abnormalities, and weight for gestational age, according to the frequency and stability criteria below. Individual rare diagnoses and rare components of the composite retained descriptive visibility.

### 2.4. Statistical Analysis

#### 2.4.1. Missing Data and Multiple Imputation

Variable-level missingness was quantified before imputation. Multiple imputation was performed on the initial 1012 record dataset before excluding twins, using a custom iterative conditional donor algorithm based on classification and regression trees implemented in Python (version 3.12.14). Fifty independently seeded chains were run for 60 iterations each, with base seed 20261001 and a chain-specific increment of 100,003.

Imputation was performed on the complete source dataset before applying the singleton-only analytical restriction, thereby retaining available information during model fitting. Twin status was included as a predictor, but donor compatibility rules did not require donors and recipients to have the same plurality status. Consequently, records of twin pregnancies were eligible donors for singleton pregnancies when they satisfied the remaining compatibility restrictions, including in the joint gestational-age, birth-weight, and birth-weight-for-gestational-age block. The 13 twin-pregnancy records were subsequently excluded from descriptive and association analyses. This sequencing was an implementation choice rather than a requirement of multiple imputation, and inclusion of twin status as a predictor did not guarantee separation of donor pools.

Missing values were imputed for race, marital status, education, delivery route, episiotomy, recorded duration, perineal tear, gravidity, parity, gestational-age category at birth, birth-weight category, birth weight for gestational age, and the numerical 5-min Apgar score. Variables with no missing values were not statistically imputed. Apgar groups and the primary composite were derived from their components where their values were missing, with consistency checks against observed derived values. The BMI category was retained as an observed predictor when available; BMI missingness and twin status were also included as predictor indicators, without filling missing BMI.

BMI was represented in the tree-model predictor matrix using one-hot indicators for the observed categories and an additional “BMI not recorded” level. For records without BMI, all observed-category indicators were zero, and the missing-category indicator was one. A separate indicator of original BMI missingness was also included, together with twin status, as implemented in the algorithm. These were predictor encodings only: no BMI category was assigned to participants with missing exposure information, and BMI remained missing in every completed dataset.

At each conditional update, a tree was fitted to a bootstrap sample of observed records, with a minimum leaf size of five and a pruning parameter of 0.0001. Missing values were filled by stochastic sampling from eligible observed donors within the assigned leaf. If no compatible donor was available in that leaf, sampling used the broader pool of compatible observed donors. The predictor set included available sociodemographic, maternal, reproductive, obstetric, and perinatal markers. Redundant diagnosis fields were represented by presence indicators where appropriate. Target variables and specified dependent or derived fields were excluded from their own predictor sets to reduce deterministic feedback.

Across the 50 chains and 60 iterations, wider-pool donor selection occurred in 553 of 6000 delivery-route draws (9.22%), 44,622 of 390,000 joint gravidity–parity draws (11.44%), and 6021 of 42,000 joint neonatal-classification draws (14.34%). No wider-pool selection occurred for the remaining imputation targets, including the numerical 5-min Apgar score (0 of 36,000 draws). These diagnostics cover iterative updates before exclusion of twin pregnancies and exclude initialization and structural assignments that did not require donor sampling. Counts refer to donor draws rather than participants; a joint draw may update several missing components. Detailed frequencies are provided in Supplementary Table S2.

Gravidity and parity were imputed jointly under constraints from observed pregnancy losses. Gestational-age category, birth-weight category, and size for gestational age were imputed jointly from compatible observed combinations. Cesarean delivery imposed structural non-applicability for episiotomy and perineal tear; those fields were imputed among vaginal deliveries when applicable. Apgar donors preserved valid observed integer scores and the restrictions associated with recorded death. Logical constraints preserved observed values and admissible category combinations. No rounded or averaged participant-level dataset was created from the 50 completed datasets.

Diagnostics examined the iteration traces of imputed category proportions, observed and imputed distributions, variation between completed datasets, constraint compliance, and split-chain R-hat summaries based on the final 40 iterations. The maximum calculated R-hat was approximately 1.011; monitored constant series were interpreted separately. These diagnostics do not prove that missingness is at random or that the imputation model is correctly specified. The working assumption is that missingness in the imputed variables is explainable by the observed predictors sufficiently for the conditional imputation model. Imputation cannot remove selection arising from unobserved BMI or undocumented factors.

The exact predictor sets, target-specific exclusions, and compatibility restrictions are documented in Table S3. Gravidity and parity were updated jointly using donors with both variables observed, preserving observed recipient values and compatibility with recorded previous pregnancy losses. Gestational-age category, birth-weight category, and birth weight for gestational age were also updated jointly from observed donor combinations matching any observed recipient components. Perineal variables were assigned structural non-applicability for cesarean deliveries; donor sampling for applicable missing values was restricted to observed vaginal deliveries. Numerical Apgar values were sampled from eligible observed numeric scores. The source code and execution instructions are provided without participant-level data.

#### 2.4.2. Comparison of Records with and Without Maternal BMI

The 999 singleton records were compared according to BMI availability, with 787 records with BMI and 212 without BMI. All 61 substantive markers other than BMI were assessed. The comparison was reported before and after multiple imputation, with a separate table quantifying variable-level missingness. In the observed-data comparison, categorical percentages used the valid denominator for each marker and group; missing-data percentages used the full group denominator. Missing observations were excluded from tests of marker distributions.

Observed global comparisons used two-sided Fisher exact tests for two-category markers. Markers with more categories used Pearson chi-square tests without continuity correction when every expected cell count was at least five; otherwise, a conditional Monte Carlo test of the Pearson statistic was used, with 9999 simulated tables, fixed margins, and seed 20261001. This simulation procedure is not described as the Fisher–Freeman–Halton exact test.

After imputation, markers whose participant-level values were unchanged in all 50 completed datasets retained exactly the same test and *p*-value as the observed-data comparison. This applied to 46 markers. For the 15 markers affected by imputation or component recalculation, global Pearson statistics were combined using the D2 method with the Enders formulation and an approximate F reference distribution [20]. Individual *p*-values were not averaged. Sparse expected counts were flagged because D2 pooling does not make an unreliable underlying chi-square approximation exact. A pooled global test was not reported for the numerical Apgar score because category support varied between completed datasets; the grouped Apgar marker was tested separately.

Imputed descriptive frequencies were averaged across completed datasets. Displayed counts were rounded using the largest remainder method within each marker and BMI-availability group so that category counts sum to 787 and 212; the total column sums these displayed group counts and therefore sums to 999. Percentages were calculated before rounding, and statistical tests used unrounded individual datasets. These displayed integers are rounded estimates rather than observed patient counts. The availability comparison describes potential selection differences and does not establish equivalence or validate the missing-at-random assumption.

#### 2.4.3. Descriptive and Association Analyses by BMI

All four observed BMI groups were included in the descriptive results. Counts, percentages, valid denominators, and missingness before imputation were reported. Descriptive summaries after imputation were identified explicitly as averages over completed datasets; if displayed as integers, the same rounding method preserved each group total. Detailed uncommon maternal and obstetric diagnoses were presented as observed counts in the main article, with fuller listings and exploratory associations in Supplementary Material. The mean imputed counts were not labeled raw data.

The primary association analyses compared overweight and obesity separately with normal weight as the reference, among 760 women. The same eligibility restriction applied across the BMI-association analyses. Overall BMI effects were distinguished from the two category-specific contrasts. Crude odds ratios (ORs) and adjusted ORs (aORs) were reported with 95% confidence intervals (95% CI). Binary logistic regression models were fitted separately in each completed dataset; log-odds coefficients and their covariance estimates were pooled using Rubin’s rules, incorporating within- and between-imputation variance [21]. ORs were obtained by exponentiating pooled coefficients. The 50 datasets were not stacked as independent observations, and no model was fitted to the rounded table. The overall two-parameter BMI effect in adjusted models was assessed using a pooled multivariate Wald test (D1), whereas individual overweight and obesity contrasts used the pooled coefficients and their uncertainty. Categorical global comparisons followed the same observed-data and D2 strategy described for BMI availability, with sparse-data limitations reported explicitly.

For the main article, secondary markers were selected according to clinical relevance, event frequency, and the reliability of the resulting estimates. Secondary binary outcomes were screened for at least 20 events overall and at least five events and five nonevents in each BMI group across all completed datasets. This was a pragmatic reporting criterion adopted during revision, not a formal power calculation or a guarantee of model validity. The primary composite was retained regardless of this secondary screen. Outcomes passing the screen still required convergence and precision assessment before adjusted results were retained. Selection did not depend on statistical significance in the unadjusted analysis.

Sparse maternal and obstetric conditions remained visible as raw observed counts; their BMI comparisons were supplementary and exploratory. Categories were collapsed only when a defensible clinical interpretation supported the grouping, with the mapping specified before fitting the model. Broad heterogeneous headings were not merged into a single causal outcome merely to increase event counts. For rare markers, finite crude estimates and their intervals were reported when supported by the data; non-estimable comparisons were identified. Exploratory sparse-data tests and any continuity correction were labeled explicitly. Adjusted models were not forced for every individual rare diagnosis. For supplementary category-versus-rest crude comparisons with a zero cell, a 0.5 continuity correction to all four cells was used only when a finite exploratory log-OR was required, and this approximation was flagged.

#### 2.4.4. Adjustment for Confounding and Subgroup Analyses

Potential confounders were selected on clinical and temporal grounds rather than bivariate significance or automated stepwise selection. The core adjusted model for the primary composite included BMI group, maternal age (<35 versus ≥35 years), and parity (one versus at least two deliveries). Normal weight was the reference category, and BMI contributed two regression parameters. Age and parity contributed one parameter each, for four slope parameters in the core model. These parsimonious representations were used because age was recorded in bands, parity was categorical, and primary-outcome events were limited. Higher-dimensional or additional adjustment was not undertaken solely because a covariate had a statistically significant unadjusted association.

Model complexity was limited in relation to the number and distribution of events, including the number of dummy-variable coefficients. Models were checked for sparse cells, complete or quasi-complete separation, nonconvergence, extreme standard errors, and implausibly wide confidence intervals. Estimates that could not be obtained reliably were identified as non-estimable; adjusted analysis was not forced for every outcome. The actual covariate set and analytical denominator were reported alongside each adjusted model.

Secondary analyses used outcome-specific consideration of confounding. For common binary outcomes selected for adjustment, the core BMI, age, and parity specification was used. Chronic hypertension, pre-existing diabetes, and smoking were considered in interpreting residual confounding; broader adjustment was constrained by event information and the absence of consistently documented baseline timing for some conditions. Chronic hypertension was included as a separate adjustment covariate in the expanded model and was not a component of the hypertensive-disorders-of-pregnancy outcome. Gestational complications, preterm delivery, and delivery route were not automatically entered into every model because they may be intermediate variables. Gravidity and parity were not routinely included together. Previous cesarean history was not available as a validated dedicated marker; generic previous surgery or parity was not substituted for it, and this limitation was acknowledged when interpreting the delivery-route analysis.

Episiotomy and perineal-tear associations were restricted to vaginal deliveries. The primary tear comparison was any recorded tear versus no tear; tear degree was secondary. These analyses concern a selected delivery subgroup and were not interpreted as effects in all pregnancies. Not applicable cases were excluded from these clinical subgroup tests, even though applicability was retained in the general BMI-availability tables. Recorded duration was analyzed within delivery route where feasible; a mixed surgery/labor duration comparison remained exploratory and was not interpreted as evidence about prolonged labor.

#### 2.4.5. Sensitivity Analyses Statistical Reporting and Software

Sensitivity analyses compared the imputed BMI-association estimates with marker-specific complete-case estimates, with the denominator reported for each analysis. The primary composite was also examined alongside its individual components and the narrower NICU-or-death endpoint. Rare components were assessed descriptively and through exploratory comparisons rather than automatically fitting unstable adjusted models. For nine more frequent conditions and outcomes, sensitivity analyses replaced binary maternal age with three categories: <25, 25–34, and ≥35 years, using <25 years as the reference. BMI and parity coding remained unchanged. Models were fitted in the same 50 completed datasets and pooled using the procedures applied to the core models. Results are presented in Table S4. Participants were not selectively deleted according to their outcome to improve statistical significance.

Because BMI was not imputed, all BMI-association estimates were conditional on having a recorded BMI. The comparison of women with and without BMI assessed potential selection differences but did not correct selection bias. No claim was made that a nonsignificant availability comparison established equivalence or that imputation resolved bias related to unobserved BMI.

The primary hypothesis concerns BMI and the composite adverse perinatal outcome. Secondary and supplementary comparisons were identified as exploratory. Two-sided tests and an alpha of 0.05 were used, with effect sizes and confidence intervals emphasized over isolated *p*-values. Multiple comparisons and the revision-stage selection of the primary outcome were acknowledged. A nonsignificant result was described as no statistically significant association detected, rather than evidence of equivalence or absence of an effect. Findings were interpreted as associations, without causal claims.

Data preparation, multiple imputation, diagnostic checks, and statistical analyses were performed in Python 3.12.14 using NumPy 2.3.5, SciPy 1.17.0, pandas 2.2.3, and scikit-learn 1.8.0. The multiple-imputation algorithm was implemented in Python.

## 3. Results

### 3.1. Study Population and Missing Data

**Figure 1:**
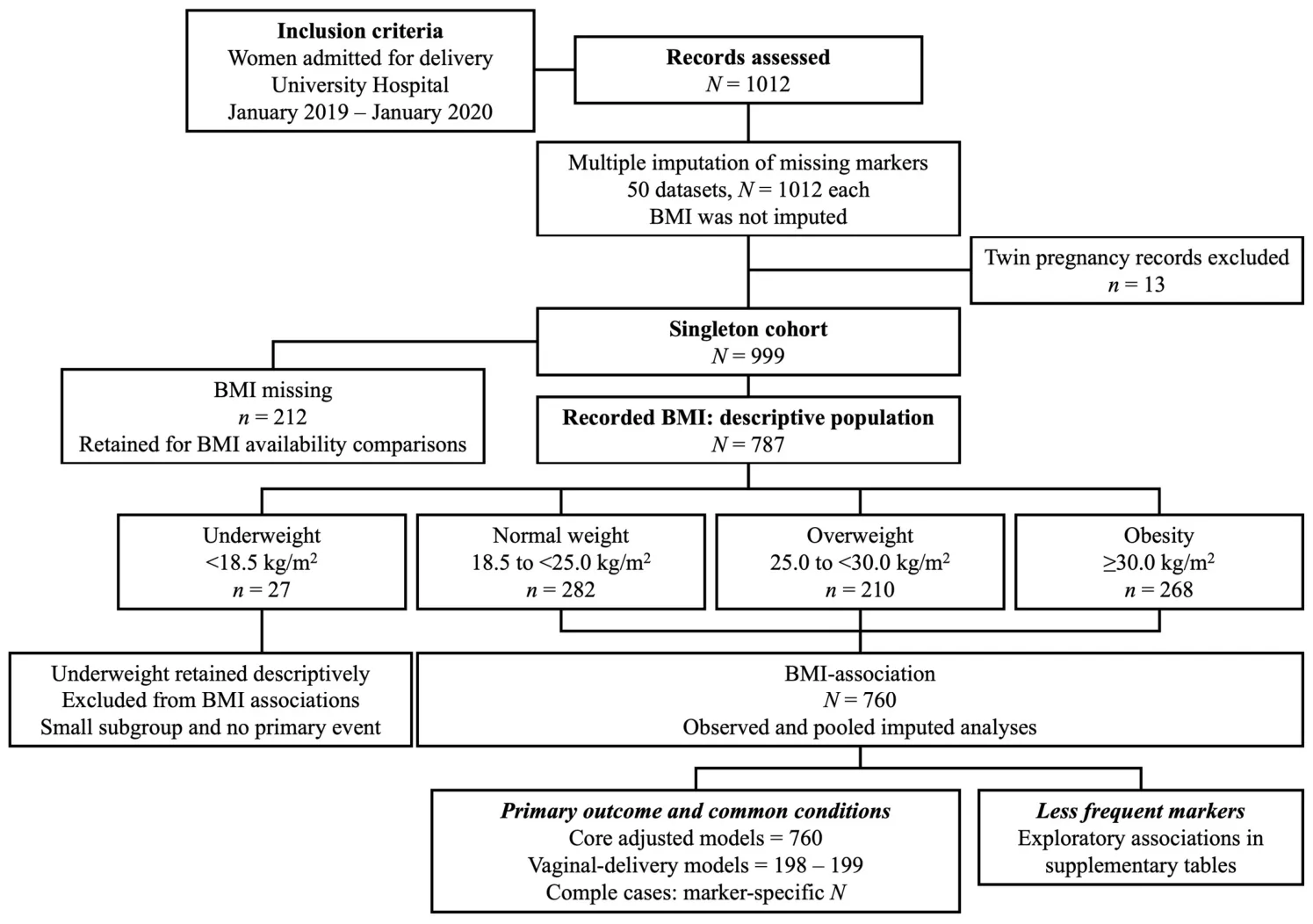
Participant inclusion and analytical populations. The source database included 1012 participants. Multiple imputation generated 50 completed datasets before exclusion of 13 records identified as twin pregnancies, leaving 999 singleton pregnancies. Maternal BMI was recorded for 787 women and missing for 212. All women with recorded BMI were included in descriptive analyses: 27 with underweight, 282 with normal weight, 210 with overweight, and 268 with obesity. BMI-based association analyses included 760 women after exclusion of the underweight group because of its small size and absence of the primary composite event. Perineal analyses were restricted to vaginal deliveries, with 198–199 women across completed datasets; complete-case sensitivity analyses used outcome-specific denominators. Women with missing BMI were included in comparisons of BMI availability but excluded from BMI-based associations. Maternal BMI was not imputed. BMI, body mass index; *n*, category frequency or analysis sample size, as specified; *N*, total group size.

The source database contained 1012 participants. After multiple imputation of eligible missing variables in the full database, 13 records identified as twin pregnancies were excluded, leaving 999 singleton pregnancies. Maternal BMI was recorded for 787 women (78.8%) and missing for 212 (21.2%). Among women with recorded BMI, 27 (3.4%) were underweight, 282 (35.8%) had normal weight, 210 (26.7%) were overweight, and 268 (34.1%) had obesity. All 787 women were included in the descriptive analyses. BMI-based association analyses included the 760 women with normal weight, overweight, or obesity; the underweight group was excluded during revision because of its small size and the absence of the primary composite event. BMI was not imputed. Figure 1 summarizes participant inclusion and the analytical populations.

Before imputation, educational attainment and gravidity each had 112 missing observations among the 999 singleton pregnancies (11.2%). Within the group with recorded BMI, these variables were missing in 82 (10.4%) and 81 (10.3%) women, respectively. Maternal age, lifestyle indicators, and the overall maternal clinical and obstetric condition indicators had no missing observations. Variable-level missingness is presented in Table S5. Observed-data and imputed descriptions by BMI category are provided in Tables S6 and S7. Unless otherwise specified, the descriptive summaries below follow the main tables after multiple imputation. For imputed variables, integer counts are rounded presentation summaries across 50 completed datasets, with percentages calculated from unrounded means; they do not represent a single completed participant database.

Women with and without recorded BMI differed in several characteristics (Tables S8 and S9). In the observed data, cesarean delivery was more frequent among women with recorded BMI (580/785; 73.9%) than among those without BMI (80/212; 37.7%; *p*-value < 0.001). Maternal clinical conditions were recorded in 230/787 (29.2%) versus 44/212 (20.8%; *p*-value = 0.015), and obstetric complications in 299/787 (38.0%) versus 45/212 (21.2%; *p*-value < 0.001). Conversely, the primary composite adverse perinatal outcome was less frequent in women with recorded BMI (34/781; 4.4%) than in those without BMI (18/209; 8.6%; *p*-value = 0.022). This difference remained after imputation (mean percentages, 4.4% versus 8.7%; pooled *p*-value = 0.012). NICU admission was also less frequent in the group with recorded BMI (10/787; 1.3%) than in the group without BMI (8/212; 3.8%; *p*-value = 0.035). These comparisons identified differences between the BMI availability groups; the BMI-based estimates below pertain to women with recorded BMI.

### 3.2. Sociodemographic Characteristics and Reproductive History

The maternal age distribution differed among the normal-weight, overweight, and obesity groups (global *p*-value < 0.001; Table 1). Women aged 15–19 years accounted for 16.7% of the normal-weight group, compared with 6.7% and 3.7% of the overweight and obesity groups. The corresponding proportions aged 35–39 years were 12.1%, 18.1%, and 16.4%. White women comprised the largest racial category in the descriptive population (rounded *n* = 558; 71.0%). There was no statistically significant global association between BMI category and race (*p*-value = 0.677), marital status (*p*-value = 0.271), or educational attainment (*p*-value = 0.646).

Smoking was reported by 56/787 women (7.1%), alcohol consumption by 24 (3.0%), and illicit drug use by nine (1.1%). These markers were not significantly associated with BMI category (*p*-value = 0.426, *p*-value = 0.135, and *p*-value = 0.190, respectively). Gravidity and parity distributions differed across BMI groups (both global *p*-values < 0.001). Three or more pregnancies were more frequent in the overweight (43.2%) and obesity (42.0%) groups than in the normal-weight group (26.8%). Similarly, three or more deliveries, including the index delivery, occurred in 39.0%, 38.8%, and 24.6%, respectively. Previous pregnancy losses were recorded in 13/787 women (1.7%); their categorical distribution was not significantly associated with BMI (*p*-value = 0.146; Table 1).

### 3.3. Maternal Clinical Conditions

Maternal clinical conditions were recorded in 230/787 women (29.2%). Their frequency increased from 55/282 (19.5%) in the normal-weight group to 59/210 (28.1%) in the overweight group and 113/268 (42.2%) in the obesity group (global *p*-value < 0.001; Table 2). Chronic hypertension was the most frequent specific clinical condition (76/787; 9.7%), followed by hypothyroidism (47; 6.0%), urinary tract infection (32; 4.1%), and asthma (29; 3.7%). Chronic hypertension was substantially more frequent in the obesity group (58/268; 21.6%) than in the normal-weight (8/282; 2.8%) or overweight groups (10/210; 4.8%; global *p*-value < 0.001). The global bivariate comparisons were not statistically significant for hypothyroidism (*p*-value = 0.059), urinary tract infection (*p*-value = 0.352), or asthma (*p*-value = 0.554).

Less frequent clinical conditions were retained as observed counts in the descriptive population of 787 women (Tables S6 and S7). Pre-existing diabetes mellitus was recorded in 14 women (1.8%), comprising two with type 1 and 12 with type 2 diabetes; syphilis was recorded in seven (0.9%), and depression in eight (1.0%). Nine women had hematological conditions or thrombophilias: six with anemia and one each with spherocytosis, sickle cell trait, or factor V Leiden-associated hereditary thrombophilia. Six had noninfectious renal or urological conditions, including two with nephrolithiasis and one each with renal atrophy, glomerulonephritis, hydronephrosis, or a recorded ureterocele–hydronephrosis combination. Six had other cardiovascular conditions: unspecified heart disease and mitral valve prolapse in two women each, and arrhythmia and hypertrophic cardiomyopathy in one each. Three had autoimmune conditions, one each with rheumatoid arthritis, an unspecified autoimmune skin disease, or systemic lupus erythematosus.

Gastrointestinal or hepatobiliary conditions and hernias were recorded in seven women, as were neurological conditions and other mental disorders, and gynecological conditions, neoplasms, or masses of undetermined nature. The neurological and mental-disorder group included two women with epilepsy and one each with an epilepsy–syncope combination, anxiety, labyrinthitis, bipolar disorder, or panic disorder. Other maternal infections were recorded in 11 women, comprising four with toxoplasmosis, three with human immunodeficiency virus (HIV) infection, and one each with hepatitis B, hepatitis C, pneumonia, or perianal condyloma acuminatum. Other specified clinical conditions and medical history were recorded in eight women: previous surgery in three, fibromyalgia in two, and emotional lability, a rickets–scoliosis combination, or allergic rhinitis in one each. The individual diagnoses and recorded combinations within these descriptive groups are detailed in Tables S6 and S7; their exploratory BMI associations are presented in Table S10. These group counts overlap with the overall maternal clinical-condition indicator and should not be added to estimate a unique total.

### 3.4. Obstetric Complications

Obstetric complications were recorded separately from maternal clinical conditions and occurred in 299/787 women (38.0%). They were more frequent in women with obesity (147/268; 54.9%) and overweight (81/210; 38.6%) than in women with normal weight (64/282; 22.7%; global *p*-value < 0.001; Table 2). The most frequent specific complications were hypertensive disorders of pregnancy (130/787; 16.5%) and gestational diabetes mellitus (125/787; 15.9%). Hypertensive disorders occurred in 20/282 (7.1%), 25/210 (11.9%), and 84/268 (31.3%) women in the normal-weight, overweight, and obesity groups, respectively (global *p*-value < 0.001). The corresponding frequencies of gestational diabetes were 28/282 (9.9%), 37/210 (17.6%), and 59/268 (22.0%; global *p*-value < 0.001).

Rare obstetric and fetal conditions remained visible as observed counts among all 787 women with recorded BMI (Tables S6 and S7). Fetal status abnormalities occurred in 21 women, including 19 with fetal distress and three with other fetal heart rate abnormalities; one woman had a combination of fetal distress and sustained fetal tachycardia. Rupture of membranes occurred in 17 women, and amniotic fluid abnormalities in 16, comprising 12 with oligohydramnios and four with polyhydramnios. Fetal growth abnormalities were recorded in ten women: seven with intrauterine growth restriction and three with a recorded diagnosis of fetal macrosomia. The latter diagnostic marker was retained separately from birth weight ≥ 4000 g.

Six women had recorded fetal congenital anomalies: unspecified malformations in two, and gastroschisis, omphalocele, Down syndrome, or a ventricular septal defect–congenital clubfoot combination in one each. Five had placental abnormalities, comprising three with placental abruption and one each with placenta accreta or placenta previa. Other obstetric, delivery, and puerperal conditions occurred in six women, including cervical insufficiency in two and uterine atony, uterine hypotonia, hemorrhage, or labor progression dystocia in one each. Recorded hypertensive subtypes and combinations, including preeclampsia, eclampsia, and HELLP syndrome, are detailed in Tables S6 and S7. The full mapping of source fields, terminology, and descriptive groupings is presented in Table S1. Recorded combinations were retained as single participant categories, and the different clinical groups were not treated as interchangeable diagnoses.

### 3.5. Delivery Characteristics and Perinatal Outcomes

Cesarean delivery predominated, with a rounded imputed summary of 580/787 (73.8%), compared with 207 (26.2%) vaginal deliveries. Cesarean delivery was more frequent in the overweight (81.0%) and obesity (82.5%) groups than in the normal-weight group (60.4%; global *p*-value < 0.001; Table 3). When recorded duration was evaluated separately by delivery route, the four-category distributions were not significantly associated with BMI for cesarean (*p*-value = 0.140) or vaginal deliveries (*p*-value = 0.483). In the exploratory binary comparison of duration > 120 min versus shorter duration among cesarean deliveries, the frequency was 5.9% in the obesity group, 1.8% in the overweight group, and 1.2% in the normal-weight group (global *p*-value = 0.015; Table S10). The original combined surgery/labor field did not establish a diagnosis of prolonged labor.

Among vaginal deliveries, the rounded imputed frequency of episiotomy was 83/207 (40.1%), with proportions of 39.6%, 52.5%, and 33.2% in the normal-weight, overweight, and obesity groups, respectively (global *p*-value = 0.185). First- and second-degree perineal tears accounted for 30 (14.6%) and 25 (12.4%) vaginal deliveries, respectively. The degree-specific distribution was not significantly associated with BMI (*p*-value = 0.183; Table 3). In the exploratory binary analysis of any tear versus no tear, proportions were 24.4%, 18.1%, and 40.8%, respectively (global *p*-value = 0.049; Table S10). These analyses were restricted to vaginal deliveries.

Most births occurred at term (rounded n = 651; 82.7%); 132 (16.8%) were preterm and four (0.5%) were post-term. Gestational-age categories did not differ significantly by maternal BMI (global *p*-value = 0.905). Birth-weight categories were associated with BMI (global *p*-value = 0.002). The rounded summary included 638 births weighing 2500–3999 g (81.1%) and 39 weighing ≥ 4000 g (5.0%). Birth weight ≥ 4000 g was more frequent in the obesity group (9.7%) than in the overweight (4.3%) or normal-weight groups (1.5%). The distribution of weight for gestational age also differed by BMI (global *p*-value < 0.001); large-for-gestational-age births accounted for 8.9% overall and occurred in 2.9%, 10.0%, and 15.3% of the normal-weight, overweight, and obesity groups, respectively (Table 3).

The rounded imputed summary of the primary composite adverse perinatal outcome was 34/787 (4.4%), with 34–36 events across completed datasets in the association population. Group-specific mean percentages were 4.7% for normal weight, 2.9% for overweight, and 5.6% for obesity (global bivariate *p*-value = 0.354). No observed or imputed composite event occurred in the underweight group. NICU admission was recorded in ten women (1.3%), death in eight (1.0%), and the narrower NICU-or-death composite in 18 (2.3%). Their global bivariate comparisons were not statistically significant (*p*-value = 0.923, *p*-value = 0.546, and *p*-value = 0.724, respectively). Death entries among women with recorded BMI were labeled fetal death. The grouped 5-min Apgar distribution was not significantly associated with BMI (*p*-value = 0.195); eight not-applicable entries were retained descriptively and excluded from that comparison. Components of the composite overlapped and were not summed as independent events (Table 3). Among the 999 singleton pregnancies, the death field contained 11 entries labeled fetal death and one labeled stillbirth. Eight of these records had available BMI, and all eight were labeled fetal death. Apgar scores were structurally not applicable for all 12 records and remained unchanged across the 50 completed datasets.

### 3.6. Adjusted Associations with Maternal BMI

After adjustment for maternal age and parity, the primary composite was not significantly associated with BMI (global *p*-value = 0.368). Relative to normal weight, the aORs were aOR = 0.58; 95% CI: 0.21–1.57; *p*-value = 0.284 for overweight and aOR = 1.16; 95% CI: 0.54–2.53; *p*-value = 0.703 for obesity. The confidence intervals were wide, reflecting the limited number of events (Table 4).

The associations with the overall maternal clinical-condition indicator persisted after adjustment: aOR = 1.62; 95% CI: 1.06–2.49; *p*-value = 0.026 for overweight and aOR = 3.04; 95% CI: 2.06–4.49; *p*-value < 0.001 for obesity (global *p*-value < 0.001). Obesity was associated with chronic hypertension (aOR = 8.96; 95% CI: 4.14–19.38; *p*-value < 0.001), whereas the overweight contrast was not significant (aOR = 1.54; 95% CI: 0.59–4.03; *p*-value = 0.374). For hypothyroidism, the global adjusted BMI test yielded *p*-value = 0.047, with a higher odds estimate for obesity (aOR = 2.50; 95% CI: 1.18–5.28; *p*-value = 0.017). Adjusted global associations remained nonsignificant for urinary tract infection (*p*-value = 0.399) and asthma (*p*-value = 0.483). These clinical-condition analyses describe contemporaneous associations rather than effects of BMI on verified pre-existing disease (Table 4).

Adjusted odds of obstetric complications were higher for overweight (aOR = 2.12; 95% CI: 1.42–3.16; *p*-value < 0.001) and obesity (aOR = 4.12; 95% CI: 2.83–5.99; *p*-value < 0.001). Both groups also had higher adjusted odds of gestational diabetes mellitus: aOR = 1.84; 95% CI: 1.08–3.14; *p*-value = 0.025 and aOR = 2.43; 95% CI: 1.48–3.97; *p*-value < 0.001, respectively. For hypertensive disorders of pregnancy, obesity remained associated with higher odds (aOR = 6.12; 95% CI: 3.60–10.40; *p*-value < 0.001), while the overweight estimate was less precise and did not reach statistical significance (aOR = 1.81; 95% CI: 0.97–3.37; *p*-value = 0.063); the global BMI test yielded *p*-value < 0.001 (Table 4).

Higher adjusted odds of cesarean delivery were observed for overweight (aOR = 2.87; 95% CI: 1.87–4.39; *p*-value < 0.001) and obesity (aOR = 3.18; 95% CI: 2.12–4.75; *p*-value < 0.001). Both groups had higher adjusted odds of large-for-gestational-age birth (aOR = 3.68; 95% CI: 1.59–8.54; *p*-value = 0.002 and aOR = 5.99; 95% CI: 2.74–13.13; *p*-value < 0.001, respectively). In contrast, adjusted global BMI associations were not significant for preterm birth (*p*-value = 0.981), birth weight < 2500 g (*p*-value = 0.143), or small-for-gestational-age birth (*p*-value = 0.478). Birth weight ≥ 4000 g had an elevated crude odds estimate for obesity (OR = 7.30; 95% CI: 2.51–21.20; *p*-value < 0.001); an adjusted model was not fitted because the normal-weight group did not meet the event-frequency criterion (Table 4).

The adjusted perineal analyses included 198–199 vaginal deliveries across completed datasets. Overweight was associated with higher odds of episiotomy in the individual contrast (aOR = 2.44; 95% CI: 1.06–5.65; *p*-value = 0.037), although the joint global BMI test was not significant (*p*-value = 0.122). Obesity was not significantly associated with episiotomy (aOR = 1.33; 95% CI: 0.56–3.15; *p*-value = 0.512). The crude obesity association with any perineal tear (OR = 2.13; 95% CI: 1.01–4.51; *p*-value = 0.047) was attenuated after adjustment (aOR = 1.73; 95% CI: 0.78–3.83; *p*-value = 0.176), and the adjusted global test was not significant (*p*-value = 0.132; Table 4).

**Figure 2:**
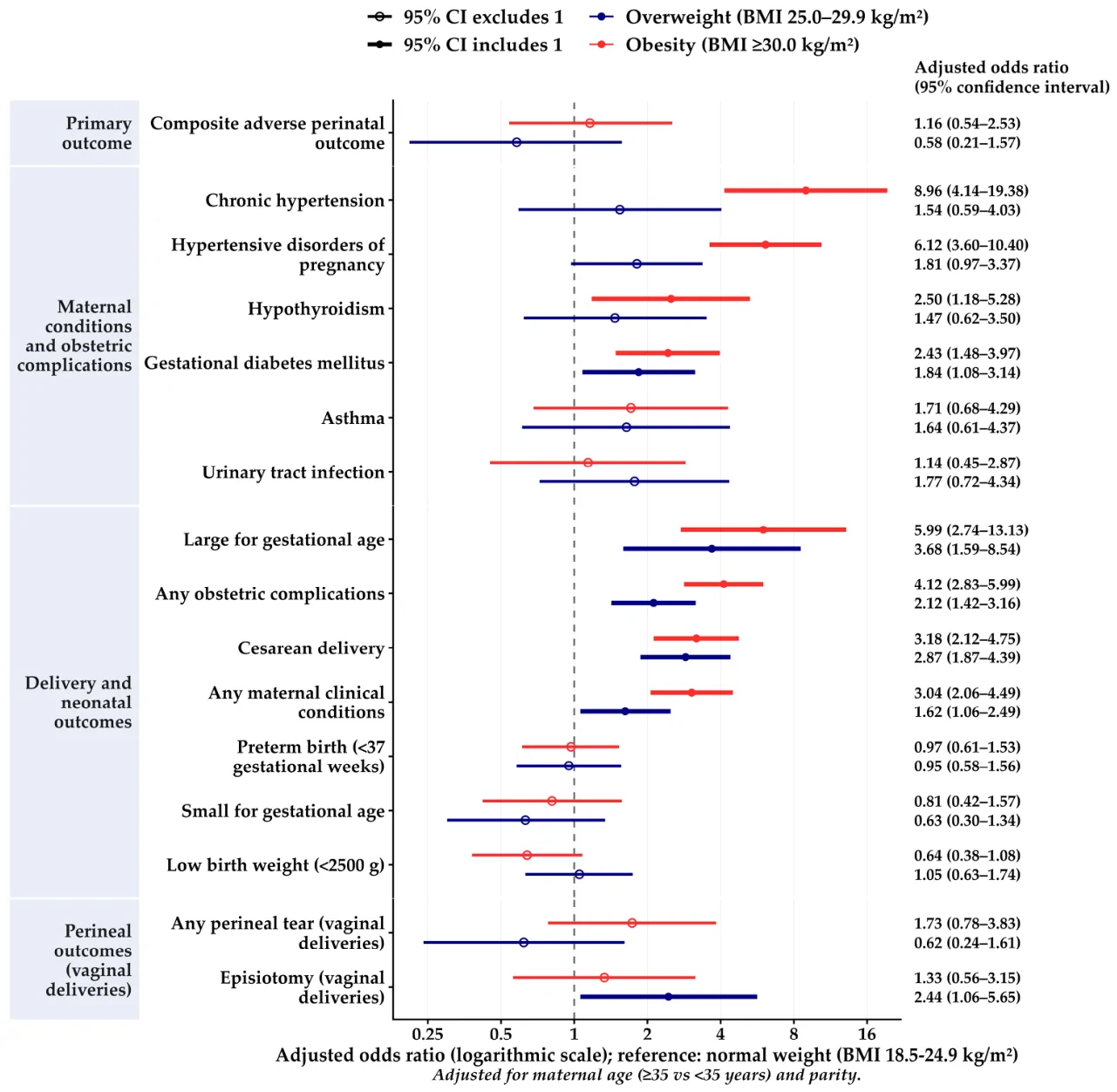
Adjusted associations of recorded maternal BMI categories with maternal clinical conditions, obstetric complications, and perinatal and delivery outcomes. Points represent adjusted odds ratios, and horizontal lines indicate 95% confidence intervals. Overweight (BMI 25.0–29.9 kg/m^2^; blue) and obesity (BMI ≥ 30.0 kg/m^2^; red) were compared with normal weight (BMI 18.5–24.9 kg/m^2^), the reference category. Each outcome was analyzed in a separate logistic regression model adjusted for maternal age (≥35 versus <35 years) and parity (≥2 versus 1 delivery, including the index delivery). Coefficient estimates and covariance matrices were pooled across 50 completed datasets using Rubin’s rules; confidence intervals incorporated within- and between-imputation uncertainty using Barnard–Rubin degrees of freedom. Maternal BMI was not imputed. The association population comprised 760 women with singleton pregnancies and recorded BMI in the normal-weight, overweight, or obesity categories. Women with underweight were retained in descriptive analyses but excluded from association analyses because of the small subgroup size and absence of primary composite events. Perineal analyses were restricted to vaginal deliveries, with 198–199 women across completed datasets. The primary composite perinatal outcome comprised NICU admission, recorded fetal death or stillbirth, or a 5-min Apgar score < 7 among liveborn infants; each participant contributed at most one composite event. “Any maternal clinical condition” and “Any obstetric complication” indicate at least one recorded condition within their respective domains. The horizontal axis uses a logarithmic scale, and the dashed vertical line marks an odds ratio of 1. Open circles indicate confidence intervals that include 1; filled circles indicate intervals that exclude 1. These symbols refer to individual contrasts and do not indicate the results of global BMI tests. Numerical estimates and confidence intervals are displayed on the right. Maternal clinical-condition analyses describe contemporaneous associations rather than verified causal effects. Secondary comparisons were exploratory and were not adjusted for multiple testing. BMI, body mass index; CI, confidence interval; NICU, neonatal intensive care unit.

Figure 2 presents the adjusted associations for the primary composite outcome and the modeled maternal, obstetric, delivery, and perinatal indicators. Several estimates indicated higher odds among women with obesity, particularly for chronic hypertension, hypertensive disorders of pregnancy, and large-for-gestational-age birth. The primary composite estimate remained imprecise and included the null. For episiotomy, the overweight contrast excluded the null, although the global BMI test was not statistically significant. The figure complements the numerical results in Table 4 by displaying the magnitude and precision of individual contrasts.

**Figure 3:**
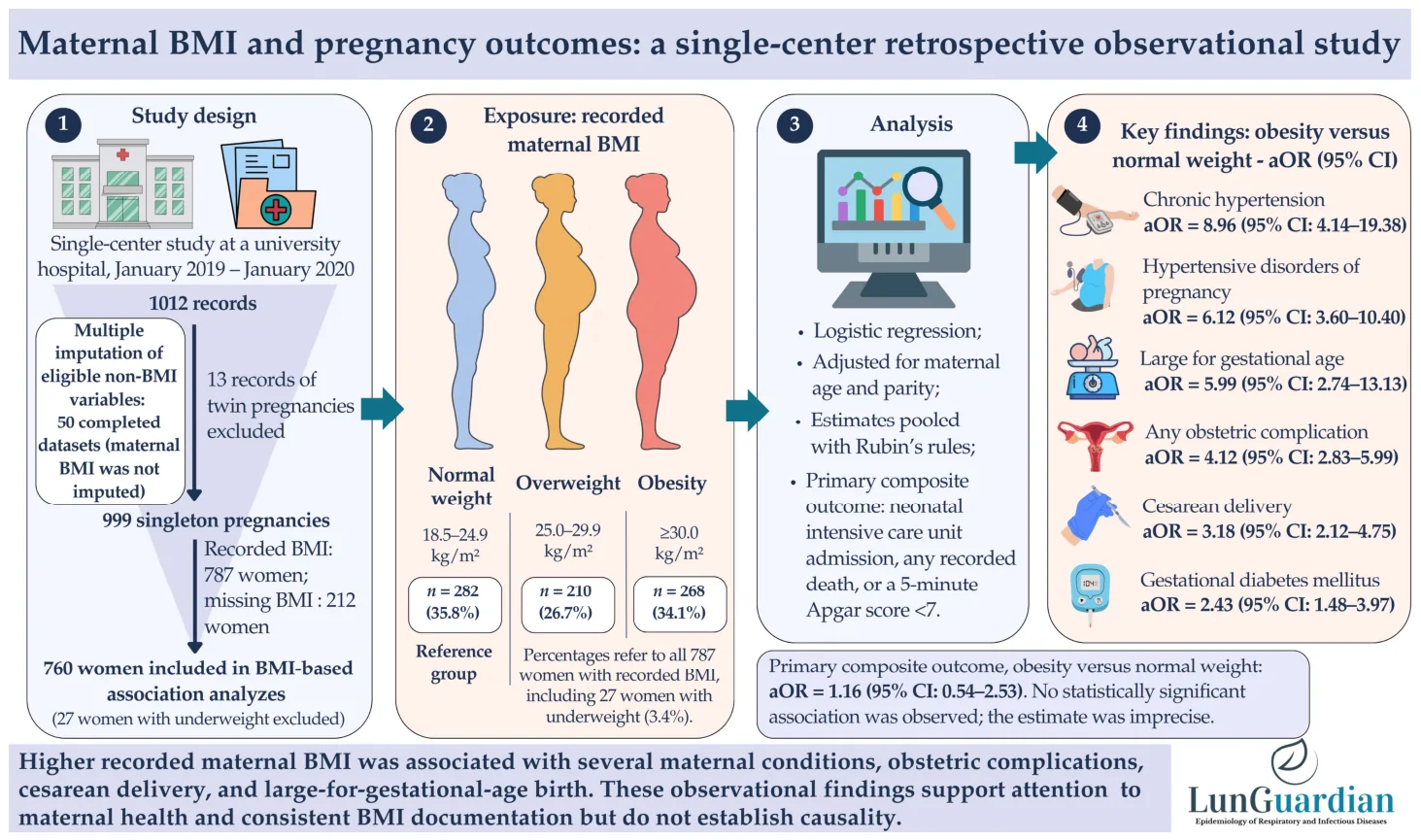
Overview of the study population, analytical approach, and selected associations with recorded maternal BMI. The source database comprised 1012 records. Multiple imputation of eligible non-BMI variables generated 50 completed datasets before exclusion of 13 records of twin pregnancies. Among 999 singleton pregnancies, 787 women had recorded BMI and were included in descriptive analyses. Percentages shown for BMI categories use these 787 women as the denominator. Association analyses included 760 women after exclusion of 27 women with underweight. Maternal BMI was not imputed. Selected estimates compare obesity with normal weight and were obtained from logistic regression models adjusted for maternal age and parity, with estimates pooled using Rubin’s rules. The primary composite perinatal outcome comprised neonatal intensive care unit admission, recorded fetal death or stillbirth, or a 5-min Apgar score < 7 among liveborn infants. Maternal clinical-condition analyses describe contemporaneous associations, and secondary outcome analyses were exploratory. The findings do not establish causality. aOR, adjusted odds ratio; BMI, body mass index; CI, confidence interval.

Figure 3 summarizes the study population, analytical approach, and selected adjusted associations. Compared with normal weight, obesity was associated with higher odds of chronic hypertension, hypertensive disorders of pregnancy, any obstetric complication, gestational diabetes mellitus, cesarean delivery, and large-for-gestational-age birth. No statistically significant association was observed with the primary composite adverse perinatal outcome, although the confidence interval was wide.

### 3.7. Sensitivity Analyses and Less Frequent Markers

Sixteen core adjusted models were estimable in all 50 completed datasets. Expanded models, which additionally included smoking, chronic hypertension, and pre-existing diabetes mellitus, were estimable for five outcomes (Table S11). Associations with obstetric complications persisted for overweight (aOR = 2.09; 95% CI: 1.40–3.13; *p*-value < 0.001) and obesity (aOR = 3.90; 95% CI: 2.65–5.73; *p*-value < 0.001). Obesity remained associated with hypertensive disorders of pregnancy (aOR = 6.82; 95% CI: 3.96–11.73; *p*-value < 0.001), and both overweight and obesity remained associated with large-for-gestational-age birth (aOR = 3.58; 95% CI: 1.54–8.33; *p*-value = 0.003 and aOR = 5.58; 95% CI: 2.51–12.39; *p*-value < 0.001, respectively). The expanded preterm-birth model did not show a significant global association with BMI (*p*-value = 0.766). For birth weight < 2500 g, the obesity estimate was lower and statistically significant in the expanded model (aOR = 0.50; 95% CI: 0.28–0.89; *p*-value = 0.018), whereas the core model estimate included the null (aOR = 0.64; 95% CI: 0.38–1.08). This finding depended on the adjustment specification and was exploratory.

Complete-case sensitivity analyses broadly preserved the direction of the main estimates, with outcome-specific denominators (Table S12). For the primary composite, 740 women and 32 events were included; the aOR was 0.58 (95% CI: 0.22–1.58) for overweight and 1.03 (95% CI: 0.46–2.30) for obesity, with global *p*-value = 0.483. For hypothyroidism, the global complete-case test was not significant (*p*-value = 0.064), although the obesity contrast remained elevated (aOR = 2.39; 95% CI: 1.12–5.10). Full coefficients and complete-case estimates are provided in Tables S11 and S12.

All nine sensitivity models using three maternal-age categories converged in all 50 completed datasets (Table S4). The direction of the BMI associations and the conclusions of both global and individual-contrast tests were unchanged, although estimates differed modestly. For gestational diabetes mellitus, the overweight estimate decreased from 1.84 (95% CI: 1.08–3.14; *p*-value = 0.025) to 1.73 (95% CI: 1.01–2.97; *p*-value = 0.046), indicating greater proximity to the null under the alternative age specification.

The expanded gestational-diabetes and cesarean-delivery models produced unstable or separated estimates, and the expanded episiotomy model had a rank-deficient design; these estimates were not pooled. Rare conditions and individual components of the primary composite did not meet the criteria for core multivariable modeling and were retained descriptively and in exploratory bivariate analyses. Table S10 reports these comparisons, including sparse-cell estimates with explicit continuity-correction flags. Numerical Apgar category support varied across imputations, preventing a pooled global comparison of the individual scores; the grouped Apgar analysis was used instead. Continuous BMI, obesity classes, gestational weight gain, and adjustment for previous cesarean delivery could not be analyzed with the available fields. Analytical feasibility and non-estimable models are documented in Table S13. All secondary and supplementary associations were exploratory and unadjusted for multiple comparisons.

## 4. Discussion

The present study identified a substantial burden of obesity among women with recorded BMI at a referral center. Obesity was associated with maternal clinical conditions, particularly chronic hypertension, obstetric complications, hypertensive disorders of pregnancy, gestational diabetes mellitus, cesarean delivery, and large-for-gestational-age birth after adjustment for maternal age and parity. The composite perinatal outcome did not show a statistically significant association with BMI, although the estimates remained imprecise. Taken together, the findings suggest that the clearest associations in this population involved maternal morbidity, delivery route, and fetal growth, while the evidence concerning less frequent perinatal events was inconclusive.

The substantial representation of women with obesity is consistent with the burden reported in Western populations and with the broader epidemiology of maternal obesity [3,11,22,23]. Lower proportions have been described in other populations, including the Chinese population examined by Chen et al. [24]. These differences should be interpreted within their clinical and methodological context. Referral patterns, access to antenatal care, population composition, BMI definitions, and the timing and source of anthropometric measurements can all influence the distribution observed in a hospital sample. Our findings therefore characterize women with available BMI in this setting rather than estimate obesity prevalence in the underlying population. The available information does not support attributing between-population differences to inherent racial characteristics or particular dietary patterns.

Maternal age and reproductive history differed across BMI categories, as has been reported in previous obstetric studies [3,11,12,25]. Women with overweight or obesity more often had a history of multiple pregnancies and deliveries than women with normal weight. These characteristics are relevant because they are also related to pregnancy complications and obstetric management. The persistence of several associations after adjustment for age and parity indicates that these measured differences did not fully account for the findings but does not establish an independent causal effect of adiposity. Categorization of age and parity, incomplete reproductive histories, and other unmeasured factors may still leave residual confounding.

Obesity has also been investigated in relation to infertility and difficulties conceiving [26,27,28]. Xu et al. described altered granulosa-cell responses to follicle-stimulating hormone and insulin signaling in women with obesity-related infertility undergoing in vitro fertilization [28]. Such observations provide a plausible biological context for reproductive differences, but their relevance to the age distribution in our population remains uncertain. Infertility, hormonal measurements, assisted conception, and the circumstances influencing pregnancy timing were not recorded reliably. Accordingly, these mechanisms cannot be invoked as demonstrated explanations for our findings, and the relationship between maternal age and BMI may also reflect social, reproductive, and health-care factors.

Chronic hypertension accounted for a prominent part of the association between obesity and maternal clinical conditions. This is compatible with the cardiometabolic burden discussed in the maternal obesity literature [3,9,25]. However, the overall clinical-condition indicator requires a broader interpretation than a strictly preconception disease history. The records included conditions that may have been recognized during pregnancy, and their timing was not consistently recoverable. The observed association consequently reflects differences in documented clinical burden. It cannot determine whether all conditions preceded conception, developed during gestation, or were identified more often because women classified as having obesity underwent more intensive assessment.

The distinction between maternal clinical conditions and obstetric complications is also important for interpreting the standardized database. Harmonizing equivalent terms and retaining clinically coherent groups improved comparability across records, while preserving separate domains avoided treating unrelated maternal, fetal, and neonatal events as a single outcome. Nevertheless, terminology standardization cannot retrospectively validate a diagnosis or establish its onset. Broad indicators summarize the presence of recorded conditions, whereas individual diagnoses address more specific clinical questions. Rare conditions and combinations of diagnoses remain useful descriptively, but associations involving heterogeneous groups should not be interpreted as evidence of a common pathophysiological process.

The findings for hypothyroidism were less consistent across analytic approaches than those for chronic hypertension. The global comparison changed with adjustment and complete-case restriction, which makes this result particularly sensitive to model specification and missing-data handling. Urinary tract infection and asthma did not show statistically significant adjusted associations with BMI. These findings illustrate why the interpretation should distinguish consistent patterns across analyses from isolated results that depend on a particular comparison. Smoking, alcohol consumption, and illicit drug use were likewise not significantly associated with BMI category, but disclosure and clinical documentation may underestimate these exposures. Incomplete reporting can limit both descriptive accuracy and control of confounding.

The associations with hypertensive disorders of pregnancy and gestational diabetes mellitus were consistent in direction with previous clinical studies and recent evidence syntheses [7,8,25,29,30]. Nassr et al. investigated adiposity-related measures in relation to these complications, and Bohiltea et al. examined obesity in the context of hypertensive pregnancy disorders [29,30]. The systematic review by Zhang et al. also identified associations of overweight and obesity with gestational diabetes mellitus, gestational hypertension, preeclampsia, and cesarean delivery [8]. Agreement in direction strengthens the clinical plausibility of our observations, but differences in exposure timing, diagnostic criteria, case mix, and adjustment strategies limit direct comparisons of association magnitude.

Insulin resistance and altered metabolic adaptation during pregnancy provide a plausible framework for the association with gestational diabetes mellitus. Pregnancy increases the demands on maternal glucose regulation, and inadequate pancreatic compensation in a setting of insulin resistance can contribute to hyperglycemia [6,7]. Adiposity-related metabolic and vascular changes may also be relevant to hypertensive pregnancy complications [3,9]. These mechanisms were not measured in our study. Their role is therefore explanatory context from the literature rather than evidence of a pathway demonstrated by our data. Moreover, clinician-recorded hypertensive subtypes were not independently adjudicated, preventing reliable inference about disease severity or the specific contribution of preeclampsia relative to other hypertensive diagnoses.

Expanded models explored whether selected associations persisted after accounting for smoking, chronic hypertension, and pre-existing diabetes mellitus in addition to age and parity. The associations with overall obstetric complications, hypertensive disorders of pregnancy, and large-for-gestational-age birth remained evident. This supports their persistence under the available alternative adjustment, while still leaving important uncertainty. Conditions included as covariates may be confounders, intermediates, or overlapping features of the outcome, depending on their timing and the clinical definition. For example, chronic hypertension may contribute to the clinical context in which a pregnancy-related hypertensive diagnosis is recorded. Without reliable temporal information and a clearly identified causal estimand, expanded adjustment should not be interpreted as isolating the direct effect of obesity.

Cesarean delivery was common in this referral setting and remained associated with overweight and obesity after adjustment for age and parity. This finding is consistent with the studies by Xiong et al., Kim et al., and Kawakita et al., as well as the recent meta-analysis by Zhang et al. [8,11,25,31]. Previous work has examined labor arrest, cephalopelvic disproportion, induction failure, and other indications that may contribute to differences in delivery route [11]. Our records did not allow these explanations to be evaluated consistently. Previous cesarean delivery was particularly important but unavailable for adjustment; parity does not adequately substitute for this history. Consequently, the adjusted association may still reflect previous obstetric management, indications for repeat surgery, or referral-related case mix.

The hospital frequency of cesarean delivery also should not be assessed against a universal numerical target without considering clinical circumstances. The World Health Organization statement discusses population-level relationships and the importance of standardized evaluation of cesarean use [32]. A referral hospital may receive a different distribution of pregnancy complications from that in the general population. Our study cannot establish whether individual cesarean deliveries were necessary or avoidable, nor whether BMI itself determined the decision. More detailed information on previous uterine surgery, presentation, induction, obstetric indications, and care pathways would be needed to understand the association more fully.

Gestational weight gain is another relevant dimension of maternal weight status. Xiong et al. evaluated prepregnancy BMI and gestational weight gain in relation to cesarean delivery, whereas Bogaerts et al. examined weight loss during pregnancy among women with obesity [31,33]. More recent synthesis by Goldstein et al. linked weight gain outside recommended ranges to a range of maternal and neonatal outcomes, with different patterns for gain below and above recommendations [17]. These findings emphasize that the clinical implications of maternal weight cannot be reduced to BMI alone. Weight change during pregnancy may influence fetal growth and obstetric outcomes while also changing the BMI classification assigned at a later antenatal visit. Because gestational weight gain was unavailable in our database, these contributions could not be distinguished. Neither our findings nor the observational comparisons cited here establish that intentional weight loss during pregnancy would improve outcomes.

The recorded duration of surgery or labor requires similarly cautious interpretation. The source field combined measurements whose clinical meanings differ according to delivery route and did not identify a specific labor stage. Although an exploratory association was observed for the longest cesarean-duration category, the overall categorical distributions within each delivery route did not show statistically significant differences. This pattern does not support a conclusion that obesity was associated with prolonged labor. It may instead reflect differences in the measurement, documentation, or duration of operative care. Without a defined starting point, ending point, and clinical process, the duration variable cannot resolve questions concerning labor progression.

Perineal outcomes were evaluated among women who delivered vaginally, and episiotomy was treated separately from spontaneous perineal injury. The exploratory association with any tear was attenuated after age and parity adjustment, while the overall adjusted BMI comparison for episiotomy was inconclusive despite a significant individual contrast. These findings should be interpreted in light of both subgroup selection and multiple comparisons. Delivery route was itself associated with BMI, so women remaining in the vaginal-delivery subgroup may differ across exposure categories in factors relevant to perineal injury. Restriction to this subgroup can therefore alter an observed association, and the results cannot represent the overall relationship between BMI and perineal outcomes in all pregnancies. The absence of recorded severe tears does not demonstrate that such injuries were prevented by episiotomy or explain institutional practice.

The association with large-for-gestational-age birth was among the more consistent fetal growth findings, remaining evident in the core and expanded models. This is compatible with previous work on maternal obesity-related traits and birth weight, including the genetic investigation by Tyrrell et al. and the clinical study by Agudelo-Espitia et al. [13,34]. The systematic review by Weir et al. also reported adverse fetal growth patterns associated with maternal obesity, with additional associations in the presence of gestational diabetes mellitus [16]. Maternal glucose regulation and nutrient availability provide plausible biological links, but their specific contribution could not be examined in our study. The persistence of the association after adjustment for pre-existing diabetes mellitus does not exclude a contribution from gestational diabetes mellitus or other metabolic pathways.

Large-for-gestational-age birth must be distinguished from macrosomia defined by an absolute birth-weight threshold and from a clinician-recorded antenatal diagnosis of fetal macrosomia. These markers refer to related but different aspects of fetal growth. The positive exploratory association with high birth weight was compatible with the large-for-gestational-age finding, but sparse events prevented comparable adjusted modeling. Maintaining these distinctions avoids overstating the consistency of separate measurements or interpreting them as interchangeable diagnoses. It also clarifies that a normal-range absolute birth weight does not, by itself, establish appropriate growth for gestational age.

The core analyses did not identify statistically significant associations with preterm birth, small-for-gestational-age birth, or low birth weight. Previous studies, including those by Liu et al. and Chen et al., have described different patterns in other populations [10,24]. Variation in population composition, BMI ascertainment, gestational weight gain, clinical care, and outcome definitions may contribute to these differences. In our expanded analysis, an inverse association with low birth weight appeared, but its dependence on adjustment specification and the exploratory nature of the comparison preclude a protective interpretation. The available evidence is insufficient to infer that obesity reduces the occurrence of small or low-birth-weight newborns.

The preterm-birth analysis combined deliveries before 37 weeks irrespective of their underlying pathway. Świercz et al. separately evaluated overall preterm delivery, spontaneous preterm birth, and preterm prelabor rupture of membranes in a study of first-trimester biochemical markers [35]. Although that study did not directly evaluate the effects of obesity, its outcome-specific approach illustrates the methodological relevance of distinguishing these phenotypes. Our database did not permit their reliable separation. Consequently, the nonsignificant association with overall preterm birth does not exclude associations with individual phenotypes, which may be obscured when clinically heterogeneous events are combined.

The composite perinatal outcome was introduced to provide a reproducible endpoint based on NICU admission, recorded death, and an abnormal Apgar score at five minutes. Its definition excluded maternal and obstetric conditions that had previously been mixed with newborn outcomes. This improved conceptual separation, but the composite still combines clinically distinct events with different severity, timing, and determinants. Its components may reflect immediate neonatal adaptation, need for specialized care, or fetal or perinatal death as documented in the records. A composite association cannot identify which mechanism is involved, and a nonsignificant composite comparison may conceal different component-specific patterns.

No statistically significant adjusted association was observed between BMI and this composite, and complete-case analyses were similarly inconclusive. Nevertheless, the small number of events and wide uncertainty around the estimates prevent exclusion of clinically important associations. The same concern applies to the individual outcomes of NICU admission and recorded death. These findings therefore do not establish neonatal safety or equivalence between BMI groups. In a contemporary nationwide Korean cohort, Kim et al. reported associations between maternal BMI and neonatal respiratory morbidity [36]. Differences in study size, exposure ascertainment, BMI classification, and outcome definitions make that evidence informative for context rather than directly comparable to our composite result.

An abnormal Apgar score at five minutes was interpreted as a marker of neonatal clinical condition, not as a diagnosis of asphyxia. Mlodawska et al. demonstrated weak correlations between Apgar scores and umbilical venous acid–base parameters in newborns with impaired clinical condition [37]. This supports the distinction between a clinical score and biochemical evidence of impaired oxygenation. Cord blood gas results and information needed to evaluate hypoxic injury were unavailable in our study. Accordingly, the analysis cannot establish acidemia, hypoxic–ischemic injury, or a causal intrapartum mechanism, and the former terminology of moderate or severe asphyxia was not retained.

The comparison between women with and without recorded BMI revealed clinically relevant differences in maternal conditions, obstetric complications, delivery route, and the composite outcome. Missing BMI therefore cannot be treated as an incidental administrative issue. Differences in care pathways, documentation, clinical complexity, or presentation may influence both exposure availability and the outcomes recorded, although the specific reasons for missing BMI could not be established. Selection into the BMI-recorded population may consequently alter the apparent associations and limit extrapolation to all women treated at the center. The direction and magnitude of this potential bias cannot be determined from the observed comparisons alone.

Multiple imputation reduced the reliance on complete-case exclusion for missing non-BMI variables and allowed uncertainty about those values to contribute to the analyses. However, it addressed a different problem from missing exposure information. BMI was retained as observed, so imputation did not restore women without an exposure classification to the BMI association analyses. The approach also relied on the assumption that missing values could be explained conditionally by the information included in the imputation models. The comparison of women with and without BMI does not validate that assumption for the other variables, and convergence of the computational procedure does not establish the absence of missing-data bias. Similar directions in several complete-case analyses provide useful context but do not eliminate concerns, because complete cases may themselves be selected.

The clinical interpretation of the adjusted results should also consider how the models were selected and what they could estimate reliably. Adjustment for age and parity addressed clinically relevant differences rather than relying exclusively on significance in bivariate comparisons. Additional adjustment was feasible only for selected outcomes, while sparse events or estimation problems prevented some expanded models from being pooled. The core models therefore remain the principal adjusted analyses for those outcomes. Odds ratios describe associations on the odds scale and should not be read as proportional increases in probability, particularly when an outcome is common. Findings that persist across available models are more persuasive than isolated significance, but none of these models overcomes uncertain exposure timing or unmeasured confounding.

These observations support the clinical relevance of coordinated preconception and antenatal assessment for women with obesity, consistent with the broader literature [18,32]. Attention to blood pressure, glucose regulation, reproductive history, fetal growth, and delivery planning may help clinicians recognize the conditions associated with higher recorded BMI. The study did not evaluate a management intervention and cannot establish that any particular strategy would prevent the observed outcomes. Its practical contribution is to identify patterns requiring careful assessment and to emphasize the value of standardized records. Consistent documentation of the source and timing of anthropometric measurements, diagnostic criteria, previous cesarean delivery, and clearly separated maternal and perinatal outcomes would improve both clinical continuity and the interpretability of subsequent research.

### Limitations

This single-center retrospective observational study is subject to selection bias, information bias, and residual confounding. The referral setting and incomplete exposure ascertainment limit generalizability. Women without recorded BMI differed clinically from those included in the association analyses, and imputation of non-BMI variables did not address missing exposure information. Women with underweight were retained descriptively but excluded from association analyses because of their small number and absence of recorded primary-outcome events. This restriction, adopted during the revision, limits the scope of inference and should not be interpreted as evidence that underweight carries no perinatal risk. No formal sample-size or power calculation was performed.

BMI timing and measurement source remain major limitations. The database did not allow verification of prepregnancy measurements, early-pregnancy measurements, or measurements obtained after substantial gestational weight gain. It was also not possible to determine consistently whether the underlying anthropometric information was measured or self-reported, and original weight and height were unavailable for recalculation. Application of adult BMI categories to measurements obtained at different stages of pregnancy may misclassify the exposure. The direction of the resulting bias is uncertain, particularly if measurement timing or reporting differs with clinical characteristics. Continuous BMI, obesity subclasses, gestational weight gain, and body composition could not be analyzed, and a restriction to verified prepregnancy or early-pregnancy measurements was not possible.

The diagnoses and outcomes reflected clinician-recorded information rather than independent retrospective adjudication. Standardization of terminology could not correct incomplete ascertainment, uncertain onset, or unverified disease severity. Documentation may differ according to the intensity of care, while lifestyle exposures may be underreported. Clinically heterogeneous rare groups and recorded combinations were retained for description but provide limited etiological specificity. Gestational age categories did not allow verification of individual dating methods, and the combined surgery/labor duration field could not define a labor stage. Perineal analyses were limited to the selected vaginal-delivery subgroup, and the absence of recorded severe tears could not be explained. Cord blood gas measurements were unavailable, and the composite combined events with different clinical meanings. In addition, neonatal mortality could not be evaluated because the available database did not document systematic neonatal death ascertainment or a defined postnatal follow-up period. The absence of neonatal-death entries must therefore not be interpreted as absence of neonatal deaths.

Multiple imputation depended on the included predictors and on a conditional missing-at-random assumption that could not be verified. Computational diagnostics assessed the behavior of the imputation procedure rather than the truth of the missing-data model. No analysis under alternative missing-not-at-random mechanisms was performed. Complete-case comparisons were informative but also susceptible to selection bias. Rounded imputed frequencies summarize multiple completed datasets and do not represent observed participant counts in a single definitive database. Sparse-data comparisons and pooled categorical tests may additionally be unstable or approximate.

Imputation preceded exclusion of twin pregnancies, and donor pools were not restricted by plurality status. Although twin status was included as a predictor, this did not prevent cross-plurality donor selection. This is particularly relevant to gestational age and birth-weight classifications, whose distributions may differ between twin and singleton pregnancies. The original diagnostics did not quantify cross-plurality donor selection, so its contribution to the imputed values cannot be determined from those summaries.

Although multivariable models addressed age and parity, these variables were simplified for modeling, and important confounders remained unavailable. Previous cesarean delivery, assisted conception, detailed socioeconomic circumstances, and the timing of maternal diagnoses could not be incorporated. Expanded models were estimable only for selected outcomes and were not available for every association. Correlated clinical conditions may function as confounders or intermediates, and adjustment for them does not identify a causal effect without further assumptions. The findings should therefore be interpreted as adjusted associations within the observed population rather than as fully controlled estimates of the consequences of prepregnancy obesity.

The primary composite and analytic restrictions were adopted during the revision rather than established in a prospectively registered analysis plan. The limited number of composite and component events resulted in imprecise estimates and constrained adjustment. Secondary and supplementary analyses involved numerous comparisons without multiplicity correction; isolated or borderline findings require an exploratory interpretation, especially when individual contrasts and global tests differ. The study cannot establish equivalence from nonsignificant results, determine the effectiveness of clinical interventions, or support causal conclusions.

## 5. Conclusions

In this retrospective observational study, maternal obesity was associated with recorded maternal clinical conditions, particularly chronic hypertension, obstetric complications, hypertensive disorders of pregnancy, gestational diabetes mellitus, cesarean delivery, and large-for-gestational-age birth after adjustment for age and parity. The composite perinatal outcome showed no statistically significant association, but the limited event frequency and imprecise estimates prevent a conclusion of no increased risk. Missing BMI, uncertain exposure timing, and residual confounding constrain the interpretation and generalizability of these findings. The results support the relevance of individualized preconception and antenatal assessment while not establishing causal relationships or the effectiveness of a specific management strategy. Standardized recording of anthropometric measurements, reproductive history, clinical diagnoses, and distinct maternal and perinatal outcomes is essential to improve clinical follow-up and strengthen future investigations.

## Figures and Tables

**Table 1:** Sociodemographic characteristics, lifestyle, and reproductive history by recorded maternal body mass index (BMI) after multiple imputation. Values are *n* (%). Maternal BMI was never imputed. Descriptive columns retain all 787 singleton pregnancies with recorded BMI. *p*-values compare only normal weight, overweight, and obesity (*N* = 760). Underweight was excluded from association analyses during revision because the subgroup was small and had no composite event. Percentages were calculated before rounding.

Markers	Underweight *N* = 27	Normal Weight *N* = 282	Overweight *N* = 210	Obesity *N* = 268	Total *N* = 787	*p*-Value
Race						0.677 ^‡^
White	20 (73.3%)	195 (69.2%)	156 (74.4%)	187 (69.9%)	558 (71.0%)	
Mixed race	6 (22.8%)	72 (25.7%)	46 (21.7%)	65 (24.1%)	189 (24.0%)	
Black	1 (3.9%)	15 (5.1%)	8 (3.9%)	16 (6.1%)	40 (5.1%)	
Maternal age (years)						<0.001 ^†^
15–19	8 (29.6%)	47 (16.7%)	14 (6.7%)	10 (3.7%)	79 (10.0%)	
20–24	10 (37.0%)	77 (27.3%)	48 (22.9%)	55 (20.5%)	190 (24.1%)	
25–29	3 (11.1%)	65 (23.0%)	51 (24.3%)	80 (29.9%)	199 (25.3%)	
30–34	6 (22.2%)	45 (16.0%)	49 (23.3%)	60 (22.4%)	160 (20.3%)	
35–39	0 (0.0%)	34 (12.1%)	38 (18.1%)	44 (16.4%)	116 (14.7%)	
40–44	0 (0.0%)	14 (5.0%)	8 (3.8%)	18 (6.7%)	40 (5.1%)	
45–49	0 (0.0%)	0 (0.0%)	2 (1.0%)	1 (0.4%)	3 (0.4%)	
Marital status						0.271 ^‡^
Married	5 (18.5%)	95 (33.5%)	78 (37.4%)	113 (42.1%)	291 (36.9%)	
Divorced	0 (0.0%)	5 (1.9%)	6 (2.9%)	7 (2.7%)	18 (2.4%)	
Single	21 (77.8%)	165 (58.7%)	108 (51.5%)	135 (50.5%)	429 (54.6%)	
Stable union	1 (3.7%)	17 (6.0%)	18 (8.3%)	13 (4.7%)	49 (6.1%)	
Educational attainment						0.646 ^‡^
Illiterate	0 (0.2%)	0 (0.1%)	1 (0.7%)	1 (0.1%)	2 (0.3%)	
Incomplete elementary school	1 (4.1%)	17 (5.9%)	19 (9.0%)	24 (9.1%)	61 (7.7%)	
Completed elementary school	6 (21.9%)	40 (14.2%)	31 (14.6%)	52 (19.4%)	129 (16.3%)	
Incomplete high school	3 (11.9%)	25 (8.9%)	21 (10.0%)	21 (7.9%)	70 (9.0%)	
Completed high school	14 (52.7%)	156 (55.5%)	112 (53.2%)	132 (49.1%)	414 (52.6%)	
Incomplete higher education	0 (0.4%)	12 (4.2%)	5 (2.3%)	11 (4.2%)	28 (3.6%)	
Completed higher education	3 (8.7%)	32 (11.2%)	21 (10.2%)	26 (9.7%)	82 (10.4%)	
Doctorate/PhD	0 (0.0%)	0 (0.0%)	0 (0.0%)	1 (0.4%)	1 (0.1%)	
Smoking	3 (11.1%)	24 (8.5%)	12 (5.7%)	17 (6.3%)	56 (7.1%)	0.426 ^#^
Alcohol consumption	3 (11.1%)	12 (4.3%)	3 (1.4%)	6 (2.2%)	24 (3.0%)	0.135 ^#^
Illicit drug use	2 (7.4%)	5 (1.8%)	1 (0.5%)	1 (0.4%)	9 (1.1%)	0.190 ^†^
Gravidity						<0.001 ^‡^
Primigravida (1 pregnancy)	19 (70.4%)	148 (52.4%)	71 (33.8%)	95 (35.6%)	333 (42.4%)	
Secundigravida (2 pregnancies)	2 (7.4%)	58 (20.7%)	48 (23.0%)	60 (22.3%)	168 (21.4%)	
Multigravida (≥3 pregnancies)	6 (22.2%)	76 (26.8%)	91 (43.2%)	113 (42.0%)	286 (36.2%)	
Parity						<0.001 ^‡^
1 delivery	19 (70.4%)	158 (56.0%)	78 (37.2%)	102 (38.2%)	357 (45.4%)	
2 deliveries	6 (22.2%)	55 (19.5%)	50 (23.8%)	62 (23.0%)	173 (21.9%)	
≥3 deliveries	2 (7.4%)	69 (24.6%)	82 (39.0%)	104 (38.8%)	257 (32.7%)	
Previous pregnancy losses						0.146 ^†^
No	27 (100.0%)	279 (98.9%)	208 (99.0%)	260 (97.0%)	774 (98.3%)	
1 pregnancy loss	0 (0.0%)	2 (0.7%)	1 (0.5%)	6 (2.2%)	9 (1.1%)	
2 pregnancy losses	0 (0.0%)	0 (0.0%)	0 (0.0%)	2 (0.7%)	2 (0.3%)	
3 pregnancy losses	0 (0.0%)	1 (0.4%)	1 (0.5%)	0 (0.0%)	2 (0.3%)	

BMI, body mass index; PhD, Doctor of Philosophy. *N*, Total group size. Fifty completed datasets were analyzed separately. Displayed counts are the mean counts rounded by the largest-remainder method within each BMI group and marker, preserving its total. The total column sums displayed group counts. Percentages use unrounded means and can differ slightly from percentages computed from the displayed integers, including a nonzero percentage with a rounded count of zero. These counts are presentation summaries, not a single completed dataset. Estimates and tests use unrounded data and account for between-imputation variation. ^#^ Pearson chi-square without continuity correction. ^†^ Conditional Monte Carlo test with fixed margins and 9999 replicates. ^‡^ D2 pooling of Pearson chi-square statistics across 50 imputations. D2 results with sparse cells are approximate. Unchanged markers retain their original test and *p*-value. BMI groups are <18.5, 18.5 to <25.0, 25.0 to <30.0, and ≥30.0 kg/m^2^. Mixed race corresponds to the recorded Brazilian parda category. Gravidity categories represent 1, 2, and ≥3 pregnancies. Parity includes the index delivery. Lifestyle and previous pregnancy losses remain exploratory comparisons.

**Table 2:** More frequent maternal clinical conditions and obstetric complications by recorded maternal body mass index (BMI) after multiple imputation. Values are *n* (%). Maternal BMI was never imputed. Descriptive columns retain all 787 singleton pregnancies with recorded BMI. *p*-values compare only normal weight, overweight, and obesity (*N* = 760). Underweight was excluded from association analyses during revision because the subgroup was small and had no composite event. Percentages were calculated before rounding.

Markers	Underweight *N* = 27	Normal Weight *N* = 282	Overweight *N* = 210	Obesity *N* = 268	Total *N* = 787	*p*-Value
Maternal clinical conditions						
Maternal clinical conditions (any)	3 (11.1%)	55 (19.5%)	59 (28.1%)	113 (42.2%)	230 (29.2%)	<0.001 ^#^
Chronic hypertension	0 (0.0%)	8 (2.8%)	10 (4.8%)	58 (21.6%)	76 (9.7%)	<0.001 ^#^
Hypothyroidism	2 (7.4%)	11 (3.9%)	11 (5.2%)	23 (8.6%)	47 (6.0%)	0.059 ^#^
Urinary tract infection	1 (3.7%)	9 (3.2%)	12 (5.7%)	10 (3.7%)	32 (4.1%)	0.352 ^#^
Asthma	0 (0.0%)	8 (2.8%)	9 (4.3%)	12 (4.5%)	29 (3.7%)	0.554 ^#^
Obstetric complications						
Obstetric complications (any)	7 (25.9%)	64 (22.7%)	81 (38.6%)	147 (54.9%)	299 (38.0%)	<0.001 ^#^
Gestational diabetes mellitus	1 (3.7%)	28 (9.9%)	37 (17.6%)	59 (22.0%)	125 (15.9%)	<0.001 ^#^
Hypertensive disorders of pregnancy	1 (3.7%)	20 (7.1%)	25 (11.9%)	84 (31.3%)	130 (16.5%)	<0.001 ^#^

BMI, body mass index. *N*, Total group size. Rows show participants with the condition. These markers contained no missing outcomes, so displayed frequencies are observed counts. The overall condition indicators can overlap with their specific diagnoses and are not additive. Specific conditions were selected for prominence using frequency, not *p*-values. Detailed rare diagnoses are retained in Tables S6 and S7; exploratory associations appear in Table S10. ^#^ Pearson chi-square without continuity correction. Unchanged markers retain their original test and *p*-value.

**Table 3:** Delivery characteristics and perinatal outcomes by recorded maternal body mass index (BMI) after multiple imputation. Values are *n* (%). Maternal BMI was never imputed. Descriptive columns retain all 787 singleton pregnancies with recorded BMI. *p*-values compare only normal weight, overweight, and obesity (*N* = 760). Underweight was excluded from association analyses during revision because the subgroup was small and had no composite event. Percentages were calculated before rounding.

Markers	Underweight *N* = 27	Normal Weight *N* = 282	Overweight *N* = 210	Obesity *N* = 268	Total *N* = 787	*p*-Value
Mode of delivery						<0.001 ^‡^
Cesarean delivery	19 (70.4%)	170 (60.4%)	170 (81.0%)	221 (82.5%)	580 (73.8%)	
Vaginal delivery	8 (29.6%)	112 (39.6%)	40 (19.0%)	47 (17.5%)	207 (26.2%)	
Recorded duration (cesarean deliveries)	*n* = 19	*n* = 170	*n* = 170	*n* = 221	*n* = 580	0.140 ^‡^
<30	0 (0.0%)	3 (1.8%)	1 (0.6%)	2 (0.9%)	6 (1.1%)	
30–60	15 (78.9%)	86 (50.3%)	89 (52.3%)	107 (48.4%)	297 (51.1%)	
60–120	4 (21.1%)	79 (46.6%)	77 (45.3%)	99 (44.8%)	259 (44.7%)	
>120	0 (0.0%)	2 (1.2%)	3 (1.8%)	13 (5.9%)	18 (3.1%)	
Recorded duration (vaginal deliveries)	*n* = 8	*n* = 112	n = 40	*n* = 47	*n* = 207	0.483 ^‡^
<30	2 (25.0%)	22 (19.2%)	7 (17.5%)	11 (24.2%)	42 (20.2%)	
30–60	6 (75.0%)	78 (69.7%)	26 (65.0%)	28 (59.3%)	138 (66.6%)	
60–120	0 (0.0%)	11 (10.1%)	7 (17.5%)	6 (12.1%)	24 (11.6%)	
>120	0 (0.0%)	1 (1.0%)	0 (0.0%)	2 (4.4%)	3 (1.5%)	
Episiotomy (vaginal deliveries)	*n* = 8	*n* = 112	*n* = 40	*n* = 47	*n* = 207	0.185 ^‡^
No	6 (75.0%)	68 (60.4%)	19 (47.5%)	31 (66.8%)	124 (59.9%)	
Yes	2 (25.0%)	44 (39.6%)	21 (52.5%)	16 (33.2%)	83 (40.1%)	
Perineal tear degree (vaginal deliveries)	*n* = 8	*n* = 112	*n* = 40	*n* = 47	*n* = 207	0.183 ^‡^
No tear	6 (75.0%)	85 (75.6%)	33 (81.8%)	28 (59.2%)	152 (73.0%)	
First degree	1 (12.5%)	15 (13.6%)	3 (8.0%)	11 (23.0%)	30 (14.6%)	
Second degree	1 (12.5%)	12 (10.8%)	4 (10.2%)	8 (17.8%)	25 (12.4%)	
Gestational age at birth						0.905 ^‡^
Preterm (<37 weeks)	6 (22.2%)	46 (16.3%)	35 (16.7%)	45 (16.9%)	132 (16.8%)	
Term (37 weeks to 41 weeks and 6 days)	21 (77.8%)	235 (83.3%)	173 (82.4%)	222 (82.7%)	651 (82.7%)	
Post-term (≥42 completed weeks)	0 (0.0%)	1 (0.4%)	2 (1.0%)	1 (0.4%)	4 (0.5%)	
Birth weight classification						0.002 ^‡^
Extremely low birth weight (<1000 g)	1 (3.7%)	4 (1.3%)	1 (0.5%)	3 (1.3%)	9 (1.1%)	
Very low birth weight (1000–1499 g)	0 (0.0%)	4 (1.5%)	4 (1.9%)	3 (1.3%)	11 (1.5%)	
Low birth weight (1500–2499 g)	6 (22.2%)	33 (11.6%)	29 (13.8%)	22 (8.0%)	90 (11.3%)	
Normal birth weight (2500–3999 g)	20 (74.1%)	237 (84.2%)	167 (79.5%)	214 (79.7%)	638 (81.1%)	
Macrosomia (≥4000 g)	0 (0.0%)	4 (1.5%)	9 (4.3%)	26 (9.7%)	39 (5.0%)	
Birth weight for gestational age						<0.001 ^‡^
Small for gestational age	3 (11.1%)	23 (8.1%)	11 (5.2%)	18 (6.6%)	55 (6.9%)	
Appropriate for gestational age	24 (88.9%)	251 (89.0%)	178 (84.8%)	209 (78.1%)	662 (84.1%)	
Large for gestational age	0 (0.0%)	8 (2.9%)	21 (10.0%)	41 (15.3%)	70 (8.9%)	
Apgar score category at 5 min						0.195 ^‡^
Low (0–3)	0 (0.0%)	0 (0.0%)	2 (1.0%)	2 (0.7%)	4 (0.5%)	
Moderately abnormal (4–6)	0 (0.0%)	8 (2.9%)	1 (0.5%)	6 (2.3%)	15 (1.9%)	
Reassuring (7–10)	27 (100.0%)	271 (96.0%)	206 (98.1%)	256 (95.5%)	760 (96.5%)	
Not applicable	0 (0.0%)	3 (1.1%)	1 (0.5%)	4 (1.5%)	8 (1.0%)	
Primary composite adverse perinatal outcome	0 (0.0%)	13 (4.7%)	6 (2.9%)	15 (5.6%)	34 (4.4%)	0.354 ^‡^
NICU admission or death	0 (0.0%)	6 (2.1%)	4 (1.9%)	8 (3.0%)	18 (2.3%)	0.724 ^†^
NICU admission	0 (0.0%)	3 (1.1%)	3 (1.4%)	4 (1.5%)	10 (1.3%)	0.923 ^†^
Recorded death	0 (0.0%)	3 (1.1%)	1 (0.5%)	4 (1.5%)	8 (1.0%)	0.546 ^†^

Fifty completed datasets were analyzed separately. Displayed counts are the mean counts rounded by the largest-remainder method within each BMI group and marker, preserving its total. The total column sums displayed group counts. Percentages use unrounded means and can differ slightly from percentages computed from the displayed integers, including a nonzero percentage with a rounded count of zero. These counts are presentation summaries, not a single completed dataset. Estimates and tests use unrounded data and account for between-imputation variation. ^†^ Conditional Monte Carlo test with fixed margins and 9999 replicates. ^‡^ D2 pooling of Pearson chi-square statistics across 50 imputations. D2 results with sparse cells are approximate. Unchanged markers retain their original test and *p*-value. BMI, body mass index; NICU, neonatal intensive care unit. *n*, category frequency or analysis sample size, as specified; *N*, total group size. The primary composite perinatal outcome comprised NICU admission, recorded fetal death or stillbirth, or a 5-min Apgar score < 7 among liveborn infants, with each pregnancy counted once. Its components overlap and must not be summed. Recorded death was fetal death among participants with BMI. Apgar categories are low (0–3), moderately abnormal (4–6), and reassuring (7–10), without equating low Apgar with asphyxia. Not-applicable Apgar entries were preserved descriptively and excluded from its global test. Birth weight 2500–3999 g does not imply appropriate weight for gestational age. Recorded fetal macrosomia (diagnosis) is separate from birth weight ≥ 4000 g. Episiotomy and tear percentages use the number of vaginal deliveries in each completed dataset, excluding cesareans. Their model population ranged from 198 to 199 women across imputations after excluding underweight. Duration was evaluated separately by delivery route because the original field combines surgery and labor; a value > 120 min is not a verified diagnosis of prolonged labor. Stratification by delivery route may introduce selection effects.

**Table 4:** Crude and adjusted associations of recorded maternal body mass index (BMI) with the primary composite and more frequent conditions and outcomes.

Condition or Outcome	BMI Contrast	Crude OR (95% CI)	aOR (95% CI)	*p*-Value Contrast *	*p*-Value BMI Global ^†^
Composite adverse perinatal outcome *n* = 760; events = 34–36	Overweight	0.60 (0.22–1.62)	0.58 (0.21–1.57)	0.284	0.368
	Obesity	1.21 (0.56–2.60)	1.16 (0.54–2.53)	0.703	
Maternal clinical conditions *n* = 760; events = 227	Overweight	1.61 (1.06–2.46)	1.62 (1.06–2.49)	0.026	<0.001
	Obesity	3.01 (2.05–4.41)	3.04 (2.06–4.49)	<0.001	
Chronic hypertension *n* = 760; events = 76	Overweight	1.71 (0.66–4.42)	1.54 (0.59–4.03)	0.374	<0.001
	Obesity	9.46 (4.42–20.27)	8.96 (4.14–19.38)	<0.001	
Hypothyroidism *n* = 760; events = 45	Overweight	1.36 (0.58–3.21)	1.47 (0.62–3.50)	0.381	0.047
	Obesity	2.31 (1.10–4.85)	2.50 (1.18–5.28)	0.017	
Urinary tract infection *n* = 760; events = 31	Overweight	1.84 (0.76–4.45)	1.77 (0.72–4.34)	0.209	0.399
	Obesity	1.18 (0.47–2.94)	1.14 (0.45–2.87)	0.787	
Asthma *n* = 760; events = 29	Overweight	1.53 (0.58–4.05)	1.64 (0.61–4.37)	0.325	0.483
	Obesity	1.61 (0.64–4.00)	1.71 (0.68–4.29)	0.257	
Obstetric complications *n* = 760; events = 292	Overweight	2.14 (1.44–3.17)	2.12 (1.42–3.16)	<0.001	<0.001
	Obesity	4.14 (2.86–5.98)	4.12 (2.83–5.99)	<0.001	
Gestational diabetes mellitus *n* = 760; events = 124	Overweight	1.94 (1.14–3.29)	1.84 (1.08–3.14)	0.025	0.002
	Obesity	2.56 (1.57–4.16)	2.43 (1.48–3.97)	<0.001	
Hypertensive disorders of pregnancy *n* = 760; events = 129	Overweight	1.77 (0.95–3.29)	1.81 (0.97–3.37)	0.063	<0.001
	Obesity	5.98 (3.54–10.10)	6.12 (3.60–10.40)	<0.001	
Cesarean delivery *n* = 760; events = 561–562	Overweight	2.78 (1.83–4.23)	2.87 (1.87–4.39)	<0.001	<0.001
	Obesity	3.08 (2.07–4.57)	3.18 (2.12–4.75)	<0.001	
Preterm birth (<37 weeks) *n* = 760; events = 125–129	Overweight	1.03 (0.63–1.66)	0.95 (0.58–1.56)	0.852	0.981
	Obesity	1.04 (0.66–1.64)	0.97 (0.61–1.53)	0.893	
Low birth weight (<2500 g) *n* = 760; events = 101–105	Overweight	1.15 (0.70–1.90)	1.05 (0.63–1.74)	0.845	0.143
	Obesity	0.70 (0.42–1.18)	0.64 (0.38–1.08)	0.097	
Birth weight ≥ 4000 g Adjusted model not fitted ^§^	Overweight	3.04 (0.92–10.01)	—^§^	—	
	Obesity	7.30 (2.51–21.20)	—^§^	—	
Small for gestational age *n* = 760; events = 50–53	Overweight	0.63 (0.30–1.32)	0.63 (0.30–1.34)	0.227	0.478
	Obesity	0.80 (0.42–1.54)	0.81 (0.42–1.57)	0.529	
Large for gestational age *n* = 760; events = 70–71	Overweight	3.75 (1.62–8.66)	3.68 (1.59–8.54)	0.002	<0.001
	Obesity	6.10 (2.80–13.28)	5.99 (2.74–13.13)	<0.001	
Episiotomy (vaginal deliveries) *n* = 198–199; events = 79–83	Overweight	1.68 (0.81–3.50)	2.44 (1.06–5.65)	0.037	0.122
	Obesity	0.76 (0.36–1.59)	1.33 (0.56–3.15)	0.512	
Any perineal tear (vaginal deliveries) *n* = 198–199; events = 52–56	Overweight	0.69 (0.27–1.74)	0.62 (0.24–1.61)	0.320	0.132
	Obesity	2.13 (1.01–4.51)	1.73 (0.78–3.83)	0.176	

aOR, adjusted odds ratio; BMI, body mass index; CI, confidence interval; OR, odds ratio. *n*, analysis sample size; event ranges indicate variation across the 50 completed datasets. Normal weight is the reference category. Core models include BMI category, age ≥ 35 versus <35 years, and parity ≥ 2 versus 1 delivery (four nonintercept coefficients). Estimates and covariance matrices were pooled using Rubin’s rules with Barnard–Rubin degrees of freedom. * Contrast *p*-values refer to adjusted logistic-regression coefficients and use pooled t-tests with Barnard–Rubin degrees of freedom. ^†^ Global BMI *p*-values refer to the joint test of the two BMI coefficients in the adjusted model and use the pooled D1 multivariate Wald test with a finite-sample correction. Adjustment variables were selected on clinical grounds, without bivariate *p*-value screening. — Adjusted estimate or *p*-value not available because the adjusted model was not fitted. ^§^ Core secondary models required ≥20 events overall and ≥5 events and ≥5 nonevents in each BMI group across all completed datasets. Birth weight ≥ 4000 g did not meet the group-level criterion, so only its exploratory crude estimate is shown. The primary composite was retained despite limited events because it is the primary endpoint of the revision. Its 34–36 events permit only a parsimonious model, and confidence intervals reflect limited precision. Maternal clinical conditions are contemporaneous association analyses and are not interpreted as effects caused by BMI. All models converged in each of the 50 completed datasets for the estimates displayed. Expanded adjustment, full coefficient tables, and complete-case sensitivity analyses appear in Tables S11 and S12. No prior-cesarean history was available for adjustment of delivery route. No multiplicity correction was applied; *p*-values outside the revision-defined primary composite are exploratory. The composite and the underweight restriction were selected during revision rather than before initial data inspection.

## Data Availability

Aggregate data supporting the findings are presented in the article and its Supplementary Materials. Participant-level medical-record data are not publicly available because of privacy and ethical restrictions. Requests for access may be directed to the corresponding author and will be considered subject to applicable institutional requirements and ethical authorization.

## Supplementary Materials

The following supporting information can be downloaded at: https://www.mdpi.com/article/10.3390/medsci14060688/s1, Supplementary Material S1. Data collection form. Table S1. Database harmonization, marker grouping, and analysis definitions used in the revised tables. Table S1a. Proposed mapping of recorded diagnostic combinations to binary analysis indicators. Table S2. Frequency of wider-pool donor selection during iterative conditional donor imputation. Table S3. Variable-specific predictor sets and compatibility restrictions used in the conditional donor imputation algorithm. Table S4. Sensitivity analyses of maternal body mass index (BMI) associations using three maternal-age categories. Table S5. Variable-level missing data before multiple imputation according to the availability of maternal body mass index (BMI) among 999 singleton pregnancies. Table S6. Full observed-data description and bivariate comparisons by recorded maternal body mass index (BMI) before multiple imputation. Table S7. Full description and bivariate comparisons by recorded maternal body mass index (BMI) after multiple imputation. Table S8. Comparison of participants with and without recorded maternal body mass index (BMI) before multiple imputation among 999 singleton pregnancies. Table S9. Comparison of participants with and without recorded maternal body mass index (BMI) after multiple imputation among 999 singleton pregnancies. Table S10. Exploratory binary associations of recorded maternal body mass index (BMI) with all applicable indicators, including less frequent diagnoses and outcome components. Table S11. Expanded multivariable sensitivity analyses and full coefficients of the core and expanded models. Table S12. Complete-case sensitivity analyses of the core age- and parity-adjusted body mass index (BMI) associations. Table S13. Analytical feasibility and departures from the expanded analysis specification.

## Abbreviations

The following abbreviations are used in this manuscript:

aOR	Adjusted odds ratio
BMI	Body mass index
CI	Confidence interval
CNPq	Conselho Nacional de Desenvolvimento Científico e Tecnológico (National Council for Scientific and Technological Development)
FAPESP	Fundação de Amparo à Pesquisa do Estado de São Paulo (São Paulo Research Foundation)
HELLP	Hemolysis, elevated liver enzymes, and low platelet count
HIV	Human immunodeficiency virus
NICU	Neonatal intensive care unit
OR	Odds ratio
PhD	Doctor of Philosophy
USF	Universidade São Francisco (São Francisco University)
