# Supplementary material for: Maternal Obesity and Outcomes During Pregnancy and the Puerperium: A Single-Center Retrospective Observational Study
Source: Med Sci (Basel). 2026 Oct 20;14(6):688. doi: 10.3390/medsci14060688 (PMC13707260; doi:10.3390/medsci14060688)
Supplement: Supplementary file 1 [file medsci-14-00688-s001.zip › medsci-4574677-supplementary.pdf]

Supplementary Material

Maternal obesity and outcomes during pregnancy and the puerperium: A single-center retrospective observational study

---

Supplementary Material 1. Data collection form.
Table S1. Database harmonization, marker grouping, and analysis definitions used in the revised tables.
Table S1a. Proposed mapping of recorded diagnostic combinations to binary analysis indicators.
Table S2. Frequency of wider-pool donor selection during iterative conditional donor imputation.
Table S3. Variable-specific predictor sets and compatibility restrictions used in the conditional donor imputation algorithm.
Table S4. Sensitivity analyses of maternal body mass index (BMI) associations using three maternal-age categories.
Table S5. Variable-level missing data before multiple imputation according to the availability of maternal body mass index (BMI) among 999 singleton pregnancies.
Table S6. Full observed-data description and bivariate comparisons by recorded maternal body mass index (BMI) before multiple imputation.
Table S7. Full description and bivariate comparisons by recorded maternal body mass index (BMI) after multiple imputation.
Table S8. Comparison of participants with and without recorded maternal body mass index (BMI) before multiple imputation among 999 singleton pregnancies.
Table S9. Comparison of participants with and without recorded maternal body mass index (BMI) after multiple imputation among 999 singleton pregnancies.
Table S10. Exploratory binary associations of recorded maternal body mass index (BMI) with all applicable indicators, including less frequent diagnoses and outcome components.
Table S11. Expanded multivariable sensitivity analyses and full coefficients of the core and expanded models.
Table S12. Complete-case sensitivity analyses of the core age- and parity-adjusted body mass index (BMI) associations.
Table S13. Analytical feasibility and departures from the expanded analysis specification.

Supplementary Material 1. Data collection form

Medical record identifier:
Date of birth (DD/MM/YYYY):

Maternal age (years):
- o 10–14
- o 15–19
- o 20–24
- o 25–29
- o 30–34
- o 35–39
- o 40–44
- o 45–49
- o Other

Race:
- o Not recorded
- o White
- o Mixed race (*Pardo*)
- o Asian (amarela in the original classification)
- o Black
- o Indigenous
- o Other

Marital status:
- o Married
- o Single
- o Widowed
- o Divorced
- o Stable union
- o Not recorded
- o Other

Educational attainment:
- o Not recorded
- o Illiterate
- o Incomplete elementary school
- o Completed elementary school
- o Incomplete high school
- o Completed high school
- o Incomplete higher education
- o Completed higher education
- o Master's degree
- o Doctorate/PhD
- o Other

Smoking status:
- o Yes
- o No
- o Not recorded

Pack-years:

Alcohol consumption:
- o Yes
- o No
- o Not recorded

Illicit drug use:
- o Yes
- o No
- o Not recorded

Type of illicit drug used:

Pre-pregnancy or early-pregnancy weight (kg):
The field label reflects the intended information collected; the timing and source of anthropometric measurements could not be verified at the record level.

Maternal height (m):

Maternal body mass index (BMI; kg/m²):
- o Underweight: <18.5 kg/m²
- o Normal weight: 18.5–24.9 kg/m²
- o Overweight: 25.0–29.9 kg/m²
- o Obesity: ≥30.0 kg/m²
- o Not recorded

Gravidity (number of pregnancies):
- o Primigravida (1 pregnancy)
- o Secundigravida (2 pregnancies)
- o Multigravida (≥3 pregnancies)
- o Not recorded

Parity (number of deliveries, including the index delivery):
- o 1 delivery
- o 2 deliveries
- o ≥3 deliveries
- o Not recorded

Recorded maternal clinical and obstetric conditions:
- o Hypertensive disorders of pregnancy
- o Hypertension
- o Gestational diabetes mellitus
- o Type 2 diabetes mellitus
- o Heart disease
- o Infections
- o Intrauterine growth restriction
- o Rupture of membranes
- o Toxoplasmosis
- o Syphilis
- o Human immunodeficiency virus infection
- o Intensive care unit admission
- o None

Recorded duration of surgery or labor:
- o <30 minutes
- o 30–60 minutes
- o 60–120 minutes
- o >120 minutes

Mode of delivery:
- o Cesarean delivery

- Vaginal delivery
- Forceps-assisted vaginal delivery

Episiotomy:
- Yes
- No

Perineal tear:
- First-degree tear
- Second-degree tear
- Third-degree tear
- No tear

Gestational age at delivery (weeks and days):

Gestational age classification at birth:
- Preterm: <37 weeks
- Term: 37 weeks to 41 weeks and 6 days
- Post-term: ≥42 completed weeks

Newborn birth weight classification:
- Macrosomia: ≥4000 g
- Normal birth weight: 2500–3999 g
- Low birth weight: 1500–2499 g
- Very low birth weight: 1000–1499 g
- Extremely low birth weight: <1000 g

Birth weight for gestational age:
- Small for gestational age (SGA)
- Appropriate for gestational age (AGA)
- Large for gestational age (LGA)

Apgar score at 5 minutes:

Neonatal intensive care unit (NICU) admission:
- Yes
- No

Reason for NICU admission:

Table S1. Database harmonization, marker grouping, and analysis definitions used in the revised tables.
The table maps the final database fields to the reporting terminology and identifies their treatment in the analyses. It does not retrospectively assign undocumented diagnoses or infer unrecorded anthropometric timing. The detailed membership and participant counts for heterogeneous groups appear in Tables S6–S7.

Source field (Portuguese)	Reporting term (English)	Coding or analysis treatment	Mapping to binary indicators
Etnia	Race	White, Mixed race (*Pardo*), and Black. Observed race labels were not used to infer ancestry. Conditional donor imputation.	Not applicable
Idade	Maternal age (years)	Seven recorded age bands. Adjusted models use ≥35 versus <35 years. Not imputed (observed/adjudicated).	Not applicable
Estado Civil	Marital status	Source categories retained without merging distinct diagnoses. Conditional donor imputation.	Not applicable
Grau de escolaridade	Educational attainment	Source categories retained without merging distinct diagnoses. Conditional donor imputation.	Not applicable
Tabagista	Smoking	Source categories retained without merging distinct diagnoses. Not imputed (observed/adjudicated).	Not applicable
Álcool	Alcohol consumption	Source categories retained without merging distinct diagnoses. Not imputed (observed/adjudicated).	Not applicable
Drogas ilicítas	Illicit drug use	Source categories retained without merging distinct diagnoses. Not imputed (observed/adjudicated).	Not applicable
Intercorrência clínica materna	Any maternal clinical conditions	Overall clinical-condition indicator. Kept separate from obstetric complications; not assumed to represent only pre-existing disease. Not imputed (observed/adjudicated).	Coded as 1 when at least one maternal clinical condition was recorded and as 0 when absence was explicitly recorded. Multiple clinical diagnoses contributed only one event to this indicator.
Hipertensão arterial	Chronic hypertension	Recorded hypertension in the maternal clinical domain, separate from pregnancy-related hypertensive disorders. Not imputed (observed/adjudicated).	Coded as 1 when chronic hypertension was documented in the corresponding clinical field, including women with other coexisting conditions. Pregnancy-related hypertensive disorders alone did not define this indicator.
Hipotireoidismo	Hypothyroidism	Source categories retained without merging distinct diagnoses. Not imputed (observed/adjudicated).	Not applicable
Infecção do trato urinário	Urinary tract infection	Source categories retained without merging distinct diagnoses. Not imputed (observed/adjudicated).	Not applicable
Asma	Asthma	Source categories retained without merging distinct diagnoses. Not imputed (observed/adjudicated).	Not applicable
Diabetes mellitus prévio	Pre-existing diabetes mellitus	Pre-existing diabetes is separate from gestational diabetes. Not imputed (observed/adjudicated).	Coded as 1 for recorded pre-existing type 1 or type 2 diabetes mellitus, including women with other coexisting conditions. Gestational diabetes alone did not define this indicator.

Source field (Portuguese)	Reporting term (English)	Coding or analysis treatment	Mapping to binary indicators
Diabetes mellitus por tipo	Type of pre-existing diabetes mellitus	Type 1 and type 2 remain separate recorded subtypes; the overall pre-existing-diabetes indicator is analyzed separately. Not imputed (observed/adjudicated).	Not applicable
Sífilis	Syphilis	Source categories retained without merging distinct diagnoses. Not imputed (observed/adjudicated).	Not applicable
Depressão	Depression	Source categories retained without merging distinct diagnoses. Not imputed (observed/adjudicated).	Not applicable
Condições renais e urológicas não infecciosas	Noninfectious renal and urological conditions	Named source categories were retained; rare heterogeneous groups remain descriptive with exploratory any-condition comparisons. Not imputed (observed/adjudicated).	Not applicable
Outras condições cardiovasculares	Other cardiovascular conditions	Named source categories were retained; rare heterogeneous groups remain descriptive with exploratory any-condition comparisons. Not imputed (observed/adjudicated).	Not applicable
Condições hematológicas e trombofilias	Hematological conditions and thrombophilias	Hematological disorders and thrombophilias share a descriptive heading only; anemia and thrombophilia are not equivalent diagnoses. Not imputed (observed/adjudicated).	Not applicable
Condições autoimunes	Autoimmune conditions	Named source categories were retained; rare heterogeneous groups remain descriptive with exploratory any-condition comparisons. Not imputed (observed/adjudicated).	Not applicable
Condições gastrointestinais, hepatobiliares e hérnias	Gastrointestinal and hepatobiliary conditions and hernias	Named source categories were retained; rare heterogeneous groups remain descriptive with exploratory any-condition comparisons. Not imputed (observed/adjudicated).	Not applicable
Condições neurológicas e outros transtornos mentais	Neurological conditions and other mental disorders	Neurological diagnoses and other mental disorders are retained individually within the descriptive group. Depression has its own indicator. Not imputed (observed/adjudicated).	Not applicable
Condições ginecológicas, neoplasias e massas de natureza não definida	Gynecological conditions, neoplasms, and masses of undetermined nature	Gynecological disease, neoplasms, and masses remain distinct recorded categories within a broad descriptive heading. Not imputed (observed/adjudicated).	Not applicable
Outras infecções maternas	Other maternal infections	Named source categories were retained; rare heterogeneous groups remain descriptive with exploratory any-condition comparisons. Not imputed (observed/adjudicated).	Not applicable
Outras condições e antecedentes clínicos especificados	Other specified clinical conditions and medical history	Other specified clinical conditions and medical history retain named diagnoses. Previous surgery is not prior-cesarean history. Not imputed (observed/adjudicated).	Not applicable
Tipo parto	Mode of delivery	Cesarean versus vaginal delivery. No dedicated prior-cesarean marker was available. Conditional donor imputation.	Not applicable
Episiotomia	Episiotomy	Episiotomy is a procedure, separate from spontaneous perineal tear. Comparative analyses use vaginal deliveries. Conditional donor imputation.	Not applicable
Tempo cirurgia/trabalho de parto	Recorded duration of surgery or labor (minutes)	Recorded categories <30, 30–60, 60–120, and >120 min. Analyze separately by delivery route; do not label the field prolonged labor. Conditional donor imputation.	Not applicable

Source field (Portuguese)	Reporting term (English)	Coding or analysis treatment	Mapping to binary indicators
Laceração parto vaginal	Perineal tear at vaginal delivery	No tear, first degree, and second degree. Cesareans are structurally not applicable. Any tear is the main binary comparison; degree is exploratory. Conditional donor imputation.	Not applicable
Número de gestações	Gravidity	1, 2, and ≥3 pregnancies. Source Excel row 425 was adjudicated as Multigesta (≥3 pregnancies). Conditional donor imputation.	Not applicable
Paridade	Parity	1, 2, and ≥3 deliveries including the index delivery; adjusted models use 1 versus ≥2. Conditional donor imputation.	Not applicable
Intercorrência obstétrica	Any obstetric complications	Overall obstetric-complication indicator. Retained independently of the maternal clinical domain. Not imputed (observed/adjudicated).	Coded as 1 when at least one obstetric complication was recorded. Multiple obstetric diagnoses contributed only one event to this indicator.
Diabetes mellitus gestacional	Gestational diabetes mellitus	Recorded gestational diabetes, distinct from pre-existing diabetes. Not imputed (observed/adjudicated).	Not applicable
Condições hipertensivas da gestação	Hypertensive disorders of pregnancy	Pregnancy-related hypertensive disorders are analyzed as an overall indicator; recorded subtypes remain descriptive. Not imputed (observed/adjudicated).	Not applicable
Condições hipertensivas da gestação - descrição/tipo	Hypertensive disorders of pregnancy: recorded type	Binary indicator coded as present for any of the following recorded obstetric categories: hypertensive disorder of pregnancy; hypertensive disorder of pregnancy with preeclampsia; hypertensive disorder of pregnancy with HELLP syndrome; eclampsia; or preeclampsia. Chronic hypertension alone was excluded and retained as a separate clinical indicator and expanded-model covariate. Superimposed preeclampsia was not separately identifiable or inferred from coexisting indicators. Each participant contributed at most one event.	Not applicable
Alterações do crescimento fetal	Fetal growth abnormalities	Source categories retained without merging distinct diagnoses. Not imputed (observed/adjudicated).	Not applicable
Alterações do crescimento fetal - descrição/tipo	Fetal growth abnormalities: recorded type	Recorded fetal growth-restriction and macrosomia diagnoses retain separate subtypes and indicators. Not imputed (observed/adjudicated).	Not applicable
Restrição do crescimento intrauterino	Intrauterine growth restriction	Source categories retained without merging distinct diagnoses. Not imputed (observed/adjudicated).	Not applicable
Macrossomia fetal	Fetal macrosomia	Source categories retained without merging distinct diagnoses. Not imputed (observed/adjudicated).	Not applicable
Alterações da condição fetal	Fetal status abnormalities	Source categories retained without merging distinct diagnoses. Not imputed (observed/adjudicated).	Not applicable
Alterações da condição fetal - descrição/tipo	Fetal status abnormalities: recorded type	Fetal distress and heart-rate abnormalities retain distinct diagnoses and recorded combinations. Not imputed (observed/adjudicated).	Not applicable
Sofrimento fetal	Fetal distress	Source categories retained without merging distinct diagnoses. Not imputed (observed/adjudicated).	Not applicable

Source field (Portuguese)	Reporting term (English)	Coding or analysis treatment	Mapping to binary indicators
Outras alterações da frequência cardíaca fetal	Other fetal heart rate abnormalities	Heart-rate abnormalities are not equated with fetal distress. Not imputed (observed/adjudicated).	Not applicable
Alterações do líquido amniótico	Amniotic fluid abnormalities	Source categories retained without merging distinct diagnoses. Not imputed (observed/adjudicated).	Not applicable
Alterações do líquido amniótico - descrição/tipo	Amniotic fluid abnormalities: recorded type	Polyhydramnios and oligohydramnios retain separate recorded types. Not imputed (observed/adjudicated).	Not applicable
Polidrâmnio	Polyhydramnios	Source categories retained without merging distinct diagnoses. Not imputed (observed/adjudicated).	Not applicable
Oligoâmnio	Oligohydramnios	Source categories retained without merging distinct diagnoses. Not imputed (observed/adjudicated).	Not applicable
Rotura de membranas	Rupture of membranes	Rupture of membranes is an obstetric marker, not a maternal antecedent or a spontaneous perineal tear. Not imputed (observed/adjudicated).	Not applicable
Anomalias congênitas fetais	Fetal congenital anomalies	Congenital fetal anomalies include omphalocele and other named anomalies. A diagnosis alone does not define the primary composite. Not imputed (observed/adjudicated).	Not applicable
Alterações placentárias	Placental abnormalities	Placental abruption, placenta previa, and placenta accreta remain distinct diagnoses within the placental group. Not imputed (observed/adjudicated).	Not applicable
Outras condições obstétricas e do parto/puerpério	Other obstetric, delivery, and puerperal conditions	Named obstetric, delivery, and puerperal conditions are retained individually. “Other” does not mean missing or a single disease. Not imputed (observed/adjudicated).	Not applicable
Recém-nascido	Gestational age at birth	Preterm <37 weeks; term 37 weeks to 41 weeks and 6 days; post-term ≥42 completed weeks. Conditional donor imputation.	Not applicable
Classificação do peso do recém-nascido	Birth weight classification	Five recorded weight groups. Additional binary contrasts: <2500 g and ≥4000 g. 2500–3999 g is normal birth weight, not synonymous with appropriate for gestational age. Conditional donor imputation.	Not applicable
Classificação quanto ao tamanho do recém-nascido	Birth weight for gestational age	Small for gestational age, appropriate for gestational age, and large for gestational age are separate recorded categories. Growth-reference validation is not possible from category-only data. Conditional donor imputation.	Not applicable
Apgar 5o minuto	Apgar score at 5 minutes	Numerical 5-minute Apgar retained. Not applicable is structural. An unpooled numerical-score global test is replaced by grouped-score comparison. Conditional donor imputation.	Not applicable
Apgar categorias	Apgar score category at 5 minutes	Low 0–3, moderately abnormal 4–6, reassuring 7–10. Low Apgar is not diagnosed as asphyxia. Derived from imputed components.	Not applicable
Desfecho perinatal adverso composto	Composite adverse perinatal outcome	Primary composite: NICU admission or fetal death or stillbirth or 5-minute Apgar <7. Each participant contributes at most one event. Derived from imputed components.	Not applicable

Source field (Portuguese)	Reporting term (English)	Coding or analysis treatment	Mapping to binary indicators
Unidade de tratamento intensivo ou óbito	NICU admission or death	Narrower composite: NICU admission or or fetal death or stillbirth, excluding low Apgar alone. Not imputed (observed/adjudicated).	Not applicable
Internação em unidade de tratamento intensivo neonatal	NICU admission	NICU entries in source Excel rows 21 and 940 were author-adjudicated as No before imputation, not statistically imputed. Not imputed (observed/adjudicated).	Not applicable
Óbito	Death	Fetal death or stillbirth is a binary exploratory component. Fetal-death and stillbirth labels were preserved as recorded and not treated as independent biological outcomes. Not imputed (observed/adjudicated).	Not applicable
Abortos	Previous pregnancy losses	No recorded loss, 1, 2, or 3 previous losses. A spontaneous or induced mechanism was not inferred. Not imputed (observed/adjudicated).	Not applicable

BMI, body mass index; HELLP, hemolysis, elevated liver enzymes, and low platelet count; NICU, neonatal intensive care unit. Missing values comprise blank, ignored, or not-informed entries. Explicit No, Absent, and Not applicable values are observed information. BMI was not imputed. All imputation and marker organization retained the separation between maternal clinical conditions and obstetric complications. Recorded combinations were retained as single descriptive categories. This did not prevent a participant from contributing once to each applicable binary indicator. Participants were counted only once within each indicator, and clinical and obstetric domains remained separate. The mapping of recorded diagnostic combinations to binary analysis indicators is presented in Table S1a.

Table S1a. Proposed mapping of recorded diagnostic combinations to binary analysis indicators.

Recorded diagnostic combination	Corresponding condition-specific or grouped binary indicators	Overall domain indicator
Ureterocele; hydronephrosis	Noninfectious renal and urological conditions: any recorded condition	Any maternal clinical condition
Cholelithiasis; pancreatitis	Gastrointestinal and hepatobiliary conditions and hernias: any recorded condition	Any maternal clinical condition
Epilepsy; syncope	Neurological conditions and other mental disorders: any recorded condition	Any maternal clinical condition
Rickets; scoliosis	Other specified clinical conditions and medical history: any recorded condition	Any maternal clinical condition
Hypertensive disorder of pregnancy; preeclampsia	Hypertensive disorders of pregnancy	Any obstetric complication
Hypertensive disorder of pregnancy; HELLP syndrome	Hypertensive disorders of pregnancy	Any obstetric complication
Fetal distress; sustained fetal tachycardia	Fetal distress; other fetal heart rate abnormalities; fetal status abnormalities	Any obstetric complication
Ventricular septal defect; congenital clubfoot	Fetal congenital anomalies: any recorded condition	Any obstetric complication

Each combination represents coexisting diagnoses recorded for the same participant. Combined labels were retained as single categories for descriptive reporting. For binary analyses, each participant contributed once to each applicable indicator listed in the table and only once to the corresponding overall domain indicator, irrespective of the number of diagnoses. Indicators were therefore not mutually exclusive, and their frequencies must not be summed to estimate the number of affected participants. Individual diagnoses within broad descriptive groups were not assigned separate analytical indicators unless such indicators were included in the analysis. Clinical and obstetric domains remained separate. HELLP, hemolysis, elevated liver enzymes, and low platelet count.

Table S2. Frequency of wider-pool donor selection during iterative conditional donor imputation.

Imputed variable or joint block	Total donor draws	Draws from the assigned terminal leaf	Draws from the wider compatible pool	Wider-pool draws (%)
Race	63,000	63,000	0	0.00
Marital status	111,000	111,000	0	0.00
Educational attainment	345,000	345,000	0	0.00
Mode of delivery	6000	5447	553	9.22
Episiotomy	16,330	16,330	0	0.00
Recorded surgery/labor duration	99,000	99,000	0	0.00
Perineal tear	25,330	25,330	0	0.00
Gravidity and parity	390,000	345,378	44,622	11.44
Gestational-age category at birth, birth-weight category, and birth weight for gestational age	42,000	35,979	6021	14.34
Numerical 5-minute Apgar score	36,000	36,000	0	0.00

Counts summarize iterative donor draws across 50 independently seeded chains and 60 iterations in the initial 1012-record dataset, before exclusion of twin pregnancies. Initial donor sampling is excluded. Wider-pool selection occurred when the assigned terminal leaf contained no compatible observed donor. Percentages were calculated as 100 × wider-pool draws / total donor draws. Counts describe repeated donor selections, not unique participants or unique donors.

Joint blocks contributed one donor draw per updated record and iteration, even when several missing components were replaced. Structural non-applicability assignments did not require donor sampling and were excluded from denominators. Across iterations, 1670 structural assignments were excluded from each perineal-variable denominator. Fallback counts were recovered from the original execution records; total donor-draw denominators were reconstructed from the missingness masks, update schedule, and saved delivery-route traces. Final-iteration-specific fallback counts were not recorded.

Neonatal intensive care unit admission and recorded death were not statistically imputed and therefore had no donor-draw denominator. The Apgar category and primary composite were derived from their components rather than independently imputed. These diagnostics describe the operation of the algorithm and do not establish the validity of the missing-at-random assumption.

Table S3. Variable-specific predictor sets and compatibility restrictions used in the conditional donor imputation algorithm.

Imputed variable or joint block	Tree model	Exact predictor set[1]	Eligible donors and compatibility restrictions
Race	Classification tree	*P* excluding column 3	Records with observed race. Donor values were restricted to observed categories; no additional compatibility restriction was imposed.
Marital status	Classification tree	*P* excluding column 5	Records with observed marital status. Donor values were restricted to observed categories; no additional compatibility restriction was imposed.
Educational attainment	Classification tree	*P* excluding column 6	Records with observed educational attainment. Donor values were restricted to observed categories; no additional compatibility restriction was imposed.
Mode of delivery	Classification tree	*P* excluding columns 28, 29, and 31	Records with observed delivery route. An observed applicable episiotomy or perineal-tear entry required vaginal delivery; an observed "not applicable" entry required cesarean delivery.
Episiotomy	Classification tree	*P* excluding column 29	For recipients classified as having vaginal delivery, donors were restricted to records with observed vaginal delivery and observed episiotomy. Cesarean deliveries received structural "not applicable" without a donor draw.
Recorded surgery/labor duration	Classification tree	*P* excluding column 30	Records with observed duration. Donor values were restricted to the recorded categories; no additional delivery-route compatibility restriction was imposed.
Perineal tear	Classification tree	*P* excluding column 31	For recipients classified as having vaginal delivery, donors were restricted to records with observed vaginal delivery and observed tear status. Cesarean deliveries received structural "not applicable" without a donor draw.
Gravidity and parity	Joint classification tree	*P* excluding columns 32 and 33	Donors had both variables observed and matched any observed recipient component. The upper bound of the gravidity category had to be at least the lower bound of the parity category plus the recipient's recorded previous pregnancy losses. Only missing components were replaced.
Gestational-age category at birth, birth-weight category, and birth weight for gestational age	Joint classification tree	*P* excluding columns 54, 55, and 56	Donors had all three variables observed and matched every observed recipient component. Missing components were copied jointly from one compatible donor, preserving an observed combination.
Numerical 5-minute Apgar score	Regression tree	*P* excluding columns 57 and 59	Donors had observed numeric Apgar scores. Missing scores were updated only for recipients without recorded death. Observed numeric scores and structural "not applicable" entries were preserved.

Column numbers refer to the original one-based Excel source-column positions. [1] *P* comprises source columns 2–57 and 59–63, together with indicators of twin status and original BMI missingness. The exact fields corresponding to these columns are listed below. Column 58, the derived Apgar category, was excluded from every predictor matrix. Each model used all predictors in P except the explicitly listed exclusions. For individual updates, the target variable was excluded; all jointly updated variables were excluded from their joint model. Perineal variables were additionally excluded from the delivery-route model, and the primary composite was excluded from numerical Apgar prediction.

Trees were fitted to bootstrap samples of eligible observed records using a minimum leaf size of five and a cost-complexity pruning parameter of 0.0001. Donors were sampled from compatible observed records in the recipient's assigned terminal leaf; when none were available, sampling used the wider compatible observed pool. Joint updates used one donor per recipient and iteration. Compatibility restrictions preserved observed recipient values and operated on the recorded categories rather than independently validated diagnoses.

Donor eligibility was not restricted by plurality status. Twin and singleton records could share donors when the other compatibility conditions were satisfied; twin status was included as a predictor but was not a mandatory matching criterion.

For the reproductive block, gravidity-category upper bounds were represented as 1, 2, and 999 for one, two, and at least three pregnancies, respectively; parity-category lower bounds were 1, 2, and 3. The value 999 was a computational upper-bound convention for the open-ended gravidity category, not an observed number of pregnancies.

BMI, body mass index; HELLP, hemolysis, elevated liver enzymes, and low platelet count; NICU, neonatal intensive care unit.

Exact definition of predictor set P

Source columns	Corresponding predictor fields
2–9	2: Recorded maternal BMI; 3: race; 4: maternal age; 5: marital status; 6: educational attainment; 7: smoking; 8: alcohol consumption; 9: illicit drug use.
10–18	10: Any maternal clinical condition; 11: chronic hypertension; 12: hypothyroidism; 13: urinary tract infection; 14: asthma; 15: pre-existing diabetes mellitus; 16: type of pre-existing diabetes mellitus; 17:syphilis; 18: depression.
19–27	19: Noninfectious renal and urological conditions; 20: other cardiovascular conditions; 21: hematological conditions and thrombophilias; 22: autoimmune conditions; 23: gastrointestinal and hepatobiliary conditions and hernias; 24: neurological conditions and other mental disorders; 25: gynecological conditions, neoplasms, and masses of undetermined nature; 26: other maternal infections; 27: other specified clinical conditions and medical history.
28–36	28: Mode of delivery; 29: episiotomy; 30: recorded surgery/labor duration; 31: perineal tear; 32: gravidity; 33: parity; 34: any obstetric complication; 35: gestational diabetes mellitus; 36: hypertensive disorders of pregnancy.
37–45	37: Recorded type of hypertensive disorder of pregnancy; 38: fetal growth abnormalities; 39: recorded type of fetal growth abnormality; 40: intrauterine growth restriction; 41: recorded fetal macrosomia; 42: fetal status abnormalities; 43: recorded type of fetal status abnormality; 44: fetal distress; 45: other fetal heart rate abnormalities.
46–53	46: Amniotic fluid abnormalities; 47: recorded type of amniotic fluid abnormality; 48: polyhydramnios; 49:oligohydramnios; 50: rupture of membranes; 51: fetal congenital anomalies; 52: placental abnormalities; 53:other obstetric, delivery, and puerperal conditions.
54–57	54: Gestational-age category at birth; 55: birth-weight category; 56: birth weight for gestational age; 57: numerical 5-minute Apgar score.
59–63	59: Primary composite adverse perinatal outcome; 60: NICU admission or death; 61: NICU admission; 62: recorded death; 63: previous pregnancy losses.
Additional indicators	Twin status and original BMI missingness.

Predictor encoding: Columns 19–27, 37, 39, 43, 45, 47, 51, 52, and 53 were represented as presence indicators: "absent" or missing was coded as zero, and other recorded categories as one. Other categorical predictors used one-hot encoding. Numerical Apgar was entered numerically, with nonnumeric entries represented by −1. BMI used indicators for observed categories plus an additional "BMI not recorded" category; a separate original BMI-missingness indicator was also included. These representations did not impute BMI or change its source values. The primary composite predictor was retained in models other than the numerical Apgar model, as implemented.

Table S4. Sensitivity analyses of maternal body mass index (BMI) associations using three maternal-age categories.

Condition or outcome	Overweight versus normal weight, aOR (95% CI)	Contrast *p*-Value	Obesity versus normal weight, aOR (95% CI)	Contrast *p*-Value	Global BMI *p*-Value
Any maternal clinical condition	1.58 (1.03–2.43)	0.038	2.91 (1.97–4.30)	<0.001	<0.001
Chronic hypertension	1.44 (0.55–3.77)	0.455	8.17 (3.76–17.75)	<0.001	<0.001
Any obstetric complication	2.09 (1.40–3.12)	<0.001	4.02 (2.75–5.86)	<0.001	<0.001
Gestational diabetes mellitus	1.73 (1.01–2.97)	0.046	2.21 (1.34–3.63)	0.002	0.008
Hypertensive disorders of pregnancy	1.85 (0.99–3.47)	0.053	6.38 (3.73–10.91)	<0.001	<0.001
Cesarean delivery	2.81 (1.83–4.30)	<0.001	3.06 (2.04–4.59)	<0.001	<0.001
Preterm birth (<37 weeks)	0.95 (0.58–1.55)	0.841	0.96 (0.61–1.53)	0.873	0.977
Low birth weight (<2500 g)	1.08 (0.65–1.79)	0.773	0.67 (0.39–1.13)	0.134	0.178
Large for gestational age	3.79 (1.63–8.82)	0.002	6.29 (2.85–13.87)	<0.001	<0.001

Analyses included 760 women with singleton pregnancies and recorded BMI in the normal-weight, overweight, or obesity categories. Each condition or outcome was analyzed in a separate logistic regression model adjusted for maternal age (<25, 25–34, and ≥35 years; reference: <25 years) and parity (≥2 versus 1 delivery, including the index delivery). Normal weight was the reference BMI category. Models included five nonintercept coefficients: two for BMI, two for age, and one for parity. All nine models converged in all 50 completed datasets. Coefficient estimates and covariance matrices were pooled using Rubin's rules. Individual-contrast *p*-Values and 95% confidence intervals used pooled t inference with Barnard–Rubin degrees of freedom. Global BMI *p*-Values used the pooled D1 multivariate Wald test with a finite-sample correction. Maternal BMI was not imputed. The age specification was the only change from the corresponding core models in Table 4. Analyses were exploratory and unadjusted for multiple comparisons. aOR, adjusted odds ratio; BMI, body mass index; CI, confidence interval.

Table S5. Variable-level missing data before multiple imputation according to the availability of maternal body mass index (BMI) among 999 singleton pregnancies.
Values are *n* (%) of missing observations, using the full group size as the denominator. BMI itself is missing in 212/999 participants (21.2%) and was not imputed.

Markers	BMI recorded (*N* = 787)	BMI missing (*N* = 212)
**Sociodemographic characteristics and lifestyle**		
Race	14 (1.8%)	6 (2.8%)
Maternal age (years)	0 (0.0%)	0 (0.0%)
Marital status	27 (3.4%)	10 (4.7%)
Educational attainment	82 (10.4%)	30 (14.2%)
Smoking	0 (0.0%)	0 (0.0%)
Alcohol consumption	0 (0.0%)	0 (0.0%)
Illicit drug use	0 (0.0%)	0 (0.0%)
**Maternal clinical conditions**		
Maternal clinical conditions	0 (0.0%)	0 (0.0%)
Chronic hypertension	0 (0.0%)	0 (0.0%)
Hypothyroidism	0 (0.0%)	0 (0.0%)
Urinary tract infection	0 (0.0%)	0 (0.0%)
Asthma	0 (0.0%)	0 (0.0%)
Pre-existing diabetes mellitus	0 (0.0%)	0 (0.0%)
Type of pre-existing diabetes mellitus	0 (0.0%)	0 (0.0%)
Syphilis	0 (0.0%)	0 (0.0%)
Depression	0 (0.0%)	0 (0.0%)
Noninfectious renal and urological conditions	0 (0.0%)	0 (0.0%)
Other cardiovascular conditions	0 (0.0%)	0 (0.0%)
Hematological conditions and thrombophilias	0 (0.0%)	0 (0.0%)
Autoimmune conditions	0 (0.0%)	0 (0.0%)
Gastrointestinal and hepatobiliary conditions and hernias	0 (0.0%)	0 (0.0%)
Neurological conditions and other mental disorders	0 (0.0%)	0 (0.0%)
Gynecological conditions, neoplasms, and masses of undetermined nature	0 (0.0%)	0 (0.0%)
Other maternal infections	0 (0.0%)	0 (0.0%)
Other specified clinical conditions and medical history	0 (0.0%)	0 (0.0%)
**Delivery and reproductive history**		
Mode of delivery	2 (0.3%)	0 (0.0%)
Episiotomy	4 (0.5%)	2 (0.9%)
Recorded duration of surgery or labor (minutes)	27 (3.4%)	6 (2.8%)
Perineal tear at vaginal delivery	7 (0.9%)	2 (0.9%)
Gravidity	81 (10.3%)	31 (14.6%)
Parity	15 (1.9%)	6 (2.8%)
**Obstetric and fetal conditions**		
Obstetric complications	0 (0.0%)	0 (0.0%)
Gestational diabetes mellitus	0 (0.0%)	0 (0.0%)
Hypertensive disorders of pregnancy	0 (0.0%)	0 (0.0%)
Hypertensive disorders of pregnancy: recorded type	0 (0.0%)	0 (0.0%)
Fetal growth abnormalities	0 (0.0%)	0 (0.0%)
Fetal growth abnormalities: recorded type	0 (0.0%)	0 (0.0%)
Intrauterine growth restriction	0 (0.0%)	0 (0.0%)
Fetal macrosomia	0 (0.0%)	0 (0.0%)
Fetal status abnormalities	0 (0.0%)	0 (0.0%)
Fetal status abnormalities: recorded type	0 (0.0%)	0 (0.0%)
Fetal distress	0 (0.0%)	0 (0.0%)
Other fetal heart rate abnormalities	0 (0.0%)	0 (0.0%)
Amniotic fluid abnormalities	0 (0.0%)	0 (0.0%)
Amniotic fluid abnormalities: recorded type	0 (0.0%)	0 (0.0%)
Polyhydramnios	0 (0.0%)	0 (0.0%)
Oligohydramnios	0 (0.0%)	0 (0.0%)
Rupture of membranes	0 (0.0%)	0 (0.0%)

Fetal congenital anomalies	0 (0.0%)	0 (0.0%)
Placental abnormalities	0 (0.0%)	0 (0.0%)
Other obstetric, delivery, and puerperal conditions	0 (0.0%)	0 (0.0%)
**Perinatal characteristics and outcomes**		
Gestational age at birth	7 (0.9%)	1 (0.5%)
Birth weight classification	9 (1.1%)	0 (0.0%)
Birth weight for gestational age	4 (0.5%)	0 (0.0%)
Apgar score at 5 minutes	6 (0.8%)	5 (2.4%)
Apgar score category at 5 minutes	6 (0.8%)	5 (2.4%)
Composite adverse perinatal outcome	6 (0.8%)	3 (1.4%)
NICU admission or death	0 (0.0%)	0 (0.0%)
NICU admission	0 (0.0%)	0 (0.0%)
Death	0 (0.0%)	0 (0.0%)
Previous pregnancy losses	0 (0.0%)	0 (0.0%)

BMI, body mass index; NICU, neonatal intensive care unit. n, category frequency or number of events, as specified; *N*, group size or analysis denominator. Missing observations comprise blank cells, ignored values, and values recorded as not informed. Absent, no, and not applicable are observed categories and are not classified as missing.

All 999 singleton records are included in this comparison regardless of the availability of other markers. The table uses the corrected database before imputation. Participants with missing BMI remain excluded from analyses requiring an observed BMI category.

Table S6. Full observed-data description and bivariate comparisons by recorded maternal body mass index (BMI) before multiple imputation.
Values are *n* (%). Maternal BMI was never imputed. Descriptive columns retain all 787 singleton pregnancies with recorded BMI. *p*-Values compare only normal weight, overweight, and obesity (*N* = 760). Underweight was excluded from association analyses because the subgroup was small and had no composite event. Percentages were calculated before rounding. Category percentages exclude missing data; missing-data percentages use the full group denominator. Perineal global *p*-Values are restricted to vaginal deliveries.

**Markers**	**Underweight** *N* **= 27**	**Normal weight** *N* **= 282**	**Overweight** *N* **= 210**	**Obesity** *N* **= 268**	**Total** *N* **= 787**	***p*-Value**
**Sociodemographic characteristics and lifestyle**						
**Race**	**Valid *n* = 26**	**Valid *n* = 275**	**Valid *n* = 207**	**Valid *n* = 265**	**Valid *n* = 773**	**0.659**#
White	19 (73.1%)	190 (69.1%)	154 (74.4%)	185 (69.8%)	548 (70.9%)	
Mixed race	6 (23.1%)	71 (25.8%)	45 (21.7%)	64 (24.2%)	186 (24.1%)	
Black	1 (3.8%)	14 (5.1%)	8 (3.9%)	16 (6.0%)	39 (5.0%)	
Missing data	1 (3.7%)	7 (2.5%)	3 (1.4%)	3 (1.1%)	14 (1.8%)	
**Maternal age (years)**	**Valid *n* = 27**	**Valid *n* = 282**	**Valid *n* = 210**	**Valid *n* = 268**	**Valid *n* = 787**	**<0.001**†
15-19	8 (29.6%)	47 (16.7%)	14 (6.7%)	10 (3.7%)	79 (10.0%)	
20-24	10 (37.0%)	77 (27.3%)	48 (22.9%)	55 (20.5%)	190 (24.1%)	
25-29	3 (11.1%)	65 (23.0%)	51 (24.3%)	80 (29.9%)	199 (25.3%)	
30-34	6 (22.2%)	45 (16.0%)	49 (23.3%)	60 (22.4%)	160 (20.3%)	
35-39	0 (0.0%)	34 (12.1%)	38 (18.1%)	44 (16.4%)	116 (14.7%)	
40-44	0 (0.0%)	14 (5.0%)	8 (3.8%)	18 (6.7%)	40 (5.1%)	
45-49	0 (0.0%)	0 (0.0%)	2 (1.0%)	1 (0.4%)	3 (0.4%)	
**Marital status**	**Valid *n* = 27**	**Valid *n* = 270**	**Valid *n* = 206**	**Valid *n* = 257**	**Valid *n* = 760**	**0.231**#
Married	5 (18.5%)	90 (33.3%)	77 (37.4%)	109 (42.4%)	281 (37.0%)	
Divorced	0 (0.0%)	5 (1.9%)	6 (2.9%)	7 (2.7%)	18 (2.4%)	
Single	21 (77.8%)	159 (58.9%)	106 (51.5%)	129 (50.2)	415 (54.6%)	
Stable union	1 (3.7%)	16 (5.9%)	17 (8.3%)	12 (4.7%)	46 (6.1%)	
Missing data	0 (0.0%)	12 (4.3%)	4 (1.9%)	11 (4.1%)	27 (3.4%)	
**Educational attainment**	**Valid *n* = 22**	**Valid *n* = 258**	**Valid *n* = 186**	**Valid *n* = 239**	**Valid *n* = 705**	**0.540**†
Illiterate	0 (0.0%)	0 (0.0%)	1 (0.5%)	0 (0.0%)	1 (0.1%)	
Incomplete elementary school	1 (4.5%)	15 (5.8%)	16 (8.6%)	22 (9.2%)	54 (7.7%)	
Completed elementary school	5 (22.7%)	36 (14.0%)	27 (14.5%)	47 (19.7%)	115 (16.3%)	
Incomplete high school	3 (13.6%)	23 (8.9%)	18 (9.7%)	19 (7.9%)	63 (8.9%)	
Completed high school	11 (50.0%)	144 (55.8%)	101 (54.3%)	117 (49.0%)	373 (52.9%)	
Incomplete higher education	0 (0.0%)	11 (4.3%)	4 (2.2%)	10 (4.2%)	25 (3.5%)	
Completed higher education	2 (9.1%)	29 (11.2%)	19 (10.2%)	23 (9.6%)	73 (10.4%)	
Doctorate/PhD	0 (0.0%)	0 (0.0%)	0 (0.0%)	1 (0.4%)	1 (0.1%)	
Missing data	5 (18.5%)	24 (8.5%)	24 (11.4%)	29 (10.8%)	82 (10.4%)	
**Smoking**	**Valid *n* = 27**	**Valid *n* = 282**	**Valid *n* = 210**	**Valid *n* = 268**	**Valid *n* = 787**	**0.426**#
No	24 (88.9%)	258 (91.5%)	198 (94.3%)	251 (93.7%)	731 (92.9%)	
Yes	3 (11.1%)	24 (8.5%)	12 (5.7%)	17 (6.3%)	56 (7.1%)	
**Alcohol consumption**	**Valid *n* = 27**	**Valid *n* = 282**	**Valid *n* = 210**	**Valid *n* = 268**	**Valid *n* = 787**	**0.135**#
No	24 (88.9%)	270 (95.7%)	207 (98.6%)	262 (97.8%)	763 (97.0%)	
Yes	3 (11.1%)	12 (4.3%)	3 (1.4%)	6 (2.2%)	24 (3.0%)	
**Illicit drug use**	**Valid *n* = 27**	**Valid *n* = 282**	**Valid *n* = 210**	**Valid *n* = 268**	**Valid *n* = 787**	**0.190**†
No	25 (92.6%)	277 (98.2%)	209 (99.5%)	267 (99.6%)	778 (98.9%)	
Yes	2 (7.4%)	5 (1.8%)	1 (0.5%)	1 (0.4%)	9 (1.1%)	
**Maternal clinical conditions**						
**Maternal clinical conditions (any)**	**Valid *n* = 27**	**Valid *n* = 282**	**Valid *n* = 210**	**Valid *n* = 268**	**Valid *n* = 787**	**<0.001**#
Absent	24 (88.9%)	227 (80.5%)	151 (71.9%)	155 (57.8%)	557 (70.8%)	
Present	3 (11.1%)	55 (19.5%)	59 (28.1%)	113 (42.2%)	230 (29.2%)	
**Chronic hypertension**	**Valid *n* = 27**	**Valid *n* = 282**	**Valid *n* = 210**	**Valid *n* = 268**	**Valid *n* = 787**	**<0.001**#
Absent	27 (100.0%)	274 (97.2%)	200 (95.2%)	210 (78.4%)	711 (90.3%)	
Present	0 (0.0%)	8 (2.8%)	10 (4.8%)	58 (21.6%)	76 (9.7%)	
**Hypothyroidism**	**Valid *n* = 27**	**Valid *n* = 282**	**Valid *n* = 210**	**Valid *n* = 268**	**Valid *n* = 787**	**0.059**#
Absent	25 (92.6%)	271 (96.1%)	199 (94.8%)	245 (91.4%)	740 (94.0%)	
Present	2 (7.4%)	11 (3.9%)	11 (5.2%)	23 (8.6%)	47 (6.0%)	

**Markers**	**Underweight** *N* = 27	**Normal weight** *N* = 282	**Overweight** *N* = 210	**Obesity** *N* = 268	**Total** *N* = 787	***p*-Value**
**Urinary tract infection**	**Valid *n* = 27**	**Valid *n* = 282**	**Valid *n* = 210**	**Valid *n* = 268**	**Valid *n* = 787**	**0.352#**
Absent	26 (96.3%)	273 (96.8%)	198 (94.3%)	258 (96.3%)	755 (95.9%)	
Present	1 (3.7%)	9 (3.2%)	12 (5.7%)	10 (3.7%)	32 (4.1%)	
**Asthma**	**Valid *n* = 27**	**Valid *n* = 282**	**Valid *n* = 210**	**Valid *n* = 268**	**Valid *n* = 787**	**0.554#**
Absent	27 (100.0%)	274 (97.2%)	201 (95.7%)	256 (95.5%)	758 (96.3%)	
Present	0 (0.0%)	8 (2.8%)	9 (4.3%)	12 (4.5%)	29 (3.7%)	
**Pre-existing diabetes mellitus**	**Valid *n* = 27**	**Valid *n* = 282**	**Valid *n* = 210**	**Valid *n* = 268**	**Valid *n* = 787**	**0.197†**
Absent	27 (100.0%)	280 (99.3%)	205 (97.6%)	261 (97.4%)	773 (98.2%)	
Present	0 (0.0%)	2 (0.7%)	5 (2.4%)	7 (2.6%)	14 (1.8%)	
**Type of pre-existing diabetes mellitus**	**Valid *n* = 27**	**Valid *n* = 282**	**Valid *n* = 210**	**Valid *n* = 268**	**Valid *n* = 787**	**0.559†**
Absent	27 (100.0%)	280 (99.3%)	205 (97.6%)	261 (97.4%)	773 (98.2%)	
Type 1 diabetes mellitus	0 (0.0%)	0 (0.0%)	1 (0.5%)	1 (0.4%)	2 (0.3%)	
Type 2 diabetes mellitus	0 (0.0%)	2 (0.7%)	4 (1.9%)	6 (2.2%)	12 (1.5%)	
**Syphilis**	**Valid *n* = 27**	**Valid *n* = 282**	**Valid *n* = 210**	**Valid *n* = 268**	**Valid *n* = 787**	**0.639†**
Absent	27 (100.0%)	278 (98.6%)	209 (99.5%)	266 (99.3%)	780 (99.1%)	
Present	0 (0.0%)	4 (1.4%)	1 (0.5%)	2 (0.7%)	7 (0.9%)	
**Depression**	**Valid *n* = 27**	**Valid *n* = 282**	**Valid *n* = 210**	**Valid *n* = 268**	**Valid *n* = 787**	**0.007†**
Absent	27 (100.0%)	282 (100.0%)	204 (97.1%)	266 (99.3%)	779 (99.0%)	
Present	0 (0.0%)	0 (0.0%)	6 (2.9%)	2 (0.7%)	8 (1.0%)	
**Noninfectious renal and urological conditions**	**Valid *n* = 27**	**Valid *n* = 282**	**Valid *n* = 210**	**Valid *n* = 268**	**Valid *n* = 787**	**0.061†**
Absent	27 (100.0%)	280 (99.3%)	207 (98.6%)	267 (99.6%)	781 (99.2%)	
Renal atrophy	0 (0.0%)	1 (0.4%)	0 (0.0%)	0 (0.0%)	1 (0.1%)	
Glomerulonephritis	0 (0.0%)	0 (0.0%)	1 (0.5%)	0 (0.0%)	1 (0.1%)	
Hydronephrosis	0 (0.0%)	0 (0.0%)	0 (0.0%)	1 (0.4%)	1 (0.1%)	
Nephrolithiasis	0 (0.0%)	0 (0.0%)	2 (1.0%)	0 (0.0%)	2 (0.3%)	
Ureterocele; hydronephrosis	0 (0.0%)	1 (0.4%)	0 (0.0%)	0 (0.0%)	1 (0.1%)	
**Other cardiovascular conditions**	**Valid *n* = 27**	**Valid *n* = 282**	**Valid *n* = 210**	**Valid *n* = 268**	**Valid *n* = 787**	**0.473†**
Absent	27 (100.0%)	279 (98.9%)	209 (99.5%)	266 (99.3%)	781 (99.2%)	
Arrhythmia	0 (0.0%)	1 (0.4%)	0 (0.0%)	0 (0.0%)	1 (0.1%)	
Hypertrophic cardiomyopathy	0 (0.0%)	0 (0.0%)	0 (0.0%)	1 (0.4%)	1 (0.1%)	
Unspecified heart disease	0 (0.0%)	2 (0.7%)	0 (0.0%)	0 (0.0%)	2 (0.3%)	
Mitral valve prolapse	0 (0.0%)	0 (0.0%)	1 (0.5%)	1 (0.4%)	2 (0.3%)	
**Hematological conditions and thrombophilias**	**Valid *n* = 27**	**Valid *n* = 282**	**Valid *n* = 210**	**Valid *n* = 268**	**Valid *n* = 787**	**0.751†**
Absent	26 (96.3%)	278 (98.6%)	207 (98.6%)	267 (99.6%)	778 (98.9%)	
Anemia	1 (3.7%)	2 (0.7%)	2 (1.0%)	1 (0.4%)	6 (0.8%)	
Spherocytosis	0 (0.0%)	1 (0.4%)	0 (0.0%)	0 (0.0%)	1 (0.1%)	
Sickle cell trait	0 (0.0%)	1 (0.4%)	0 (0.0%)	0 (0.0%)	1 (0.1%)	
Hereditary thrombophilia associated with factor V Leiden	0 (0.0%)	0 (0.0%)	1 (0.5%)	0 (0.0%)	1 (0.1%)	
**Autoimmune conditions**	**Valid *n* = 27**	**Valid *n* = 282**	**Valid *n* = 210**	**Valid *n* = 268**	**Valid *n* = 787**	**0.102†**
Absent	27 (100.0%)	282 (100.0%)	208 (99.0%)	267 (99.6%)	784 (99.6%)	
Rheumatoid arthritis	0 (0.0%)	0 (0.0%)	0 (0.0%)	1 (0.4%)	1 (0.1%)	
Unspecified autoimmune skin disease	0 (0.0%)	0 (0.0%)	1 (0.5%)	0 (0.0%)	1 (0.1%)	
Systemic lupus erythematosus	0 (0.0%)	0 (0.0%)	1 (0.5%)	0 (0.0%)	1 (0.1%)	
**Gastrointestinal and hepatobiliary conditions and hernias**	**Valid *n* = 27**	**Valid *n* = 282**	**Valid *n* = 210**	**Valid *n* = 268**	**Valid *n* = 787**	**0.706†**
Absent	27 (100.0%)	280 (99.3%)	209 (99.5%)	264 (98.5%)	780 (99.1%)	
Cholecystitis	0 (0.0%)	0 (0.0%)	0 (0.0%)	1 (0.4%)	1 (0.1%)	
Cholelithiasis; pancreatitis	0 (0.0%)	0 (0.0%)	0 (0.0%)	1 (0.4%)	1 (0.1%)	
Crohn disease	0 (0.0%)	1 (0.4%)	0 (0.0%)	0 (0.0%)	1 (0.1%)	
Gastroesophageal reflux disease	0 (0.0%)	1 (0.4%)	0 (0.0%)	0 (0.0%)	1 (0.1%)	
Gastritis	0 (0.0%)	0 (0.0%)	1 (0.5%)	0 (0.0%)	1 (0.1%)	
Hiatal hernia	0 (0.0%)	0 (0.0%)	0 (0.0%)	1 (0.4%)	1 (0.1%)	
Umbilical hernia	0 (0.0%)	0 (0.0%)	0 (0.0%)	1 (0.4%)	1 (0.1%)	
**Neurological conditions and other mental disorders**	**Valid *n* = 27**	**Valid *n* = 282**	**Valid *n* = 210**	**Valid *n* = 268**	**Valid *n* = 787**	**0.387†**

Markers	Underweight *N* = 27	Normal weight *N* = 282	Overweight *N* = 210	Obesity *N* = 268	Total *N* = 787	*p*-Value
Absent	27 (100.0%)	281 (99.6%)	207 (98.6%)	265 (98.9%)	780 (99.1%)	
Anxiety	0 (0.0%)	0 (0.0%)	0 (0.0%)	1 (0.4%)	1 (0.1%)	
Epilepsy	0 (0.0%)	1 (0.4%)	0 (0.0%)	1 (0.4%)	2 (0.3%)	
Epilepsy; syncope	0 (0.0%)	0 (0.0%)	1 (0.5%)	0 (0.0%)	1 (0.1%)	
Labyrinthitis	0 (0.0%)	0 (0.0%)	0 (0.0%)	1 (0.4%)	1 (0.1%)	
Bipolar disorder	0 (0.0%)	0 (0.0%)	1 (0.5%)	0 (0.0%)	1 (0.1%)	
Panic disorder	0 (0.0%)	0 (0.0%)	1 (0.5%)	0 (0.0%)	1 (0.1%)	
**Gynecological conditions, neoplasms, and masses of undetermined nature**	**Valid *n* = 27**	**Valid *n* = 282**	**Valid n = 210**	**Valid *n* = 268**	**Valid *n* = 787**	**0.835†**
Absent	27 (100.0%)	279 (98.9%)	209 (99.5%)	265 (98.9%)	780 (99.1%)	
Ovarian cyst	0 (0.0%)	1 (0.4%)	0 (0.0%)	0 (0.0%)	1 (0.1%)	
Uterine leiomyomatosis	0 (0.0%)	1 (0.4%)	0 (0.0%)	1 (0.4%)	2 (0.3%)	
Cervical intraepithelial neoplasia	0 (0.0%)	1 (0.4%)	0 (0.0%)	0 (0.0%)	1 (0.1%)	
Ovarian tumor of unspecified nature	0 (0.0%)	0 (0.0%)	0 (0.0%)	1 (0.4%)	1 (0.1%)	
Mass in the bladder region	0 (0.0%)	0 (0.0%)	0 (0.0%)	1 (0.4%)	1 (0.1%)	
Bicornuate uterus	0 (0.0%)	0 (0.0%)	1 (0.5%)	0 (0.0%)	1 (0.1%)	
**Other maternal infections**	**Valid *n* = 27**	**Valid *n* = 282**	**Valid *n* = 210**	**Valid *n* = 268**	**Valid *n* = 787**	**0.277†**
Absent	27 (100.0%)	280 (99.3%)	205 (97.6%)	264 (98.5%)	776 (98.6%)	
Perianal condyloma acuminatum	0 (0.0%)	0 (0.0%)	1 (0.5%)	0 (0.0%)	1 (0.1%)	
Hepatitis B	0 (0.0%)	1 (0.4%)	0 (0.0%)	0 (0.0%)	1 (0.1%)	
Hepatitis C	0 (0.0%)	1 (0.4%)	0 (0.0%)	0 (0.0%)	1 (0.1%)	
HIV infection	0 (0.0%)	0 (0.0%)	2 (1.0%)	1 (0.4%)	3 (0.4%)	
Pneumonia	0 (0.0%)	0 (0.0%)	0 (0.0%)	1 (0.4%)	1 (0.1%)	
Toxoplasmosis	0 (0.0%)	0 (0.0%)	2 (1.0%)	2 (0.7%)	4 (0.5%)	
**Other specified clinical conditions and medical history**	**Valid *n* = 27**	**Valid *n* = 282**	**Valid *n* = 210**	**Valid *n* = 268**	**Valid *n* = 787**	**0.777†**
Absent	27 (100.0%)	279 (98.9%)	208 (99.0%)	265 (98.9%)	779 (99.0%)	
Previous surgery	0 (0.0%)	0 (0.0%)	1 (0.5%)	2 (0.7%)	3 (0.4%)	
Fibromyalgia	0 (0.0%)	0 (0.0%)	1 (0.5%)	1 (0.4%)	2 (0.3%)	
Emotional lability	0 (0.0%)	1 (0.4%)	0 (0.0%)	0 (0.0%)	1 (0.1%)	
Rickets; scoliosis	0 (0.0%)	1 (0.4%)	0 (0.0%)	0 (0.0%)	1 (0.1%)	
Allergic rhinitis	0 (0.0%)	1 (0.4%)	0 (0.0%)	0 (0.0%)	1 (0.1%)	
**Delivery and reproductive history**						
**Mode of delivery**	**Valid *n* = 27**	**Valid *n* = 281**	**Valid *n* = 209**	**Valid *n* = 268**	**Valid *n* = 785**	**<0.001#**
Cesarean delivery	19 (70.4%)	170 (60.5%)	170 (81.3%)	221 (82.5%)	580 (73.9%)	
Vaginal delivery	8 (29.6%)	111 (39.5%)	39 (18.7%)	47 (17.5%)	205 (26.1%)	
Missing data	0 (0.0%)	1 (0.4%)	1 (0.5%)	0 (0.0%)	2 (0.3%)	
**Episiotomy**	**Valid *n* = 27**	**Valid *n* = 281**	**Valid *n* = 210**	**Valid *n* = 265**	**Valid *n* = 783**	**0.118#**
No	6 (22.2%)	67 (23.8%)	19 (9.0%)	30 (11.3%)	122 (15.6%)	
Yes	2 (7.4%)	44 (15.7%)	21 (10.0%)	14 (5.3%)	81 (10.3%)	
Not applicable	19 (70.4%)	170 (60.5%)	170 (81.0%)	221 (83.4%)	580 (74.1%)	
Missing data	0 (0.0%)	1 (0.4%)	0 (0.0%)	3 (1.1%)	4 (0.5%)	
**Recorded duration of surgery or labor (minutes)**	**Valid *n* = 27**	**Valid *n* = 266**	**Valid *n* = 208**	**Valid *n* = 259**	**Valid *n* = 760**	**0.002#**
<30	2 (7.4%)	22 (8.3%)	8 (3.8%)	12 (4.6%)	44 (5.8%)	
30–60	21 (77.8%)	154 (57.9%)	114 (54.8%)	129 (49.8%)	418 (55.0%)	
60–120	4 (14.8%)	87 (32.7%)	83 (39.9%)	103 (39.8%)	277 (36.4%)	
>120	0 (0.0%)	3 (1.1%)	3 (1.4%)	15 (5.8%)	21 (2.8%)	
Missing data	0 (0.0%)	16 (5.7%)	2 (1.0%)	9 (3.4%)	27 (3.4%)	
**Perineal tear at vaginal delivery**	**Valid *n* = 27**	**Valid *n* = 280**	**Valid *n* = 209**	**Valid *n* = 264**	**Valid *n* = 780**	**0.144†**
No tear	6 (22.2%)	83 (29.6%)	32 (15.3%)	25 (9.5%)	146 (18.7%)	
First degree	1 (3.7%)	15 (5.4%)	3 (1.4%)	10 (3.8%)	29 (3.7%)	
Second degree	1 (3.7%)	12 (4.3%)	4 (1.9%)	8 (3.0%)	25 (3.2%)	
Not applicable	19 (70.4%)	170 (60.7%)	170 (81.3%)	221 (83.7%)	580 (74.4%)	
Missing data	0 (0.0%)	2 (0.7%)	1 (0.5%)	4 (1.5%)	7 (0.9%)	
**Gravidity**	**Valid *n* = 26**	**Valid *n* = 247**	**Valid *n* = 187**	**Valid *n* = 246**	**Valid *n* = 706**	**<0.001#**
Primigravida (1 pregnancy)	18 (69.2%)	127 (51.4%)	60 (32.1%)	85 (34.6%)	290 (41.1%)	
Secundigravida (2 pregnancies)	2 (7.7%)	47 (19.0%)	39 (20.9%)	50 (20.3%)	138 (19.5%)	

Markers	Underweight *N* = 27	Normal weight *N* = 282	Overweight *N* = 210	Obesity *N* = 268	Total *N* = 787	*p*-Value
Multigravida (≥3 pregnancies)	6 (23.1%)	73 (29.6%)	88 (47.1%)	111 (45.1%)	278 (39. %4)	
Missing data	1 (3.7%)	35 (12.4%)	23 (11.0%)	22 (8.2%)	81 (10.3%)	
**Parity**	**Valid *n* = 27**	**Valid *n* = 276**	**Valid *n* = 209**	**Valid *n* = 260**	**Valid *n* = 772**	**<0.001#**
1 delivery	19 (70.4%)	155 (56.2%)	78 (37.3%)	97 (37.3%)	349 (45.2%)	
2 deliveries	6 (22.2%)	54 (19.6%)	49 (23.4%)	59 (22.7%)	168 (21.8%)	
≥3 deliveries	2 (7.4%)	67 (24.3%)	82 (39.2%)	104 (40.0%)	255 (33.0%)	
Missing data	0 (0.0%)	6 (2.1%)	1 (0.5%)	8 (3.0%)	15 (1.9%)	
**Obstetric and fetal conditions**						
**Obstetric complications**	**Valid *n* = 27**	**Valid *n* = 282**	**Valid *n* = 210**	**Valid *n* = 268**	**Valid *n* = 787**	**<0.001#**
Absent	20 (74.1%)	218 (77.3%)	129 (61.4%)	121 (45.1%)	488 (62.0%)	
Present	7 (25.9%)	64 (22.7%)	81 (38.6%)	147 (54.9%)	299 (38.0%)	
**Gestational diabetes mellitus**	**Valid *n* = 27**	**Valid *n* = 282**	**Valid n = 210**	**Valid *n* = 268**	**Valid *n* = 787**	**<0.001#**
Absent	26 (96.3%)	254 (90.1%)	173 (82.4%)	209 (78.0%)	662 (84.1%)	
Present	1 (3.7%)	28 (9.9%)	37 (17.6%)	59 (22.0%)	125 (15.9%)	
**Hypertensive disorders of pregnancy**	**Valid *n* = 27**	**Valid *n* = 282**	**Valid *n* = 210**	**Valid *n* = 268**	**Valid *n* = 787**	**<0.001#**
Absent	26 (96.3%)	262 (92.9%)	185 (88.1%)	184 (68.7%)	657 (83.5%)	
Present	1 (3.7%)	20 (7.1%)	25 (11.9%)	84 (31.3%)	130 (16.5%)	
**Hypertensive disorders of pregnancy: recorded type**	**Valid *n* = 27**	**Valid *n* = 282**	**Valid *n* = 210**	**Valid *n* = 268**	**Valid *n* = 787**	**<0.001†**
Absent	26 (96.3%)	262 (92.9%)	185 (88.1%)	184 (68.7%)	657 (83.5%)	
Hypertensive disorder of pregnancy	0 (0.0%)	18 (6.4%)	24 (11.4%)	82 (30.6%)	124 (15.8%)	
Hypertensive disorder of pregnancy; preeclampsia	0 (0.0%)	0 (0.0%)	0 (0.0%)	1 (0.4%)	1 (0.1%)	
Hypertensive disorder of pregnancy; HELLP syndrome	0 (0.0%)	1 (0.4%)	0 (0.0%)	1 (0.4%)	2 (0.3%)	
Eclampsia	1 (3.7%)	0 (0.0%)	0 (0.0%)	0 (0.0%)	1 (0.1%)	
Preeclampsia	0 (0.0%)	1 (0.4%)	1 (0.5%)	0 (0.0%)	2 (0.3%)	
**Fetal growth abnormalities**	**Valid *n* = 27**	**Valid *n* = 282**	**Valid *n* = 210**	**Valid *n* = 268**	**Valid *n* = 787**	**0.047†**
Absent	26 (96.3%)	282 (100.0%)	207 (98.6%)	262 (97.8%)	777 (98.7%)	
Present	1 (3.7%)	0 (0.0%)	3 (1.4%)	6 (2.2%)	10 (1.3%)	
**Fetal growth abnormalities: recorded type**	**Valid *n* = 27**	**Valid *n* = 282**	**Valid *n* = 210**	**Valid *n* = 268**	**Valid *n* = 787**	**0.031†**
Absent	26 (96.3%)	282 (100.0%)	207 (98.6%)	262 (97.8%)	777 (98.7%)	
Fetal macrosomia	0 (0.0%)	0 (0.0%)	0 (0.0%)	3 (1.1%)	3 (0.4%)	
Intrauterine growth restriction	1 (3.7%)	0 (0.0%)	3 (1.4%)	3 (1.1%)	7 (0.9%)	
**Intrauterine growth restriction**	**Valid *n* = 27**	**Valid *n* = 282**	**Valid *n* = 210**	**Valid *n* = 268**	**Valid *n* = 787**	**0.138†**
Absent	26 (96.3%)	282 (100.0%)	207 (98.6%)	265 (98.9%)	780 (99.1%)	
Present	1 (3.7%)	0 (0.0%)	3 (1.4%)	3 (1.1%)	7 (0.9%)	
**Fetal macrosomia**	**Valid *n* = 27**	**Valid *n* = 282**	**Valid *n* = 210**	**Valid *n* = 268**	**Valid *n* = 787**	**0.066†**
Absent	27 (100.0%)	282 (100.0%)	210 (100.0%)	265 (98.9%)	784 (99.6%)	
Present	0 (0.0%)	0 (0.0%)	0 (0.0%)	3 (1.1%)	3 (0.4%)	
**Fetal status abnormalities**	**Valid *n* = 27**	**Valid *n* = 282**	**Valid *n* = 210**	**Valid *n* = 268**	**Valid *n* = 787**	**0.056#**
Absent	25 (92.6%)	271 (96.1)	204 (97.1%)	266 (99.3%)	766 (97.3%)	
Present	2 (7.4%)	11 (3.9)	6 (2.9%)	2 (0.7%)	21 (2.7%)	
**Fetal status abnormalities: recorded type**	**Valid *n* = 27**	**Valid *n* = 282**	**Valid *n* = 210**	**Valid *n* = 268**	**Valid *n* = 787**	**0.071†**
Absent	25 (92.6%)	271 (96.1%)	204 (97.1%)	266 (99.3%)	766 (97.3%)	
Variable fetal heart rate decelerations	0 (0.0%)	0 (0.0%)	1 (0.5%)	0 (0.0%)	1 (0.1%)	
Fetal distress	1 (3.7%)	10 (3.5%)	5 (2.4%)	2 (0.7%)	18 (2.3%)	
Fetal distress; sustained fetal tachycardia	1 (3.7%)	0 (0.0%)	0 (0.0%)	0 (0.0%)	1 (0.1%)	
Fetal tachycardia	0 (0.0%)	1 (0.4%)	0 (0.0%)	0 (0.0%)	1 (0.1%)	
**Fetal distress**	**Valid *n* = 27**	**Valid *n* = 282**	**Valid *n* = 210**	**Valid *n* = 268**	**Valid *n* = 787**	**0.080†**
Absent	25 (92.6%)	272 (96.5%)	205 (97.6%)	266 (99.3%)	768 (97.6%)	
Present	2 (7.4%)	10 (3.5%)	5 (2.4%)	2 (0.7%)	19 (2.4%)	
**Other fetal heart rate abnormalities**	**Valid *n* = 27**	**Valid *n* = 282**	**Valid *n* = 210**	**Valid *n* = 268**	**Valid *n* = 787**	**0.478†**
Absent	26 (96.3%)	281 (99.6%)	209 (99.5%)	268 (100.0%)	784 (99.6%)	
Variable fetal heart rate decelerations	0 (0.0%)	0 (0.0%)	1 (0.5%)	0 (0.0%)	1 (0.1%)	
Fetal tachycardia	0 (0.0%)	1 (0.4%)	0 (0.0%)	0 (0.0%)	1 (0.1%)	
Sustained fetal tachycardia	1 (3.7%)	0 (0.0%)	0 (0.0%)	0 (0.0%)	1 (0.1%)	
**Amniotic fluid abnormalities**	**Valid *n* = 27**	**Valid *n* = 282**	**Valid *n* = 210**	**Valid *n* = 268**	**Valid *n* = 787**	**0.330†**

Markers	Underweight *N* = 27	Normal weight *N* = 282	Overweight *N* = 210	Obesity *N* = 268	Total *N* = 787	*p*-Value
Absent	25 (92.6%)	279 (98.9%)	204 (97.1%)	263 (98.1%)	771 (98.0%)	
Present	2 (7.4%)	3 (1.1%)	6 (2.9%)	5 (1.9%)	16 (2.0%)	
**Amniotic fluid abnormalities: recorded type**	**Valid *n* = 27**	**Valid *n* = 282**	**Valid *n* = 210**	**Valid *n* = 268**	**Valid *n* = 787**	**0.202†**
Absent	25 (92.6%)	279 (98.9%)	204 (97.1%)	263 (98.1%)	771 (98.0%)	
Oligohydramnios	1 (3.7%)	2 (0.7%)	6 (2.9%)	3 (1.1%)	12 (1.5%)	
Polyhydramnios	1 (3.7%)	1 (0.4%)	0 (0.0%)	2 (0.7%)	4 (0.5%)	
**Polyhydramnios**	**Valid *n* = 27**	**Valid *n* = 282**	**Valid *n* = 210**	**Valid *n* = 268**	**Valid *n* = 787**	**0.637†**
Absent	26 (96.3%)	281 (99.6%)	210 (100.0%)	266 (99.3%)	783 (99.5%)	
Present	1 (3.7%)	1 (0.4%)	0 (0.0%)	2 (0.7%)	4 (0.5%)	
**Oligohydramnios**	**Valid *n* = 27**	**Valid *n* = 282**	**Valid *n* = 210**	**Valid *n* = 268**	**Valid *n* = 787**	**0.127†**
Absent	26 (96.3%)	280 (99.3%)	204 (97.1%)	265 (98.9%)	775 (98.5%)	
Present	1 (3.7%)	2 (0.7%)	6 (2.9%)	3 (1.1%)	12 (1.5%)	
**Rupture of membranes**	**Valid *n* = 27**	**Valid *n* = 282**	**Valid *n* = 210**	**Valid *n* = 268**	**Valid *n* = 787**	**0.904†**
Absent	26 (96.3%)	277 (98.2%)	205 (97.6%)	262 (97.8%)	770 (97.8%)	
Present	1 (3.7%)	5 (1.8%)	5 (2.4%)	6 (2.2%)	17 (2.2%)	
**Fetal congenital anomalies**	**Valid *n* = 27**	**Valid *n* = 282**	**Valid *n* = 210**	**Valid *n* = 268**	**Valid *n* = 787**	**0.547†**
Absent	26 (96.3%)	281 (99.6%)	208 (99.0%)	266 (99.3%)	781 (99.2%)	
Ventricular septal defect; congenital clubfoot	0 (0.0%)	0 (0.0%)	1 (0.5%)	0 (0.0%)	1 (0.1%)	
Fetal gastroschisis	1 (3.7%)	0 (0.0%)	0 (0.0%)	0 (0.0%)	1 (0.1%)	
Unspecified fetal malformations	0 (0.0%)	0 (0.0%)	1 (0.5%)	1 (0.4%)	2 (0.3%)	
Fetal omphalocele	0 (0.0%)	1 (0.4%)	0 (0.0%)	0 (0.0%)	1 (0.1%)	
Down syndrome	0 (0.0%)	0 (0.0%)	0 (0.0%)	1 (0.4%)	1 (0.1%)	
**Placental abnormalities**	**Valid *n* = 27**	**Valid *n* = 282**	**Valid *n* = 210**	**Valid *n* = 268**	**Valid *n* = 787**	**0.967†**
Absent	27 (100.0%)	280 (99.3%)	209 (99.5%)	266 (99.3%)	782 (99.4%)	
Placenta accreta	0 (0.0%)	1 (0.4%)	0 (0.0%)	0 (0.0%)	1 (0.1%)	
Placental abruption	0 (0.0%)	1 (0.4%)	1 (0.5%)	1 (0.4%)	3 (0.4%)	
Placenta previa	0 (0.0%)	0 (0.0%)	0 (0.0%)	1 (0.4%)	1 (0.1%)	
**Other obstetric, delivery, and puerperal conditions**	**Valid *n* = 27**	**Valid n = 282**	**Valid *n* = 210**	**Valid *n* = 268**	**Valid *n* = 787**	**0.291†**
Absent	27 (100.0%)	281 (99.6%)	206 (98.1%)	267 (99.6%)	781 (99.2%)	
Uterine atony	0 (0.0%)	0 (0.0%)	1 (0.5%)	0 (0.0%)	1 (0.1%)	
Labor progression dystocia	0 (0.0%)	0 (0.0%)	1 (0.5%)	0 (0.0%)	1 (0.1%)	
Hemorrhage	0 (0.0%)	0 (0.0%)	1 (0.5%)	0 (0.0%)	1 (0.1%)	
Uterine hypotonia	0 (0.0%)	0 (0.0%)	0 (0.0%)	1 (0.4%)	1 (0.1%)	
Cervical insufficiency	0 (0.0%)	1 (0.4%)	1 (0.5%)	0 (0.0%)	2 (0.3%)	
**Perinatal outcomes**						
**Gestational age at birth**	**Valid *n* = 27**	**Valid *n* = 277**	**Valid *n* = 210**	**Valid *n* = 266**	**Valid *n* = 780**	**0.892†**
Preterm (<37 weeks)	6 (22.2%)	44 (15.9%)	35 (16.7%)	45 (16.9%)	130 (16.7%)	
Term (37 weeks to 41 weeks and 6 days)	21 (77.8%)	232 (83.8%)	173 (82.4%)	220 (82.7%)	646 (82.8%)	
Post-term (≥42 completed weeks)	0 (0.0%)	1 (0.4%)	2 (1.0%)	1 (0.4%)	4 (0.5%)	
Missing data	0 (0.0%)	5 (1.8%)	0 (0.0%)	2 (0.7%)	7 (0.9%)	
**Birth weight classification**	**Valid *n* = 27**	**Valid *n* = 276**	**Valid *n* = 210**	**Valid *n* = 265**	**Valid *n* = 778**	**0.001†**
Extremely low birth weight (<1000 g)	1 (3.7%)	3 (1.1%)	1 (0.5%)	3 (1.1%)	8 (1.0%)	
Very low birth weight (1000–1499 g)	0 (0.0%)	4 (1.4%)	4 (1.9%)	3 (1.1%)	11 (1.4%)	
Low birth weight (1500–2499 g)	6 (22.2%)	32 (11.6%)	29 (13.8%)	21 (7.9%)	88 (11.3%)	
Normal birth weight 2500–3999 g	20 (74.1%)	233 (84.4%)	167 (79.5%)	212 (80.0%)	632 (81.2%)	
Macrosomia (≥4000 g)	0 (0.0%)	4 (1.4%)	9 (4.3%)	26 (9.8%)	39 (5.0%)	
Missing data	0 (0.0%)	6 (2.1%)	0 (0.0%)	3 (1.1%)	9 (1.1%)	
**Birth weight for gestational age**	**Valid *n* = 27**	**Valid *n* = 280**	**Valid *n* = 210**	**Valid *n* = 266**	**Valid *n* = 783**	**<0.001#**
Small for gestational age	3 (11.1%)	22 (7.9%)	11 (5.2%)	17 (6.4%)	53 (6.8%)	
Appropriate for gestational age	24 (88.9%)	250 (89.3%)	178 (84.8%)	208 (78.2%)	660 (84.3%)	
Large for gestational age	0 (0.0%)	8 (2.9%)	21 (10.0%)	41 (15.4%)	70 (8.9%)	
Missing data	0 (0.0%)	2 (0.7%)	0 (0.0%)	2 (0.7%)	4 (0.5%)	
**Apgar score at 5 minutes**	**Valid *n* = 27**	**Valid *n* = 279**	**Valid *n* = 209**	**Valid *n* = 266**	**Valid *n* = 781**	**0.103†**
0	0 (0.0%)	0 (0.0%)	2 (1.0%)	1 (0.4%)	3 (0.4%)	
3	0 (0.0%)	0 (0.0%)	0 (0.0%)	1 (0.4%)	1 (0.1%)	
4	0 (0.0%)	1 (0.4%)	0 (0.0%)	4 (1.5%)	5 (0.6%)	
5	0 (0.0%)	3 (1.1%)	0 (0.0%)	0 (0.0%)	3 (0.4%)	

Markers	Underweight $N = 27$	Normal weight $N = 282$	Overweight $N = 210$	Obesity $N = 268$	Total $N = 787$	*p*-Value
6	0 (0.0%)	4 (1.4%)	1 (0.5%)	2 (0.8%)	7 (0.9%)	
7	0 (0.0%)	19 (6.8%)	10 (4.8%)	19 (7.1%)	48 (6.1%)	
8	2 (7.4%)	23 (8.2%)	28 (13.4%)	19 (7.1%)	72 (9.2%)	
9	23 (85.2%)	209 (74.9%)	156 (74.6%)	201 (75.6%)	589 (75.4%)	
10	2 (7.4%)	17 (6.1%)	11 (5.3%)	15 (5.6%)	45 (5.8%)	
Not applicable	0 (0.0%)	3 (1.1%)	1 (0.5%)	4 (1.5%)	8 (1.0%)	
Missing data	0 (0.0%)	3 (1.1%)	1 (0.5%)	2 (0.7%)	6 (0.8%)	
**Apgar score category at 5 minutes**	**Valid *n* = 27**	**Valid *n* = 279**	**Valid *n* = 209**	**Valid *n* = 266**	**Valid *n* = 781**	**0.184†**
Low (0–3)	0 (0.0%)	0 (0.0%)	2 (1.0%)	2 (0.8%)	4 (0.5%)	
Moderately abnormal (4–6)	0 (0.0%)	8 (2.9%)	1 (0.5%)	6 (2.3%)	15 (1.9%)	
Reassuring (7–10)	27 (100.0%)	268 (96.1%)	205 (98.1%)	254 (95.5%)	754 (96. 5%)	
Not applicable	0 (0.0%)	3 (1.1%)	1 (0.5%)	4 (1.5%)	8 (1.0%)	
Missing data	0 (0.0%)	3 (1.1%)	1 (0.5%)	2 (0.7%)	6 (0.8%)	
**Composite adverse perinatal outcome**	**Valid *n* = 27**	**Valid *n* = 279**	**Valid *n* = 209**	**Valid *n* = 266**	**Valid *n* = 781**	**0.349#**
No	27 (100.0%)	266 (95.3%)	203 (97.1%)	251 (94.4%)	747 (95.6%)	
Yes	0 (0.0%)	13 (4.7%)	6 (2.9%)	15 (5.6%)	34 (4.4%)	
Missing data	0 (0.0%)	3 (1.1%)	1 (0.5%)	2 (0.7%)	6 (0.8%)	
**NICU admission or death**	**Valid *n* = 27**	**Valid *n* = 282**	**Valid *n* = 210**	**Valid *n* = 268**	**Valid *n* = 787**	**0.724†**
No	27 (100.0%)	276 (97.9%)	206 (98.1%)	260 (97.0%)	769 (97.7%)	
Yes	0 (0.0%)	6 (2.1%)	4 (1.9%)	8 (3.0%)	18 (2.3%)	
**NICU admission**	**Valid *n* = 27**	**Valid *n* = 282**	**Valid *n* = 210**	**Valid *n* = 268**	**Valid *n* = 787**	**0.923†**
No	27 (100.0%)	279 (98.9%)	207 (98.6%)	264 (98.5%)	777 (98.7%)	
Yes	0 (0.0%)	3 (1.1%)	3 (1.4%)	4 (1.5%)	10 (1.3%)	
**Death**	**Valid *n* = 27**	**Valid *n* = 282**	**Valid *n* = 210**	**Valid *n* = 268**	**Valid *n* = 787**	**0.546†**
No	27 (100.0%)	279 (98.9%)	209 (99.5%)	264 (98.5%)	779 (99.0%)	
Yes (fetal death)	0 (0.0%)	3 (1.1%)	1 (0.5%)	4 (1.5%)	8 (1.0%)	
**Previous pregnancy losses**	**Valid *n* = 27**	**Valid *n* = 282**	**Valid *n* = 210**	**Valid *n* = 268**	**Valid *n* = 787**	**0.146†**
No	27 (100.0%)	279 (98.9%)	208 (99.0%)	260 (97.0%)	774 (98.3%)	
1 pregnancy loss	0 (0.0%)	2 (0.7%)	1 (0.5%)	6 (2.2%)	9 (1.1%)	
2 pregnancy losses	0 (0.0%)	0 (0.0%)	0 (0.0%)	2 (0.7%)	2 (0.3%)	
3 pregnancy losses	0 (0.0%)	1 (0.4%)	1 (0.5%)	0 (0.0%)	2 (0.3%)	

BMI, body mass index; HELLP, hemolysis, elevated liver enzymes, and low platelet count; HIV, human immunodeficiency virus; NICU, neonatal intensive care unit; PhD, Doctor of Philosophy. *n*, category frequency or number of events, as specified; *N*, group size or analysis denominator. #, Pearson chi-square without continuity correction. †, Conditional Monte Carlo test with fixed margins and 9999 replicates. Missing values are excluded from categorical tests. Perineal *p*-Values use vaginal deliveries; Apgar *p*-Values exclude not-applicable scores. Structural not-applicability remains descriptive. BMI was not imputed.

Table S7. Full description and bivariate comparisons by recorded maternal body mass index (BMI) after multiple imputation. Values are *n* (%). Maternal BMI was never imputed. Descriptive columns retain all 787 singleton pregnancies with recorded BMI. *p*-Values compare only normal weight, overweight, and obesity (*N* = 760). Underweight was excluded from association analyses during revision because the subgroup was small and had no composite event. Percentages were calculated before rounding. Fifty completed datasets were analyzed separately. Displayed counts are the mean counts rounded by the largest-remainder method within each BMI group and marker, preserving its total. The total column sums displayed group counts. Percentages use unrounded means and can differ slightly from percentages computed from the displayed integers, including a nonzero percentage with a rounded count of zero. These counts are presentation summaries, not a single completed dataset. Estimates and tests use unrounded data and account for between-imputation variation.

**Markers**		**Underweight** *N* = 27	**Normal weight** *N* = 282	**Overweight** *N* = 210	**Obesity** *N* = 268	**Total** *N* = 787	***p*-Value**
**Sociodemographic characteristics and lifestyle**							
**Race**							**0.677‡**
	White	20 (73.3%)	195 (69.2%)	156 (74.4%)	187 (69.9%)	558 (71.0%)	
	Mixed race	6 (22.8%)	72 (25.7%)	46 (21.7%)	65 (24.1%)	189 (24.0%)	
	Black	1 (3.9%)	15 (5.1%)	8 (3.9%)	16 (6.1%)	40 (5.1%)	
**Maternal age (years)**							**<0.001†**
	15-19	8 (29.6%)	47 (16.7%)	14 (6.7%)	10 (3.7%)	79 (10.0%)	
	20-24	10 (37.0%)	77 (27.3%)	48 (22.9%)	55 (20.5%)	190 (24.1%)	
	25-29	3 (11.1%)	65 (23.0%)	51 (24.3%)	80 (29.9%)	199 (25.3%)	
	30-34	6 (22.2%)	45 (16.0%)	49 (23.3%)	60 (22.4%)	160 (20.3%)	
	35-39	0 (0.0%)	34 (12.1%)	38 (18.1%)	44 (16.4%)	116 (14.7%)	
	40-44	0 (0.0%)	14 (5.0%)	8 (3.8%)	18 (6.7%)	40 (5.1%)	
	45-49	0 (0.0%)	0 (0.0%)	2 (1.0%)	1 (0.4%)	3 (0.4%)	
**Marital status**							**0.271‡**
	Married	5 (18.5%)	95 (33.5%)	78 (37.4%)	113 (42.1%)	291 (36.9%)	
	Divorced	0 (0.0%)	5 (1.9%)	6 (2.9%)	7 (2.7%)	18 (2.4%)	
	Single	21 (77.8%)	165 (58.7%)	108 (51.5%)	135 (50.5%)	429 (54.6%)	
	Stable union	1 (3.7%)	17 (6.0%)	18 (8.3%)	13 (4.7%)	49 (6.1%)	
**Educational attainment**							**0.646‡**
	Illiterate	0 (0.2%)	0 (0.1%)	1 (0.7%)	1 (0.1%)	2 (0.3%)	
	Incomplete elementary school	1 (4.1%)	17 (5.9%)	19 (9.0%)	24 (9.1%)	61 (7.7%)	
	Completed elementary school	6 (21.9%)	40 (14.2%)	31 (14.6%)	52 (19.4%)	129 (16.3%)	
	Incomplete high school	3 (11.9%)	25 (8.9%)	21 (10.0%)	21 (7.9%)	70 (9.0%)	
	Completed high school	14 (52.7%)	156 (55.5%)	112 (53.2%)	132 (49.1%)	414 (52.6%)	
	Incomplete higher education	0 (0.4%)	12 (4.2%)	5 (2.3%)	11 (4.2%)	28 (3.6%)	
	Completed higher education	3 (8.7%)	32 (11.2%)	21 (10.2%)	26 (9.7%)	82 (10.4%)	
	Doctorate/PhD	0 (0.0%)	0 (0.0%)	0 (0.0%)	1 (0.4%)	1 (0.1%)	
**Smoking**							**0.426#**
	No	24 (88.9%)	258 (91.5%)	198 (94.3%)	251 (93.7%)	731 (92.9%)	
	Yes	3 (11.1%)	24 (8.5%)	12 (5.7%)	17 (6.3%)	56 (7.1%)	
**Alcohol consumption**							**0.135#**
	No	24 (88.9%)	270 (95.7%)	207 (98.6%)	262 (97.8%)	763 (97.0%)	
	Yes	3 (11.1%)	12 (4.3%)	3 (1.4%)	6 (2.2%)	24 (3.0%)	
**Illicit drug use**							**0.190†**
	No	25 (92.6%)	277 (98.2%)	209 (99.5%)	267 (99.6%)	778 (98.9%)	
	Yes	2 (7.4%)	5 (1.8%)	1 (0.5%)	1 (0.4%)	9 (1.1%)	
**Maternal clinical conditions (any)**							
**Maternal clinical conditions**							**<0.001#**
	Absent	24 (88.9%)	227 (80.5%)	151 (71.9%)	155 (57.8%)	557 (70.8%)	
	Present	3 (11.1%)	55 (19.5%)	59 (28.1%)	113 (42.2%)	230 (29.2%)	
**Chronic hypertension**							**<0.001#**
	Absent	27 (100.0%)	274 (97.2%)	200 (95.2%)	210 (78.4%)	711 (90.3%)	
	Present	0 (0.0%)	8 (2.8%)	10 (4.8%)	58 (21.6%)	76 (9.7%)	
**Hypothyroidism**							**0.059#**
	Absent	25 (92.6%)	271 (96.1%)	199 (94.8%)	245 (91.4%)	740 (94.0%)	
	Present	2 (7.4%)	11 (3.9%)	11 (5.2%)	23 (8.6%)	47 (6.0%)	
**Urinary tract infection**							**0.352#**
	Absent	26 (96.3%)	273 (96. %8)	198 (94.3%)	258 (96.3%)	755 (95.9%)	

Markers	Underweight *N* = 27	Normal weight *N* = 282	Overweight *N* = 210	Obesity *N* = 268	Total *N* = 787	*p*-Value
Present	1 (3.7%)	9 (3.2%)	12 (5.7%)	10 (3.7%)	32 (4.1%)	
**Asthma**						**0.554#**
Absent	27 (100.0%)	274 (97.2%)	201 (95.7%)	256 (95.5%)	758 (96.3%)	
Present	0 (0.0%)	8 (2.8%)	9 (4.3%)	12 (4.5%)	29 (3.7%)	
**Pre-existing diabetes mellitus**						**0.197†**
Absent	27 (100.0%)	280 (99.3%)	205 (97.6%)	261 (97.4%)	773 (98.2%)	
Present	0 (0.0%)	2 (0.7%)	5 (2.4%)	7 (2.6%)	14 (1.8%)	
**Type of pre-existing diabetes mellitus**						**0.559†**
Absent	27 (100.0%)	280 (99.3%)	205 (97.6%)	261 (97.4%)	773 (98.2%)	
Type 1 diabetes mellitus	0 (0.0%)	0 (0.0%)	1 (0.5%)	1 (0.4%)	2 (0.3%)	
Type 2 diabetes mellitus	0 (0.0%)	2 (0.7%)	4 (1.9%)	6 (2.2%)	12 (1.5%)	
**Syphilis**						**0.639†**
Absent	27 (100.0%)	278 (98.6%)	209 (99.5%)	266 (99.3%)	780 (99.1%)	
Present	0 (0.0%)	4 (1.4%)	1 (0.5%)	2 (0.7%)	7 (0.9%)	
**Depression**						**0.007†**
Absent	27 (100.0%)	282 (100.0%)	204 (97.1%)	266 (99.3%)	779 (99.0%)	
Present	0 (0.0%)	0 (0.0%)	6 (2.9%)	2 (0.7%)	8 (1.0%)	
**Noninfectious renal and urological conditions**						**0.061†**
Absent	27 (100.0%)	280 (99.3%)	207 (98.6%)	267 (99.6%)	781 (99.2%)	
Renal atrophy	0 (0.0%)	1 (0.4%)	0 (0.0%)	0 (0.0%)	1 (0.1%)	
Glomerulonephritis	0 (0.0%)	0 (0.0%)	1 (0.5%)	0 (0.0%)	1 (0.1%)	
Hydronephrosis	0 (0.0%)	0 (0.0%)	0 (0.0%)	1 (0.4%)	1 (0.1%)	
Nephrolithiasis	0 (0.0%)	0 (0.0%)	2 (1.0%)	0 (0.0%)	2 (0.3%)	
Ureterocele; hydronephrosis	0 (0.0%)	1 (0.4%)	0 (0.0%)	0 (0.0%)	1 (0.1%)	
**Other cardiovascular conditions**						**0.473†**
Absent	27 (100.0%)	279 (98.9%)	209 (99.5%)	266 (99.3%)	781 (99.2%)	
Arrhythmia	0 (0.0%)	1 (0.4%)	0 (0.0%)	0 (0.0%)	1 (0.1%)	
Hypertrophic cardiomyopathy	0 (0.0%)	0 (0.0%)	0 (0.0%)	1 (0.4%)	1 (0.1%)	
Unspecified heart disease	0 (0.0%)	2 (0.7%)	0 (0.0%)	0 (0.0%)	2 (0.3%)	
Mitral valve prolapse	0 (0.0%)	0 (0.0%)	1 (0.5%)	1 (0.4%)	2 (0.3%)	
**Hematological conditions and thrombophilias**						**0.751†**
Absent	26 (96.3%)	278 (98.6%)	207 (98.6%)	267 (99.6%)	778 (98.9%)	
Anemia	1 (3.7%)	2 (0.7%)	2 (1.0%)	1 (0.4%)	6 (0.8%)	
Spherocytosis	0 (0.0%)	1 (0.4%)	0 (0.0%)	0 (0.0%)	1 (0.1%)	
Sickle cell trait	0 (0.0%)	1 (0.4%)	0 (0.0%)	0 (0.0%)	1 (0.1%)	
Hereditary thrombophilia associated with factor V Leiden	0 (0.0%)	0 (0.0%)	1 (0.5%)	0 (0.0%)	1 (0.1%)	
**Autoimmune conditions**						**0.102†**
Absent	27 (100.0%)	282 (100.0%)	208 (99.0%)	267 (99.6%)	784 (99.6%)	
Rheumatoid arthritis	0 (0.0%)	0 (0.0%)	0 (0.0%)	1 (0.4%)	1 (0.1%)	
Unspecified autoimmune skin disease	0 (0.0%)	0 (0.0%)	1 (0.5%)	0 (0.0%)	1 (0.1%)	
Systemic lupus erythematosus	0 (0.0%)	0 (0.0%)	1 (0.5%)	0 (0.0%)	1 (0.1%)	
**Gastrointestinal and hepatobiliary conditions and hernias**						**0.706†**
Absent	27 (100.0%)	280 (99.3%)	209 (99.5%)	264 (98.5%)	780 (99.1%)	
Cholecystitis	0 (0.0%)	0 (0.0%)	0 (0.0%)	1 (0.4%)	1 (0.1%)	
Cholelithiasis; pancreatitis	0 (0.0%)	0 (0.0%)	0 (0.0%)	1 (0.4%)	1 (0.1%)	
Crohn disease	0 (0.0%)	1 (0.4%)	0 (0.0%)	0 (0.0%)	1 (0.1%)	
Gastroesophageal reflux disease	0 (0.0%)	1 (0.4%)	0 (0.0%)	0 (0.0%)	1 (0.1%)	
Gastritis	0 (0.0%)	0 (0.0%)	1 (0.5%)	0 (0.0%)	1 (0.1%)	
Hiatal hernia	0 (0.0%)	0 (0.0%)	0 (0.0%)	1 (0.4%)	1 (0.1%)	
Umbilical hernia	0 (0.0%)	0 (0.0%)	0 (0.0%)	1 (0.4%)	1 (0.1%)	
**Neurological conditions and other mental disorders**						**0.387†**
Absent	27 (100.0%)	281 (99.6%)	207 (98.6%)	265 (98.9%)	780 (99.1%)	
Anxiety	0 (0.0%)	0 (0.0%)	0 (0.0%)	1 (0.4%)	1 (0.1%)	
Epilepsy	0 (0.0%)	1 (0.4%)	0 (0.0%)	1 (0.4%)	2 (0.3%)	
Epilepsy; syncope	0 (0.0%)	0 (0.0%)	1 (0.5%)	0 (0.0%)	1 (0.1%)	

Markers	Underweight N = 27	Normal weight N = 282	Overweight N = 210	Obesity N = 268	Total N = 787	p-Value
Labyrinthitis	0 (0.0%)	0 (0.0%)	0 (0.0%)	1 (0.4%)	1 (0.1%)	
Bipolar disorder	0 (0.0%)	0 (0.0%)	1 (0.5%)	0 (0.0%)	1 (0.1%)	
Panic disorder	0 (0.0%)	0 (0.0%)	1 (0.5%)	0 (0.0%)	1 (0.1%)	
**Gynecological conditions, neoplasms, and masses of undetermined nature**						**0.835†**
Absent	27 (100.0%)	279 (98.9%)	209 (99.5%)	265 (98.9%)	780 (99.1%)	
Ovarian cyst	0 (0.0%)	1 (0.4%)	0 (0.0%)	0 (0.0%)	1 (0.1%)	
Uterine leiomyomatosis	0 (0.0%)	1 (0.4%)	0 (0.0%)	1 (0.4%)	2 (0.3%)	
Cervical intraepithelial neoplasia	0 (0.0%)	1 (0.4%)	0 (0.0%)	0 (0.0%)	1 (0.1%)	
Ovarian tumor of unspecified nature	0 (0.0%)	0 (0.0%)	0 (0.0%)	1 (0.4%)	1 (0.1%)	
Mass in the bladder region	0 (0.0%)	0 (0.0%)	0 (0.0%)	1 (0.4%)	1 (0.1%)	
Bicornuate uterus	0 (0.0%)	0 (0.0%)	1 (0.5%)	0 (0.0%)	1 (0.1%)	
**Other maternal infections**						**0.277†**
Absent	27 (100.0%)	280 (99.3%)	205 (97.6%)	264 (98.5%)	776 (98.6%)	
Perianal condyloma acuminatum	0 (0.0%)	0 (0.0%)	1 (0.5%)	0 (0.0%)	1 (0.1%)	
Hepatitis B	0 (0.0%)	1 (0.4%)	0 (0.0%)	0 (0.0%)	1 (0.1%)	
Hepatitis C	0 (0.0%)	1 (0.4%)	0 (0.0%)	0 (0.0%)	1 (0.1%)	
HIV infection	0 (0.0%)	0 (0.0%)	2 (1.0%)	1 (0.4%)	3 (0.4%)	
Pneumonia	0 (0.0%)	0 (0.0%)	0 (0.0%)	1 (0.4%)	1 (0.1%)	
Toxoplasmosis	0 (0.0%)	0 (0.0%)	2 (1.0%)	2 (0.7%)	4 (0.5%)	
**Other specified clinical conditions and medical history**						**0.777†**
Absent	27 (100.0%)	279 (98.9%)	208 (99.0%)	265 (98.9%)	779 (99.0%)	
Previous surgery	0 (0.0%)	0 (0.0%)	1 (0.5%)	2 (0.7%)	3 (0.4%)	
Fibromyalgia	0 (0.0%)	0 (0.0%)	1 (0.5%)	1 (0.4%)	2 (0.3%)	
Emotional lability	0 (0.0%)	1 (0.4%)	0 (0.0%)	0 (0.0%)	1 (0.1%)	
Rickets; scoliosis	0 (0.0%)	1 (0.4%)	0 (0.0%)	0 (0.0%)	1 (0.1%)	
Allergic rhinitis	0 (0.0%)	1 (0.4%)	0 (0.0%)	0 (0.0%)	1 (0.1%)	
**Delivery and reproductive history**						
**Mode of delivery**						**<0.001‡**
Cesarean delivery	19 (70.4%)	170 (60.4%)	170 (81.0%)	221 (82.5%)	580 (73.8%)	
Vaginal delivery	8 (29.6%)	112 (39.6%)	40 (19.0%)	47 (17.5%)	207 (26.2%)	
**Episiotomy**						**0.185‡**
No	6 (22.2%)	67 (23.9%)	19 (9.0%)	31 (11.7%)	123 (15.7%)	
Yes	2 (7.4%)	44 (15.7%)	21 (10.0%)	16 (5.8%)	83 (10.5%)	
Not applicable	19 (70.4%)	171 (60.4%)	170 (81.0%)	221 (82.5%)	581 (73.8%)	
**Recorded duration of surgery or labor (minutes)**						**0.002‡**
<30	2 (7.4%)	25 (8.7%)	8 (3.8%)	13 (5.0%)	48 (6.1%)	
30–60	21 (77.8%)	163 (58.0%)	115 (54.7%)	135 (50.3%)	434 (55.2%)	
60–120	4 (14.8%)	91 (32.2%)	84 (40.0%)	105 (39.0%)	284 (36.0%)	
>120	0 (0.0%)	3 (1.1%)	3 (1.4%)	15 (5.7%)	21 (2.7%)	
**Perineal tear at vaginal delivery**						**0.183‡**
No tear	6 (22.2%)	84 (29.9%)	33 (15.6%)	28 (10.4%)	151 (19.2%)	
First degree	1 (3.7%)	15 (5.4%)	3 (1.5%)	11 (4.0%)	30 (3.8%)	
Second degree	1 (3.7%)	12 (4.3%)	4 (1.9%)	8 (3.1%)	25 (3.2%)	
Not applicable	19 (70.4%)	171 (60.4%)	170 (81.0%)	221 (82.5%)	581 (73.8%)	
**Gravidity**						**<0.001‡**
Primigravida (1 pregnancy)	19 (70.4%)	148 (52.4%)	71 (33.8%)	95 (35.6%)	333 (42.4%)	
Secundigravida (2 pregnancies)	2 (7.4%)	58 (20.7%)	48 (23.0%)	60 (22.3%)	168 (21.4%)	
Multigravida (≥3 pregnancies)	6 (22.2%)	76 (26.8%)	91 (43.2%)	113 (42.0%)	286 (36.2%)	
**Parity**						**<0.001‡**
1 delivery	19 (70.4%)	158 (56.0%)	78 (37.2%)	102 (38.2%)	357 (45.4%)	
2 deliveries	6 (22.2%)	55 (19.5%)	50 (23.8%)	62 (23.0%)	173 (21.9%)	
≥3 deliveries	2 (7.4%)	69 (24.6%)	82 (39.0%)	104 (38.8%)	257 (32.7%)	
**Obstetric and fetal conditions**						
**Obstetric complications (any)**						**<0.001#**
Absent	20 (74.1%)	218 (77.3%)	129 (61.4%)	121 (45.1%)	488 (62.0%)	

Markers	Underweight<br>*N* = 27	Normal weight<br>*N* = 282	Overweight<br>*N* = 210	Obesity<br>*N* = 268	Total<br>*N* = 787	*p*-Value
Present	7 (25.9%)	64 (22.7%)	81 (38.6%)	147 (54.9%)	299 (38.0%)	
**Gestational diabetes mellitus**						**<0.001#**
Absent	26 (96.3%)	254 (90.1%)	173 (82.4%)	209 (78.0%)	662 (84.1%)	
Present	1 (3.7%)	28 (9.9%)	37 (17.6%)	59 (22.0%)	125 (15.9%)	
**Hypertensive disorders of pregnancy**						**<0.001#**
Absent	26 (96.3%)	262 (92.9%)	185 (88.1%)	184 (68.7%)	657 (83.5%)	
Present	1 (3.7%)	20 (7.1%)	25 (11.9%)	84 (31.3%)	130 (16.5%)	
**Hypertensive disorders of pregnancy: recorded type**						**<0.001†**
Absent	26 (96.3%)	262 (92.9%)	185 (88.1%)	184 (68.7%)	657 (83.5%)	
Hypertensive disorder of pregnancy	0 (0.0%)	18 (6.4%)	24 (11.4%)	82 (30.6%)	124 (15.8%)	
Hypertensive disorder of pregnancy; preeclampsia	0 (0.0%)	0 (0.0%)	0 (0.0%)	1 (0.4%)	1 (0.1%)	
Hypertensive disorder of pregnancy; HELLP syndrome	0 (0.0%)	1 (0.4%)	0 (0.0%)	1 (0.4%)	2 (0.3%)	
Eclampsia	1 (3.7%)	0 (0.0%)	0 (0.0%)	0 (0.0%)	1 (0.1%)	
Preeclampsia	0 (0.0%)	1 (0.4%)	1 (0.5%)	0 (0.0%)	2 (0.3%)	
**Fetal growth abnormalities**						**0.047†**
Absent	26 (96.3%)	282 (100.0%)	207 (98.6%)	262 (97.8%)	777 (98.7%)	
Present	1 (3.7%)	0 (0.0%)	3 (1.4%)	6 (2.2%)	10 (1.3%)	
**Fetal growth abnormalities: recorded type**						**0.031†**
Absent	26 (96.3%)	282 (100.0%)	207 (98.6%)	262 (97.8%)	777 (98.7%)	
Fetal macrosomia	0 (0.0%)	0 (0.0%)	0 (0.0%)	3 (1.1%)	3 (0.4%)	
Intrauterine growth restriction	1 (3.7%)	0 (0.0%)	3 (1.4%)	3 (1.1) %	7 (0.9%)	
**Intrauterine growth restriction**						**0.138†**
Absent	26 (96.3%)	282 (100.0%)	207 (98.6%)	265 (98.9%)	780 (99.1%)	
Present	1 (3.7%)	0 (0.0%)	3 (1.4%)	3 (1.1%)	7 (0.9%)	
**Fetal macrosomia**						**0.066†**
Absent	27 (100.0%)	282 (100.0%)	210 (100.0%)	265 (98.9%)	784 (99.6%)	
Present	0 (0.0%)	0 (0.0%)	0 (0.0%)	3 (1.1%)	3 (0.4%)	
**Fetal status abnormalities**						**0.056#**
Absent	25 (92.6%)	271 (96.1%)	204 (97.1%)	266 (99.3%)	766 (97.3%)	
Present	2 (7.4%)	11 (3.9%)	6 (2.9%)	2 (0.7%)	21 (2.7%)	
**Fetal status abnormalities: recorded type**						**0.071†**
Absent	25 (92.6%)	271 (96.1%)	204 (97.1%)	266 (99.3%)	766 (97.3%)	
Variable fetal heart rate decelerations	0 (0.0%)	0 (0.0%)	1 (0.5%)	0 (0.0%)	1 (0.1%)	
Fetal distress	1 (3.7%)	10 (3.5%)	5 (2.4%)	2 (0.7%)	18 (2.3%)	
Fetal distress; sustained fetal tachycardia	1 (3.7%)	0 (0.0%)	0 (0.0%)	0 (0.0%)	1 (0.1%)	
Fetal tachycardia	0 (0.0%)	1 (0.4%)	0 (0.0%)	0 (0.0%)	1 (0.1%)	
**Fetal distress**						**0.080†**
Absent	25 (92.6%)	272 (96.5%)	205 (97.6%)	266 (99.3%)	768 (97.6%)	
Present	2 (7.4%)	10 (3.5%)	5 (2.4%)	2 (0.7%)	19 (2.4%)	
**Other fetal heart rate abnormalities**						**0.478†**
Absent	26 (96.3%)	281 (99.6%)	209 (99.5%)	268 (100.0%)	784 (99.6%)	
Variable fetal heart rate decelerations	0 (0.0%)	0 (0.0%)	1 (0.5%)	0 (0.0%)	1 (0.1%)	
Fetal tachycardia	0 (0.0%)	1 (0.4%)	0 (0.0%)	0 (0.0%)	1 (0.1%)	
Sustained fetal tachycardia	1 (3.7%)	0 (0.0%)	0 (0.0%)	0 (0.0%)	1 (0.1%)	
**Amniotic fluid abnormalities**						**0.330†**
Absent	25 (92.6%)	279 (98.9%)	204 (97.1%)	263 (98.1%)	771 (98.0%)	
Present	2 (7.4%)	3 (1.1%)	6 (2.9%)	5 (1.9%)	16 (2.0%)	
**Amniotic fluid abnormalities: recorded type**						**0.202†**
Absent	25 (92.6%)	279 (98.9%)	204 (97.1%)	263 (98.1%)	771 (98.0%)	
Oligohydramnios	1 (3.7%)	2 (0.7%)	6 (2.9%)	3 (1.1%)	12 (1.5%)	
Polyhydramnios	1 (3.7%)	1 (0.4%)	0 (0.0%)	2 (0.7%)	4 (0.5%)	
**Polyhydramnios**						**0.637†**
Absent	26 (96.3%)	281 (99.6%)	210 (100.0%)	266 (99.3%)	783 (99.5%)	
Present	1 (3.7%)	1 (0.4%)	0 (0.0%)	2 (0.7%)	4 (0.5%)	
**Oligohydramnios**						**0.127†**

Markers	Underweight *N* = 27	Normal weight *N* = 282	Overweight *N* = 210	Obesity *N* = 268	Total *N* = 787	*p*-Value
Absent	26 (96.3%)	280 (99.3%)	204 (97.1%)	265 (98.9%)	775 (98.5%)	
Present	1 (3.7%)	2 (0.7%)	6 (2.9%)	3 (1.1%)	12 (1.5%)	
**Rupture of membranes**						**0.904†**
Absent	26 (96.3%)	277 (98.2%)	205 (97.6%)	262 (97.8%)	770 (97.8%)	
Present	1 (3.7%)	5 (1.8%)	5 (2.4%)	6 (2.2%)	17 (2.2%)	
**Fetal congenital anomalies**						**0.547†**
Absent	26 (96.3%)	281 (99.6%)	208 (99.0%)	266 (99.3%)	781 (99.2%)	
Ventricular septal defect; congenital clubfoot	0 (0.0%)	0 (0.0%)	1 (0.5%)	0 (0.0%)	1 (0.1%)	
Fetal gastroschisis	1 (3.7%)	0 (0.0%)	0 (0.0%)	0 (0.0%)	1 (0.1%)	
Unspecified fetal malformations	0 (0.0%)	0 (0.0%)	1 (0.5%)	1 (0.4%)	2 (0.3%)	
Fetal omphalocele	0 (0.0%)	1 (0.4%)	0 (0.0%)	0 (0.0%)	1 (0.1%)	
Down syndrome	0 (0.0%)	0 (0.0%)	0 (0.0%)	1 (0.4%)	1 (0.1%)	
**Placental abnormalities**						**0.967†**
Absent	27 (100.0%)	280 (99.3%)	209 (99.5%)	266 (99.3%)	782 (99.4%)	
Placenta accreta	0 (0.0%)	1 (0.4%)	0 (0.0%)	0 (0.0%)	1 (0.1%)	
Placental abruption	0 (0.0%)	1 (0.4%)	1 (0.5%)	1 (0.4%)	3 (0.4%)	
Placenta previa	0 (0.0%)	0 (0.0%)	0 (0.0%)	1 (0.4%)	1 (0.1%)	
**Other obstetric, delivery, and puerperal conditions**						**0.291†**
Absent	27 (100.0%)	281 (99.6%)	206 (98.1%)	267 (99.6%)	781 (99.2%)	
Uterine atony	0 (0.0%)	0 (0.0%)	1 (0.5%)	0 (0.0%)	1 (0.1%)	
Labor progression dystocia	0 (0.0%)	0 (0.0%)	1 (0.5%)	0 (0.0%)	1 (0.1%)	
Hemorrhage	0 (0.0%)	0 (0.0%)	1 (0.5%)	0 (0.0%)	1 (0.1%)	
Uterine hypotonia	0 (0.0%)	0 (0.0%)	0 (0.0%)	1 (0.4%)	1 (0.1%)	
Cervical insufficiency	0 (0.0%)	1 (0.4%)	1 (0.5%)	0 (0.0%)	2 (0.3%)	
**Perinatal outcomes**						
**Gestational age at birth**						**0.905‡**
Preterm (<37 weeks)	6 (22.2%)	46 (16.3%)	35 (16.7%)	45 (16.9%)	132 (16.8%)	
Term (37 weeks to 41 weeks and 6 days)	21 (77.8%)	235 (83.3%)	173 (82.4%)	222 (82.7%)	651 (82.7%)	
Post-term (≥42 completed weeks)	0 (0.0%)	1 (0.4%)	2 (1.0%)	1 (0.4%)	4 (0.5%)	
**Birth weight classification**						**0.002‡**
Extremely low birth weight (<1000 g)	1 (3.7%)	4 (1.3%)	1 (0.5%)	3 (1.3%)	9 (1.1%)	
Very low birth weight (1000–1499 g)	0 (0.0%)	4 (1.5%)	4 (1.9%)	3 (1.3%)	11 (1.5%)	
Low birth weight (1500–2499 g)	6 (22.2%)	33 (11.6%)	29 (13.8%)	22 (8.0%)	90 (11.3%)	
Normal birth weight (2500–3999 g)	20 (74.1%)	237 (84.2%)	167 (79.5%)	214 (79.7%)	638 (81.1%)	
Macrosomia (≥4000 g)	0 (0.0%)	4 (1.5%)	9 (4.3%)	26 (9.7%)	39 (5.0%)	
**Birth weight for gestational age**						**<0.001‡**
Small for gestational age	3 (11.1%)	23 (8.1%)	11 (5.2%)	18 (6.6%)	55 (6.9%)	
Appropriate for gestational age	24 (88.9%)	251 (89.0%)	178 (84.8%)	209 (78.1%)	662 (84.1%)	
Large for gestational age	0 (0.0%)	8 (2.9%)	21 (10.0%)	41 (15.3%)	70 (8.9%)	
**Apgar score at 5 minutes**						**—§**
0	0 (0.0%)	0 (0.0%)	2 (1.0%)	1 (0.4%)	3 (0.4%)	
3	0 (0.0%)	0 (0.0%)	0 (0.0%)	1 (0.4%)	1 (0.1%)	
4	0 (0.0%)	1 (0.4%)	0 (0.0%)	4 (1.5%)	5 (0.6%)	
5	0 (0.0%)	3 (1.1%)	0 (0.0%)	0 (0.0%)	3 (0.4%)	
6	0 (0.0%)	4 (1.4%)	1 (0.5%)	2 (0.8%)	7 (0.9%)	
7	0 (0.0%)	19 (6.8%)	10 (4.8%)	19 (7.1%)	48 (6.2%)	
8	2 (7.4%)	24 (8.2%)	28 (13.4%)	19 (7.2%)	73 (9.2%)	
9	23 (85.2%)	211 (74.9%)	157 (74.6%)	203 (75.6%)	594 (75.4%)	
10	2 (7.4%)	17 (6.1%)	11 (5.2%)	15 (5.6%)	45 (5.8%)	
Not applicable	0 (0.0%)	3 (1.1%)	1 (0.5%)	4 (1.5%)	8 (1.0%)	
**Apgar score category at 5 minutes**						**0.195‡**
Low (0–3)	0 (0.0%)	0 (0.0%)	2 (1.0%)	2 (0.7%)	4 (0.5%)	
Moderately abnormal (4–6)	0 (0.0%)	8 (2.9%)	1 (0.5%)	6 (2.3%)	15 (1.9%)	
Reassuring (7–10)	27 (100.0%)	271 (96.0%)	206 (98.1%)	256 (95.5%)	760 (96.5%)	
Not applicable	0 (0.0%)	3 (1.1%)	1 (0.5%)	4 (1.5%)	8 (1.0%)	
**Composite adverse perinatal outcome**						**0.354‡**
No	27 (100.0%)	269 (95.3%)	204 (97.1%)	253 (94.4%)	753 (95.6%)	

Markers		Underweight<br>*N* = 27	Normal weight<br>*N* = 282	Overweight<br>*N* = 210	Obesity<br>*N* = 268	Total<br>*N* = 787	*p*-Value
	Yes	0 (0.0%)	13 (4.7%)	6 (2.9%)	15 (5.6%)	34 (4.4%)	
**NICU admission or death**							**0.724†**
	No	27 (100.0%)	276 (97.9%)	206 (98.1%)	260 (97.0%)	769 (97.7%)	
	Yes	0 (0.0%)	6 (2.1%)	4 (1.9%)	8 (3.0%)	18 (2.3%)	
**NICU admission**							**0.923†**
	No	27 (100.0%)	279 (98.9%)	207 (98.6%)	264 (98.5%)	777 (98.7%)	
	Yes	0 (0.0%)	3 (1.1%)	3 (1.4%)	4 (1.5%)	10 (1.3%)	
**Death**							**0.546†**
	No	27 (100.0%)	279 (98.9%)	209 (99.5%)	264 (98.5%)	779 (99.0%)	
	Yes (fetal death)	0 (0.0%)	3 (1.1%)	1 (0.5%)	4 (1.5%)	8 (1.0%)	
**Previous pregnancy losses**							**0.146†**
	No	27 (100.0%)	279 (98.9%)	208 (99.0%)	260 (97.0%)	774 (98.3%)	
	1 pregnancy loss	0 (0.0%)	2 (0.7%)	1 (0.5%)	6 (2.2%)	9 (1.1%)	
	2 pregnancy losses	0 (0.0%)	0 (0.0%)	0 (0.0%)	2 (0.7%)	2 (0.3%)	
	3 pregnancy losses	0 (0.0%)	1 (0.4%)	1 (0.5%)	0 (0.0%)	2 (0.3%)	

BMI, body mass index; HELLP, hemolysis, elevated liver enzymes, and low platelet count; HIV, human immunodeficiency virus; NICU, neonatal intensive care unit; PhD, Doctor of Philosophy. n, category frequency or number of events, as specified; *N*, group size or analysis denominator. Rounding was performed separately within the groups displayed in each table. Consequently, aggregated rounded counts may differ by one participant between tables that use different grouping structures; percentages and statistical tests are based on unrounded data. #, Pearson chi-square without continuity correction. †, Conditional Monte Carlo test with fixed margins and 9999 replicates. ‡, D2 pooling of Pearson chi-square statistics across 50 imputations. D2 results with sparse cells are approximate. §, The global comparison of numerical Apgar scores was not pooled because category support differed across imputations. Numerical scores are reported descriptively; the grouped Apgar comparison is presented separately. —, Not estimated or not pooled, as specified in the accompanying note. Unchanged markers retain their original test and *p*-Value. Perineal global *p*-Values are restricted to vaginal deliveries. Not-applicable perineal and Apgar values are retained in the full descriptive counts, but not-applicable Apgar values are excluded from its test. No global *p*-Value was pooled for the numerical Apgar score because an extremely rare score appeared in only some completed datasets; the clinically grouped score was analyzed instead.

Table S8. Comparison of participants with and without recorded maternal body mass index (BMI) before multiple imputation among 999 singleton pregnancies.

Values are *n* (%). The valid sample size for each marker is shown in its heading row. Percentages for observed categories use that valid sample size; percentages for missing data use the full group size. All maternal BMI categories, including underweight, are retained in the group with recorded BMI.

Markers	BMI recorded (*N* = 787)	BMI missing (*N* = 212)	Total (*N* = 999)	*p*-Value
**Sociodemographic characteristics and lifestyle**				
**Race**	**Valid *n* = 773**	**Valid *n* = 206**	**Valid *n* = 979**	**0.124#**
White	548 (70.9%)	134 (65.0%)	682 (69.7%)	
Mixed race (*Pardo*)	186 (24.1%)	55 (26.7%)	241 (24.6%)	
Black	39 (5.0%)	17 (8.3%)	56 (5.7%)	
Missing data	14 (1.8%)	6 (2.8%)	20 (2.0%)	
**Maternal age (years)**	**Valid *n* = 787**	**Valid *n* = 212**	**Valid *n* = 999**	**0.297†**
15-19	79 (10.0%)	31 (14.6%)	110 (11.0%)	
20-24	190 (24.1%)	59 (27.8%)	249 (24.9%)	
25-29	199 (25.3%)	51 (24.1%)	250 (25.0%)	
30-34	160 (20.3%)	38 (17.9%)	198 (19.8%)	
35-39	116 (14.7%)	25 (11.8%)	141 (14.1%)	
40-44	40 (5.1%)	8 (3.8%)	48 (4.8%)	
45-49	3 (0.4%)	0 (0.0%)	3 (0.3%)	
**Marital status**	**Valid *n* = 760**	**Valid *n* = 202**	**Valid *n* = 962**	**0.892†**
Married	281 (37.0%)	69 (34.2%)	350 (36.4%)	
Divorced	18 (2.4%)	5 (2.5%)	23 (2.4%)	
Single	415 (54.6%)	116 (57.4%)	531 (55.2%)	
Stable union	46 (6.1%)	12 (5.9%)	58 (6.0%)	
Missing data	27 (3.4%)	10 (4.7%)	37 (3.7%)	
**Educational attainment**	**Valid *n* = 705**	**Valid *n* = 182**	**Valid *n* = 887**	**0.812†**
Illiterate	1 (0.1%)	1 (0.5%)	2 (0.2%)	
Incomplete elementary school	54 (7.7%)	13 (7.1%)	67 (7.6%)	
Completed elementary school	115 (16.3%)	26 (14.3%)	141 (15.9%)	
Incomplete high school	63 (8.9%)	16 (8.8%)	79 (8.9%)	
Completed high school	373 (52.9%)	106 (58.2%)	479 (54.0%)	
Incomplete higher education	25 (3.5%)	4 (2.2%)	29 (3.3%)	
Completed higher education	73 (10.4%)	16 (8.8%)	89 (10.0%)	
Doctorate/PhD	1 (0.1%)	0 (0.0%)	1 (0.1%)	
Missing data	82 (10.4%)	30 (14.2%)	112 (11.2%)	
**Smoking**	**Valid *n* = 787**	**Valid *n* = 212**	**Valid *n* = 999**	**0.880***
No	731 (92.9%)	198 (93.4%)	929 (93.0%)	
Yes	56 (7.1%)	14 (6.6%)	70 (7.0%)	
**Alcohol consumption**	**Valid *n* = 787**	**Valid *n* = 212**	**Valid *n* = 999**	**0.239***
No	763 (97.0%)	209 (98.6%)	972 (97.3%)	
Yes	24 (3.0%)	3 (1.4%)	27 (2.7%)	
**Illicit drug use**	**Valid *n* = 787**	**Valid *n* = 212**	**Valid *n* = 999**	**0.698***
No	778 (98.9%)	211 (99.5%)	989 (99.0%)	
Yes	9 (1.1%)	1 (0.5%)	10 (1.0%)	
**Maternal clinical conditions**				
**Maternal clinical conditions (any)**	**Valid *n* = 787**	**Valid *n* = 212**	**Valid *n* = 999**	**0.015***
Absent	557 (70.8%)	168 (79.2%)	725 (72.6%)	
Present	230 (29.2%)	44 (20.8%)	274 (27.4%)	
**Chronic hypertension**	**Valid *n* = 787**	**Valid *n* = 212**	**Valid *n* = 999**	**0.040***
Absent	711 (90.3%)	201 (94.8%)	912 (91.3%)	
Present	76 (9.7%)	11 (5.2%)	87 (8.7%)	
**Hypothyroidism**	**Valid *n* = 787**	**Valid *n* = 212**	**Valid *n* = 999**	**0.743***
Absent	740 (94.0%)	201 (94.8%)	941 (94.2%)	
Present	47 (6.0%)	11 (5.2%)	58 (5.8%)	
**Urinary tract infection**	**Valid *n* = 787**	**Valid n = 212**	**Valid *n* = 999**	**0.345***
Absent	755 (95.9%)	200 (94.3%)	955 (95.6%)	
Present	32 (4.1%)	12 (5.7%)	44 (4.4%)	

Markers	BMI recorded (*N* = 787)	BMI missing (*N* = 212)	Total (*N* = 999)	*p*-Value
**Asthma**	**Valid *n* = 787**	**Valid *n* = 212**	**Valid *n* = 999**	**0.123***
Absent	758 (96.3%)	209 (98.6%)	967 (96.8%)	
Present	29 (3.7%)	3 (1.4%)	32 (3.2%)	
**Pre-existing diabetes mellitus**	**Valid *n* = 787**	**Valid n = 212**	**Valid *n* = 999**	**0.574***
Absent	773 (98.2%)	207 (97.6%)	980 (98.1%)	
Present	14 (1.8%)	5 (2.4%)	19 (1.9%)	
**Type of pre-existing diabetes mellitus**	**Valid *n* = 787**	**Valid *n* = 212**	**Valid *n* = 999**	**0.901†**
Absent	773 (98.2%)	207 (97.6%)	980 (98.1%)	
Type 1 diabetes mellitus	2 (0.3%)	1 (0.5%)	3 (0.3%)	
Type 2 diabetes mellitus	12 (1.5%)	4 (1.9%)	16 (1.6%)	
**Syphilis**	**Valid *n* = 787**	**Valid *n* = 212**	**Valid *n* = 999**	**0.450***
Absent	780 (99.1%)	209 (98.6%)	989 (99.0%)	
Present	7 (0.9%)	3 (1.4%)	10 (1.0%)	
**Depression**	**Valid *n* = 787**	**Valid *n* = 212**	**Valid *n* = 999**	**1.000***
Absent	779 (99.0%)	210 (99.1%)	989 (99.0%)	
Present	8 (1.0%)	2 (0.9%)	10 (1.0%)	
**Noninfectious renal and urological conditions**	**Valid *n* = 787**	**Valid *n* = 212**	**Valid *n* = 999**	**0.401†**
Absent	781 (99.2%)	210 (99.1%)	991 (99.2%)	
Renal atrophy	1 (0.1%)	0 (0.0%)	1 (0.1%)	
Ureteropelvic junction stenosis	0 (0.0%)	1 (0.5%)	1 (0.1%)	
Glomerulonephritis	1 (0.1%)	0 (0.0%)	1 (0.1%)	
Hydronephrosis	1 (0.1%)	0 (0.0%)	1 (0.1%)	
Nephrolithiasis	2 (0.3%)	0 (0.0%)	2 (0.2%)	
Unspecified nephropathy	0 (0.0%)	1 (0.5%)	1 (0.1%)	
Ureterocele; hydronephrosis	1 (0.1%)	0 (0.0%)	1 (0.1%)	
**Other cardiovascular conditions**	**Valid *n* = 787**	**Valid *n* = 212**	**Valid *n* = 999**	**1.000†**
Absent	781 (99.2%)	212 (100.0%)	993 (99.4%)	
Arrhythmia	1 (0.1%)	0 (0.0%)	1 (0.1%)	
Hypertrophic cardiomyopathy	1 (0.1%)	0 (0.0%)	1 (0.1%)	
Unspecified heart disease	2 (0.3%)	0 (0.0%)	2 (0.2%)	
Mitral valve prolapse	2 (0.3%)	0 (0.0%)	2 (0.2%)	
**Hematological conditions and thrombophilias**	**Valid *n* = 787**	**Valid *n* = 212**	**Valid *n* = 999**	**0.150†**
Absent	778 (98.9%)	210 (99.1%)	988 (98.9%)	
Anemia	6 (0.8%)	0 (0.0%)	6 (0.6%)	
Spherocytosis	1 (0.1%)	0 (0.0%)	1 (0.1%)	
Sickle cell trait	1 (0.1%)	0 (0.0%)	1 (0.1%)	
Immune thrombocytopenia	0 (0.0%)	1 (0.5%)	1 (0.1%)	
Hereditary thrombophilia associated with factor V Leiden	1 (0.1%)	0 (0.0%)	1 (0.1%)	
Unspecified thrombophilia	0 (0.0%)	1 (0.5%)	1 (0.1%)	
**Autoimmune conditions**	**Valid *n* = 787**	**Valid *n* = 212**	**Valid *n* = 999**	**0.610†**
Absent	784 (99.6%)	211 (99.5%)	995 (99.6%)	
Rheumatoid arthritis	1 (0.1%)	0 (0.0%)	1 (0.1%)	
Unspecified autoimmune skin disease	1 (0.1%)	0 (0.0%)	1 (0.1%)	
Systemic lupus erythematosus	1 (0.1%)	1 (0.5%)	2 (0.2%)	
**Gastrointestinal and hepatobiliary conditions and hernias**	**Valid *n* = 787**	**Valid *n* = 212**	**Valid *n* = 999**	**1.000†**
Absent	780 (99.1%)	212 (100.0%)	992 (99.3%)	
Cholecystitis	1 (0.1%)	0 (0.0%)	1 (0.1%)	
Cholelithiasis; pancreatitis	1 (0.1%)	0 (0.0%)	1 (0.1%)	
Crohn disease	1 (0.1%)	0 (0.0%)	1 (0.1%)	
Gastroesophageal reflux disease	1 (0.1%)	0 (0.0%)	1 (0.1%)	
Gastritis	1 (0.1%)	0 (0.0%)	1 (0.1%)	
Hiatal hernia	1 (0.1%)	0 (0.0%)	1 (0.1%)	
Umbilical hernia	1 (0.1%)	0 (0.0%)	1 (0.1%)	
**Neurological conditions and other mental disorders**	**Valid *n* = 787**	**Valid *n* = 212**	**Valid *n* = 999**	**0.480†**
Absent	780 (99.1%)	210 (99.1%)	990 (99.1%)	
Anxiety	1 (0.1%)	0 (0.0%)	1 (0.1%)	
Epilepsy	2 (0.3%)	0 (0.0%)	2 (0.2%)	
Epilepsy; syncope	1 (0.1%)	0 (0.0%)	1 (0.1%)	

Markers	BMI recorded (*N* = 787)	BMI missing (*N* = 212)	Total (*N* = 999)	*p*-Value
Labyrinthitis	1 (0.1%)	0 (0.0%)	1 (0.1%)	
Bipolar disorder	1 (0.1%)	0 (0.0%)	1 (0.1%)	
Personality disorder	0 (0.0%)	1 (0.5%)	1 (0.1%)	
Panic disorder	1 (0.1%)	0 (0.0%)	1 (0.1%)	
Unspecified mood disorder	0 (0.0%)	1 (0.5%)	1 (0.1%)	
**Gynecological conditions, neoplasms, and masses of undetermined nature**	**Valid *n* = 787**	**Valid *n* = 212**	**Valid *n* = 999**	**0.776†**
Absent	780 (99.1%)	211 (99.5%)	991 (99.2%)	
Ovarian cyst	1 (0.1%)	0 (0.0%)	1 (0.1%)	
Laryngeal cancer	0 (0.0%)	1 (0.5%)	1 (0.1%)	
Uterine leiomyomatosis	2 (0.3%)	0 (0.0%)	2 (0.2%)	
Cervical intraepithelial neoplasia	1 (0.1%)	0 (0.0%)	1 (0.1%)	
Ovarian tumor of unspecified nature	1 (0.1%)	0 (0.0%)	1 (0.1%)	
Mass in the bladder region	1 (0.1%)	0 (0.0%)	1 (0.1%)	
Bicornuate uterus	1 (0.1%)	0 (0.0%)	1 (0.1%)	
**Other maternal infections**	**Valid *n* = 787**	**Valid *n* = 212**	**Valid *n* = 999**	**0.718†**
Absent	776 (98.6%)	209 (98.6%)	985 (98.6%)	
Perianal condyloma acuminatum	1 (0.1%)	0 (0.0%)	1 (0.1%)	
Hepatitis B	1 (0.1%)	0 (0.0%)	1 (0.1%)	
Hepatitis C	1 (0.1%)	0 (0.0%)	1 (0.1%)	
HIV infection	3 (0.4%)	1 (0.5%)	4 (0.4%)	
HPV infection	0 (0.0%)	1 (0.5%)	1 (0.1%)	
Pneumonia	1 (0.1%)	0 (0.0%)	1 (0.1%)	
Toxoplasmosis	4 (0.5%)	1 (0.5%)	5 (0.5%)	
**Other specified clinical conditions and medical history**	**Valid *n* = 787**	**Valid *n* = 212**	**Valid *n* = 999**	**0.819†**
Absent	779 (99.0%)	212 (100.0%)	991 (99.2%)	
Previous surgery	3 (0.4%)	0 (0.0%)	3 (0.3%)	
Fibromyalgia	2 (0.3%)	0 (0.0%)	2 (0.2%)	
Emotional lability	1 (0.1%)	0 (0.0%)	1 (0.1%)	
Rickets; scoliosis	1 (0.1%)	0 (0.0%)	1 (0.1%)	
Allergic rhinitis	1 (0.1%)	0 (0.0%)	1 (0.1%)	
**Delivery and reproductive history**				
**Mode of delivery**	**Valid *n* = 785**	**Valid *n* = 212**	**Valid *n* = 997**	**<0.001***
Cesarean delivery	580 (73.9%)	80 (37.7%)	660 (66.2%)	
Vaginal delivery	205 (26.1%)	132 (62.3%)	337 (33.8%)	
Missing data	2 (0.3%)	0 (0.0%)	2 (0.2%)	
**Episiotomy**	**Valid *n* = 783**	**Valid *n* = 210**	**Valid *n* = 993**	**<0.001#**
No	122 (15.6%)	78 (37.1%)	200 (20.1%)	
Yes	81 (10.3%)	52 (24.8%)	133 (13.4%)	
Not applicable	580 (74.1%)	80 (38.1%)	660 (66.5%)	
Missing data	4 (0.5%)	2 (0.9%)	6 (0.6%)	
**Recorded duration of surgery or labor (minutes)**	**Valid *n* = 760**	**Valid *n* = 206**	**Valid *n* = 966**	**<0.001#**
<30	44 (5.8%)	28 (13.6%)	72 (7.5%)	
30–60	418 (55.0%)	127 (61.7%)	545 (56.4%)	
60–120	277 (36.4%)	48 (23.3%)	325 (33.6%)	
>120	21 (2.8%)	3 (1.5%)	24 (2.5%)	
Missing data	27 (3.4%)	6 (2.8%)	33 (3.3%)	
**Perineal tear at vaginal delivery**	**Valid *n* = 780**	**Valid *n* = 210**	**Valid *n* = 990**	**<0.001#**
No tear	146 (18.7%)	91 (43.3%)	237 (23.9%)	
First degree	29 (3.7%)	25 (11.9%)	54 (5.5%)	
Second degree	25 (3.2%)	14 (6.7%)	39 (3.9%)	
Not applicable	580 (74.4%)	80 (38.1%)	660 (66.7%)	
Missing data	7 (0.9%)	2 (0.9%)	9 (0.9%)	
**Gravidity**	**Valid *n* = 706**	**Valid *n* = 181**	**Valid *n* = 887**	**0.116#**
Primigravida (1 pregnancy)	290 (41.1%)	74 (40.9%)	364 (41.0%)	
Secundigravida (2 pregnancies)	138 (19.5%)	47 (26.0%)	185 (20.9%)	
Multigravida (≥3 pregnancies)	278 (39.4%)	60 (33.1%)	338 (38.1%)	
Missing data	81 (10.3%)	31 (14.6%)	112 (11.2%)	

**Markers**	**BMI recorded (*N* = 787)**	**BMI missing (*N* = 212)**	**Total (*N* = 999)**	***p*-Value**
**Parity**	**Valid *n* = 772**	**Valid *n* = 206**	**Valid *n* = 978**	**0.165[#]**
1 delivery	349 (45.2%)	97 (47.1%)	446 (45.6%)	
2 deliveries	168 (21.8%)	54 (26.2%)	222 (22.7%)	
≥3 deliveries	255 (33.0%)	55 (26.7%)	310 (31.7%)	
Missing data	15 (1.9%)	6 (2.8%)	21 (2.1%)	
**Obstetric and fetal conditions**				
**Obstetric complications**	**Valid *n* = 787**	**Valid *n* = 212**	**Valid *n* = 999**	**<0.001[*]**
Absent	488 (62.0%)	167 (78.8%)	655 (65.6%)	
Present	299 (38.0%)	45 (21.2%)	344 (34.4%)	
**Gestational diabetes mellitus**	**Valid *n* = 787**	**Valid *n* = 212**	**Valid *n* = 999**	**0.011[*]**
Absent	662 (84.1%)	193 (91.0%)	855 (85.6%)	
Present	125 (15.9%)	19 (9.0%)	144 (14.4%)	
**Hypertensive disorders of pregnancy**	**Valid *n* = 787**	**Valid *n* = 212**	**Valid *n* = 999**	**0.006[*]**
Absent	657 (83.5%)	193 (91.0%)	850 (85.1%)	
Present	130 (16.5%)	19 (9.0%)	149 (14.9%)	
**Hypertensive disorders of pregnancy: recorded type**	**Valid *n* = 787**	**Valid *n* = 212**	**Valid *n* = 999**	**0.136[†]**
Absent	657 (83.5%)	193 (91.0%)	850 (85.1%)	
Hypertensive disorder of pregnancy	124 (15.8%)	18 (8.5%)	142 (14.2%)	
Hypertensive disorder of pregnancy; preeclampsia	1 (0.1%)	0 (0.0%)	1 (0.1%)	
Hypertensive disorder of pregnancy; HELLP syndrome	2 (0.3%)	0 (0.0%)	2 (0.2%)	
Eclampsia	1 (0.1%)	0 (0.0%)	1 (0.1%)	
Preeclampsia	2 (0.3%)	1 (0.5%)	3 (0.3%)	
**Fetal growth abnormalities**	**Valid *n* = 787**	**Valid *n* = 212**	**Valid *n* = 999**	**1.000[*]**
Absent	777 (98.7%)	210 (99.1%)	987 (98.8%)	
Present	10 (1.3%)	2 (0.9%)	12 (1.2%)	
**Fetal growth abnormalities: recorded type**	**Valid *n* = 787**	**Valid *n* = 212**	**Valid *n* = 999**	**0.877[†]**
Absent	777 (98.7%)	210 (99.1%)	987 (98.8%)	
Fetal macrosomia	3 (0.4%)	0 (0.0%)	3 (0.3%)	
Intrauterine growth restriction	7 (0.9%)	2 (0.9%)	9 (0.9%)	
**Intrauterine growth restriction**	**Valid *n* = 787**	**Valid *n* = 212**	**Valid *n* = 999**	**1.000[*]**
Absent	780 (99.1%)	210 (99.1%)	990 (99.1%)	
Present	7 (0.9%)	2 (0.9%)	9 (0.9%)	
**Fetal macrosomia**	**Valid *n* = 787**	**Valid *n* = 212**	**Valid *n* = 999**	**1.000[*]**
Absent	784 (99.6%)	212 (100.0%)	996 (99.7%)	
Present	3 (0.4%)	0 (0.0%)	3 (0.3%)	
**Fetal status abnormalities**	**Valid *n* = 787**	**Valid *n* = 212**	**Valid *n* = 999**	**1.000[*]**
Absent	766 (97.3%)	207 (97.6%)	973 (97.4%)	
Present	21 (2.7%)	5 (2.4%)	26 (2.6%)	
**Fetal status abnormalities: recorded type**	**Valid *n* = 787**	**Valid *n* = 212**	**Valid *n* = 999**	**1.000[†]**
Absent	766 (97.3%)	207 (97.6%)	973 (97.4%)	
Variable fetal heart rate decelerations	1 (0.1%)	0 (0.0%)	1 (0.1%)	
Fetal distress	18 (2.3%)	5 (2.4%)	23 (2.3%)	
Fetal distress; sustained fetal tachycardia	1 (0.1%)	0 (0.0%)	1 (0.1%)	
Fetal tachycardia	1 (0.1%)	0 (0.0%)	1 (0.1%)	
**Fetal distress**	**Valid *n* = 787**	**Valid *n* = 212**	**Valid *n* = 999**	**1.000[*]**
Absent	768 (97.6%)	207 (97.6%)	975 (97.6%)	
Present	19 (2.4%)	5 (2.4%)	24 (2.4%)	
**Other fetal heart rate abnormalities**	**Valid *n* = 787**	**Valid *n* = 212**	**Valid *n* = 999**	**1.000[†]**
Absent	784 (99.6%)	212 (100.0%)	996 (99.7%)	
Variable fetal heart rate decelerations	1 (0.1%)	0 (0.0%)	1 (0.1%)	
Fetal tachycardia	1 (0.1%)	0 (0.0%)	1 (0.1%)	
Sustained fetal tachycardia	1 (0.1%)	0 (0.0%)	1 (0.1%)	
**Amniotic fluid abnormalities**	**Valid *n* = 787**	**Valid *n* = 212**	**Valid *n* = 999**	**1.000***
Absent	771 (98.0%)	208 (98.1%)	979 (98.0%)	
Present	16 (2.0%)	4 (1.9%)	20 (2.0%)	
**Amniotic fluid abnormalities: recorded type**	**Valid *n* = 787**	**Valid *n* = 212**	**Valid *n* = 999**	**1.000[†]**
Absent	771 (98.0%)	208 (98.1%)	979 (98.0%)	
Oligohydramnios	12 (1.5%)	3 (1.4%)	15 (1.5%)	

Markers	BMI recorded (*N* = 787)	BMI missing (*N* = 212)	Total (*N* = 999)	*p*-Value
Polyhydramnios	4 (0.5%)	1 (0.5%)	5 (0.5%)	
**Polyhydramnios**	**Valid *n* = 787**	**Valid *n* = 212**	**Valid *n* = 999**	**1.000***
Absent	783 (99.5%)	211 (99.5%)	994 (99.5%)	
Present	4 (0.5%)	1 (0.5%)	5 (0.5%)	
**Oligohydramnios**	**Valid *n* = 787**	**Valid *n* = 212**	**Valid *n* = 999**	**1.000***
Absent	775 (98.5%)	209 (98.6%)	984 (98.5%)	
Present	12 (1.5%)	3 (1.4%)	15 (1.5%)	
**Rupture of membranes**	**Valid *n* = 787**	**Valid *n* = 212**	**Valid *n* = 999**	**0.782***
Absent	770 (97.8%)	209 (98.6%)	979 (98.0%)	
Present	17 (2.2%)	3 (1.4%)	20 (2.0%)	
**Fetal congenital anomalies**	**Valid *n* = 787**	**Valid *n* = 212**	**Valid *n* = 999**	**0.401†**
Absent	781 (99.2%)	210 (99.1%)	991 (99.2%)	
Bilateral renal agenesis	0 (0.0%)	1 (0.5%)	1 (0.1%)	
Ventricular septal defect; congenital clubfoot	1 (0.1%)	0 (0.0%)	1 (0.1%)	
Fetal gastroschisis	1 (0.1%)	0 (0.0%)	1 (0.1%)	
Pulmonary malformation	0 (0.0%)	1 (0.5%)	1 (0.1%)	
Unspecified fetal malformations	2 (0.3%)	0 (0.0%)	2 (0.2%)	
Fetal omphalocele	1 (0.1%)	0 (0.0%)	1 (0.1%)	
Down syndrome	1 (0.1%)	0 (0.0%)	1 (0.1%)	
**Placental abnormalities**	**Valid *n* = 787**	**Valid *n* = 212**	**Valid *n* = 999**	**0.758†**
Absent	782 (99.4%)	212 (100.0%)	994 (99.5%)	
Placenta accreta	1 (0.1%)	0 (0.0%)	1 (0.1%)	
Placental abruption	3 (0.4%)	0 (0.0%)	3 (0.3%)	
Placenta previa	1 (0.1%)	0 (0.0%)	1 (0.1%)	
**Other obstetric, delivery, and puerperal conditions**	**Valid *n* = 787**	**Valid *n* = 212**	**Valid *n* = 999**	**1.000†**
Absent	781 (99.2%)	211 (99.5%)	992 (99.3%)	
Uterine atony	1 (0.1%)	0 (0.0%)	1 (0.1%)	
Labor progression dystocia	1 (0.1%)	0 (0.0%)	1 (0.1%)	
Hemorrhage	1 (0.1%)	0 (0.0%)	1 (0.1%)	
Uterine hypotonia	1 (0.1%)	0 (0.0%)	1 (0.1%)	
Cervical insufficiency	2 (0.3%)	1 (0.5%)	3 (0.3%)	
**Perinatal characteristics and outcomes**				
**Gestational age at birth**	**Valid *n* = 780**	**Valid *n* = 211**	**Valid *n* = 991**	**0.735†**
Preterm (<37 weeks)	130 (16.7%)	41 (19.4%)	171 (17.3%)	
Term (37 weeks to 41 weeks and 6 days)	646 (82.8%)	169 (80.1%)	815 (82.2%)	
Post-term (≥42 completed weeks)	4 (0.5%)	1 (0.5%)	5 (0.5%)	
Missing data	7 (0.9%)	1 (0.5%)	8 (0.8%)	
**Birth weight classification**	**Valid *n* = 778**	**Valid *n* = 212**	**Valid *n* = 990**	**0.436†**
Extremely low birth weight (<1000 g)	8 (1.0%)	5 (2.4%)	13 (1.3%)	
Very low birth weight (1000–1499 g)	11 (1.4%)	5 (2.4%)	16 (1.6%)	
Low birth weight (1500–2499 g)	88 (11.3%)	24 (11.3%)	112 (11.3%)	
Normal birth weight (2500–3999 g)	632 (81.2%)	170 (80.2%)	802 (81.0%)	
Macrosomia (≥4000 g)	39 (5.0%)	8 (3.8%)	47 (4.7%)	
Missing data	9 (1.1%)	0 (0.0%)	9 (0.9%)	
**Birth weight for gestational age**	**Valid *n* = 783**	**Valid *n* = 212**	**Valid *n* = 995**	**0.399#**
Small for gestational age	53 (6.8%)	20 (9.4%)	73 (7.3%)	
Appropriate for gestational age	660 (84.3%)	175 (82.5%)	835 (83.9%)	
Large for gestational age	70 (8.9%)	17 (8.0%)	87 (8.7%)	
Missing data	4 (0.5%)	0 (0.0%)	4 (0.4%)	
**Apgar score category at 5 minutes**	**Valid *n* = 781**	**Valid *n* = 207**	**Valid *n* = 988**	**0.335†**
Low (0–3)	4 (0.5%)	2 (1.0%)	6 (0.6%)	
Moderately abnormal (4–6)	15 (1.9%)	7 (3.4%)	22 (2.2%)	
Reassuring (7–10)	754 (96.5%)	194 (93.7%)	948 (96.0%)	
Not applicable	8 (1.0%)	4 (1.9%)	12 (1.2%)	
Missing data	6 (0.8%)	5 (2.4%)	11 (1.1%)	
**Composite adverse perinatal outcome**	**Valid *n* = 781**	**Valid *n* = 209**	**Valid *n* = 990**	**0.022***
No	747 (95.6%)	191 (91.4%)	938 (94.7%)	
Yes	34 (4.4%)	18 (8.6%)	52 (5.3%)	

Markers		BMI recorded (N = 787)	BMI missing (N = 212)	Total (N = 999)	p-Value
	Missing data	6 (0.8%)	3 (1.4%)	9 (0.9%)	
**NICU admission or death**		**Valid n = 787**	**Valid n = 212**	**Valid n = 999**	**0.020** *
	No	769 (97.7%)	200 (94.3%)	969 (97.0%)	
	Yes	18 (2.3%)	12 (5.7%)	30 (3.0%)	
**NICU admission**		**Valid n = 787**	**Valid n = 212**	**Valid n = 999**	**0.035** *
	No	777 (98.7%)	204 (96.2%)	981 (98.2%)	
	Yes	10 (1.3%)	8 (3.8%)	18 (1.8%)	
**Death**		**Valid n = 787**	**Valid n = 212**	**Valid n = 999**	**0.195** †
	No	779 (99.0%)	208 (98.1%)	987 (98.8%)	
	Yes (fetal death)	8 (1.0%)	3 (1.4%)	11 (1.1%)	
	Yes (stillbirth)	0 (0.0%)	1 (0.5%)	1 (0.1%)	
**Previous pregnancy losses**		**Valid n = 787**	**Valid n = 212**	**Valid n = 999**	**0.672** †
	1 pregnancy loss	9 (1.1%)	1 (0.5%)	10 (1.0%)	
	2 pregnancy losses	2 (0.3%)	0 (0.0%)	2 (0.2%)	
	3 pregnancy losses	2 (0.3%)	0 (0.0%)	2 (0.2%)	
	No	774 (98.3%)	211 (99.5%)	985 (98.6%)	

BMI, body mass index; HELLP, hemolysis, elevated liver enzymes, and low platelet count; HIV, human immunodeficiency virus; HPV, human papillomavirus; NICU, neonatal intensive care unit; PhD, Doctor of Philosophy. n, category frequency or number of events, as specified; N, group size or analysis denominator. *, Two-sided Fisher exact test. $^{\#}$, Pearson chi-square test without continuity correction. †, Conditional Monte Carlo test of the Pearson statistic with 9999 random tables and fixed margins (seed 20261001). A single global p-Value is reported per marker, excluding missing data. Tests are exploratory and unadjusted for multiple comparisons.

Not applicable is retained as an observed category. Consequently, the global tests for episiotomy and perineal tear also reflect differences in delivery route and applicability. The surgery/labor duration field retains the intervals recorded in the database and does not identify a specific stage of labor. Multiple diagnoses recorded in one category are separated by semicolons and were not counted as separate participants. Fetal death and stillbirth labels are displayed as recorded. The composite adverse perinatal outcome comprises NICU admission, recorded fetal death or stillbirth, or a 5-min Apgar score <7 among liveborn infants. The recorded BMI was not imputed.

Table S9. Comparison of participants with and without recorded maternal body mass index (BMI) after multiple imputation among 999 singleton pregnancies.

Values are rounded mean frequencies (mean percentages) across 50 imputations. The largest remainder method preserves group totals of 787 and 212 for every marker; total frequencies sum the rounded group counts (*N* = 999). Counts are estimated, not observed. Percentages and global tests use unrounded data. Maternal BMI and group membership were not imputed.

Markers		BMI recorded (*N* = 787)	BMI missing (*N* = 212)	Total (*N* = 999)	*p*-Value
**Sociodemographic characteristics and lifestyle**					
**Race**					**0.139‡**
	White	558 (71.0%)	138 (65.2%)	696 (69.7%)	
	Mixed race (*Pardo*)	189 (24.0%)	56 (26.6%)	245 (24.5%)	
	Black	40 (5.1%)	18 (8.2%)	58 (5.7%)	
**Maternal age (years)**					**0.297†**
	15-19	79 (10.0%)	31 (14.6%)	110 (11.0%)	
	20-24	190 (24.1%)	59 (27.8%)	249 (24.9%)	
	25-29	199 (25.3%)	51 (24.1%)	250 (25.0%)	
	30-34	160 (20.3%)	38 (17.9%)	198 (19.8%)	
	35-39	116 (14.7%)	25 (11.8%)	141 (14.1%)	
	40-44	40 (5.1%)	8 (3.8%)	48 (4.8%)	
	45-49	3 (0.4%)	0 (0.0%)	3 (0.3%)	
**Marital status**					**0.886‡,a**
	Married	291 (36.9%)	72 (34.0%)	363 (36.3%)	
	Divorced	18 (2.4%)	5 (2.5%)	23 (2.4%)	
	Single	430 (54.6%)	122 (57.5%)	552 (55.2%)	
	Stable union	48 (6.1%)	13 (6.0%)	61 (6.1%)	
**Educational attainment**					**0.913‡,a**
	Illiterate	2 (0.3%)	2 (0.7%)	4 (0.4%)	
	Incomplete elementary school	61 (7.7%)	15 (7.0%)	76 (7.6%)	
	Completed elementary school	128 (16.3%)	31 (14.8%)	159 (16.0%)	
	Incomplete high school	71 (9.0%)	19 (9.0%)	90 (9.0%)	
	Completed high school	414 (52.6%)	122 (57.6%)	536 (53.7%)	
	Incomplete higher education	28 (3.6%)	5 (2.3%)	33 (3.3%)	
	Completed higher education	82 (10.4%)	18 (8.6%)	100 (10.0%)	
	Doctorate/PhD	1 (0.1%)	0 (0.0%)	1 (0.1%)	
**Smoking**					**0.880***
	No	731 (92.9%)	198 (93.4%)	929 (93.0%)	
	Yes	56 (7.1%)	14 (6.6%)	70 (7.0%)	
**Alcohol consumption**					**0.239***
	No	763 (97.0%)	209 (98.6%)	972 (97.3%)	
	Yes	24 (3.0%)	3 (1.4%)	27 (2.7%)	
**Illicit drug use**					**0.698***
	No	778 (98.9%)	211 (99.5%)	989 (99.0%)	
	Yes	9 (1.1%)	1 (0.5%)	10 (1.0%)	
**Maternal clinical conditions**					
**Maternal clinical conditions (any)**					**0.015***
	Absent	557 (70.8%)	168 (79.2%)	725 (72.6%)	
	Present	230 (29.2%)	44 (20.8%)	274 (27.4%)	
**Chronic hypertension**					**0.040***
	Absent	711 (90.3%)	201 (94.8%)	912 (91.3%)	
	Present	76 (9.7%)	11 (5.2%)	87 (8.7%)	
**Hypothyroidism**					**0.742***
	Absent	740 (94.0%)	201 (94.8%)	941 (94.2%)	
	Present	47 (6.0%)	11 (5.2%)	58 (5.8%)	
**Urinary tract infection**					**0.345***
	Absent	755 (95.9%)	200 (94.3%)	955 (95.6%)	
	Present	32 (4.1%)	12 (5.7%)	44 (4.4%)	
**Asthma**					**0.123***
	Absent	758 (96.3%)	209 (98.6%)	967 (96.8%)	
	Present	29 (3.7%)	3 (1.4%)	32 (3.2%)	

Markers		BMI recorded (*N* = 787)	BMI missing (*N* = 212)	Total (*N* = 999)	*p*-Value
**Pre-existing diabetes mellitus**					**0.574***
	Absent	773 (98.2%)	207 (97.6%)	980 (98.1%)	
	Present	14 (1.8%)	5 (2.4%)	19 (1.9%)	
**Type of pre-existing diabetes mellitus**					**0.901†**
	Absent	773 (98.2%)	207 (97.6%)	980 (98.1%)	
	Type 1 diabetes mellitus	2 (0.3%)	1 (0.5%)	3 (0.3%)	
	Type 2 diabetes mellitus	12 (1.5%)	4 (1.9%)	16 (1.6%)	
**Syphilis**					**0.450***
	Absent	780 (99.1%)	209 (98.6%)	989 (99.0%)	
	Present	7 (0.9%)	3 (1.4%)	10 (1.0%)	
**Depression**					**1.000***
	Absent	779 (99.0%)	210 (99.1%)	989 (99.0%)	
	Present	8 (1.0%)	2 (0.9%)	10 (1.0%)	
**Noninfectious renal and urological conditions**					**0.401†**
	Absent	781 (99.2%)	210 (99.1%)	991 (99.2%)	
	Renal atrophy	1 (0.1%)	0 (0.0%)	1 (0.1%)	
	Ureteropelvic junction stenosis	0 (0.0%)	1 (0.5%)	1 (0.1%)	
	Glomerulonephritis	1 (0.1%)	0 (0.0%)	1 (0.1%)	
	Hydronephrosis	1 (0.1%)	0 (0.0%)	1 (0.1%)	
	Nephrolithiasis	2 (0.3%)	0 (0.0%)	2 (0.2%)	
	Unspecified nephropathy	0 (0.0%)	1 (0.5%)	1 (0.1%)	
	Ureterocele; hydronephrosis	1 (0.1%)	0 (0.0%)	1 (0.1%)	
**Other cardiovascular conditions**					**1.000†**
	Absent	781 (99.2%)	212 (100.0%)	993 (99.4%)	
	Arrhythmia	1 (0.1%)	0 (0.0%)	1 (0.1%)	
	Hypertrophic cardiomyopathy	1 (0.1%)	0 (0.0%)	1 (0.1%)	
	Unspecified heart disease	2 (0.3%)	0 (0.0%)	2 (0.2%)	
	Mitral valve prolapse	2 (0.3%)	0 (0.0%)	2 (0.2%)	
**Hematological conditions and thrombophilias**					**0.150†**
	Absent	778 (98.9%)	210 (99.1%)	988 (98.9%)	
	Anemia	6 (0.8%)	0 (0.0%)	6 (0.6%)	
	Spherocytosis	1 (0.1%)	0 (0.0%)	1 (0.1%)	
	Sickle cell trait	1 (0.1%)	0 (0.0%)	1 (0.1%)	
	Immune thrombocytopenia	0 (0.0%)	1 (0.5%)	1 (0.1%)	
	Hereditary thrombophilia associated with factor V Leiden	1 (0.1%)	0 (0.0%)	1 (0.1%)	
	Unspecified thrombophilia	0 (0.0%)	1 (0.5%)	1 (0.1%)	
**Autoimmune conditions**					**0.610†**
	Absent	784 (99.6%)	211 (99.5%)	995 (99.6%)	
	Rheumatoid arthritis	1 (0.1%)	0 (0.0%)	1 (0.1%)	
	Unspecified autoimmune skin disease	1 (0.1%)	0 (0.0%)	1 (0.1%)	
	Systemic lupus erythematosus	1 (0.1%)	1 (0.5%)	2 (0.2%)	
**Gastrointestinal and hepatobiliary conditions and hernias**					**1.000†**
	Absent	780 (99.1%)	212 (100.0%)	992 (99.3%)	
	Cholecystitis	1 (0.1%)	0 (0.0%)	1 (0.1%)	
	Cholelithiasis; pancreatitis	1 (0.1%)	0 (0.0%)	1 (0.1%)	
	Crohn disease	1 (0.1%)	0 (0.0%)	1 (0.1%)	
	Gastroesophageal reflux disease	1 (0.1%)	0 (0.0%)	1 (0.1%)	
	Gastritis	1 (0.1%)	0 (0.0%)	1 (0.1%)	
	Hiatal hernia	1 (0.1%)	0 (0.0%)	1 (0.1%)	
	Umbilical hernia	1 (0.1%)	0 (0.0%)	1 (0.1%)	
**Neurological conditions and other mental disorders**					**0.480†**
	Absent	780 (99.1%)	210 (99.1%)	990 (99.1%)	
	Anxiety	1 (0.1%)	0 (0.0%)	1 (0.1%)	
	Epilepsy	2 (0.3%)	0 (0.0%)	2 (0.2%)	
	Epilepsy; syncope	1 (0.1%)	0 (0.0%)	1 (0.1%)	
	Labyrinthitis	1 (0.1%)	0 (0.0%)	1 (0.1%)	
	Bipolar disorder	1 (0.1%)	0 (0.0%)	1 (0.1%)	
	Personality disorder	0 (0.0%)	1 (0.5%)	1 (0.1%)	

Markers	BMI recorded (*N* = 787)	BMI missing (*N* = 212)	Total (*N* = 999)	*p*-Value
Panic disorder	1 (0.1%)	0 (0.0%)	1 (0.1%)	
Unspecified mood disorder	0 (0.0%)	1 (0.5%)	1 (0.1%)	
**Gynecological conditions, neoplasms, and masses of undetermined nature**				**0.776†**
Absent	780 (99.1%)	211 (99.5%)	991 (99.2%)	
Ovarian cyst	1 (0.1%)	0 (0.0%)	1 (0.1%)	
Laryngeal cancer	0 (0.0%)	1 (0.5%)	1 (0.1%)	
Uterine leiomyomatosis	2 (0.3%)	0 (0.0%)	2 (0.2%)	
Cervical intraepithelial neoplasia	1 (0.1%)	0 (0.0%)	1 (0.1%)	
Ovarian tumor of unspecified nature	1 (0.1%)	0 (0.0%)	1 (0.1%)	
Mass in the bladder region	1 (0.1%)	0 (0.0%)	1 (0.1%)	
Bicornuate uterus	1 (0.1%)	0 (0.0%)	1 (0.1%)	
**Other maternal infections**				**0.718†**
Absent	776 (98.6%)	209 (98.6%)	985 (98.6%)	
Perianal condyloma acuminatum	1 (0.1%)	0 (0.0%)	1 (0.1%)	
Hepatitis B	1 (0.1%)	0 (0.0%)	1 (0.1%)	
Hepatitis C	1 (0.1%)	0 (0.0%)	1 (0.1%)	
HIV infection	3 (0.4%)	1 (0.5%)	4 (0.4%)	
HPV infection	0 (0.0%)	1 (0.5%)	1 (0.1%)	
Pneumonia	1 (0.1%)	0 (0.0%)	1 (0.1%)	
Toxoplasmosis	4 (0.5%)	1 (0.5%)	5 (0.5%)	
**Other specified clinical conditions and medical history**				**0.819†**
Absent	779 (99.0%)	212 (100.0%)	991 (99.2%)	
Previous surgery	3 (0.4%)	0 (0.0%)	3 (0.3%)	
Fibromyalgia	2 (0.3%)	0 (0.0%)	2 (0.2%)	
Emotional lability	1 (0.1%)	0 (0.0%)	1 (0.1%)	
Rickets; scoliosis	1 (0.1%)	0 (0.0%)	1 (0.1%)	
Allergic rhinitis	1 (0.1%)	0 (0.0%)	1 (0.1%)	
**Delivery and reproductive history**				
**Mode of delivery**				**<0.001‡**
Cesarean delivery	580 (73.8%)	80 (37.7%)	660 (66.1%)	
Vaginal delivery	207 (26.2%)	132 (62.3%)	339 (33.9%)	
**Episiotomy**				**<0.001‡**
No	124 (15.7%)	79 (37.4%)	203 (20.3%)	
Yes	83 (10.5%)	53 (24.8%)	136 (13.6%)	
Not applicable	580 (73.8%)	80 (37.7%)	660 (66.1%)	
**Recorded duration of surgery or labor (minutes)**				**<0.001‡**
<30	48 (6.1%)	29 (13.7%)	77 (7.7%)	
30–60	434 (55.2%)	131 (61.6%)	565 (56.5%)	
60–120	284 (36.0%)	49 (23.2%)	333 (33.3%)	
>120	21 (2.7%)	3 (1.4%)	24 (2.4%)	
**Perineal tear at vaginal delivery**				**<0.001‡**
No tear	151 (19.2%)	93 (43.7%)	244 (24.4%)	
First degree	30 (3.8%)	25 (11.9%)	55 (5.5%)	
Second degree	26 (3.2%)	14 (6.7%)	40 (4.0%)	
Not applicable	580 (73.8%)	80 (37.7%)	660 (66.1%)	
**Gravidity**				**0.043‡**
Primigravida (1 pregnancy)	333 (42.4%)	89 (42.0%)	422 (42.3%)	
Secundigravida (2 pregnancies)	169 (21.4%)	62 (29.0%)	231 (23.0%)	
Multigravida (≥3 pregnancies)	285 (36.2%)	61 (29.0%)	346 (34.7%)	
**Parity**				**0.122‡**
1 delivery	357 (45.4%)	100 (47.2%)	457 (45.8%)	
2 deliveries	173 (21.9%)	57 (26.8%)	230 (22.9%)	
≥3 deliveries	257 (32.7%)	55 (26.0%)	312 (31.3%)	
**Obstetric and fetal conditions**				**<0.001***
**Obstetric complications (any)**				
Absent	488 (62.0%)	167 (78.8%)	655 (65.6%)	
Present	299 (38.0%)	45 (21.2%)	344 (34.4%)	

Markers		BMI recorded (N = 787)	BMI missing (N = 212)	Total (N = 999)	p-Value
**Gestational diabetes mellitus**					**0.011***
	Absent	662 (84.1%)	193 (91.0%)	855 (85.6%)	
	Present	125 (15.9%)	19 (9.0%)	144 (14.4%)	
**Hypertensive disorders of pregnancy**					**0.006***
	Absent	657 (83.5%)	193 (91.0%)	850 (85.1%)	
	Present	130 (16.5%)	19 (9.0%)	149 (14.9%)	
**Hypertensive disorders of pregnancy: recorded type**					**0.136†**
	Absent	657 (83.5%)	193 (91.0%)	850 (85.1%)	
	Hypertensive disorder of pregnancy	124 (15.8%)	18 (8.5%)	142 (14.2%)	
	Hypertensive disorder of pregnancy; preeclampsia	1 (0.1%)	0 (0.0%)	1 (0.1%)	
	Hypertensive disorder of pregnancy; HELLP syndrome	2 (0.3%)	0 (0.0%)	2 (0.2%)	
	Eclampsia	1 (0.1%)	0 (0.0%)	1 (0.1%)	
	Preeclampsia	2 (0.3%)	1 (0.5%)	3 (0.3%)	
**Fetal growth abnormalities**					**1.000***
	Absent	777 (98.7%)	210 (99.1%)	987 (98.8%)	
	Present	10 (1.3%)	2 (0.9%)	12 (1.2%)	
**Fetal growth abnormalities: recorded type**					**0.887†**
	Absent	777 (98.7%)	210 (99.1%)	987 (98.8%)	
	Fetal macrosomia	3 (0.4%)	0 (0.0%)	3 (0.3%)	
	Intrauterine growth restriction	7 (0.9%)	2 (0.9%)	9 (0.9%)	
**Intrauterine growth restriction**					**1.000***
	Absent	780 (99.1%)	210 (99.1%)	990 (99.1%)	
	Present	7 (0.9%)	2 (0.9%)	9 (0.9%)	
**Fetal macrosomia**					**1.000***
	Absent	784 (99.6%)	212 (100.0%)	996 (99.7%)	
	Present	3 (0.4%)	0 (0.0%)	3 (0.3%)	
**Fetal status abnormalities**					**1.000***
	Absent	766 (97.3%)	207 (97.6%)	973 (97.4%)	
	Present	21 (2.7%)	5 (2.4%)	26 (2.6%)	
**Fetal status abnormalities: recorded type**					**1.000†**
	Absent	766 (97.3%)	207 (97.6%)	973 (97.4%)	
	Variable fetal heart rate decelerations	1 (0.1%)	0 (0.0%)	1 (0.1%)	
	Fetal distress	18 (2.3%)	5 (2.4%)	23 (2.3%)	
	Fetal distress; sustained fetal tachycardia	1 (0.1%)	0 (0.0%)	1 (0.1%)	
	Fetal tachycardia	1 (0.1%)	0 (0.0%)	1 (0.1%)	
**Fetal distress**					**1.000***
	Absent	768 (97.6%)	207 (97.6%)	975 (97.6%)	
	Present	19 (2.4%)	5 (2.4%)	24 (2.4%)	
**Other fetal heart rate abnormalities**					**1.000†**
	Absent	784 (99.6%)	212 (100.0%)	996 (99.7%)	
	Variable fetal heart rate decelerations	1 (0.1%)	0 (0.0%)	1 (0.1%)	
	Fetal tachycardia	1 (0.1%)	0 (0.0%)	1 (0.1%)	
	Sustained fetal tachycardia	1 (0.1%)	0 (0.0%)	1 (0.1%)	
**Amniotic fluid abnormalities**					**1.000***
	Absent	771 (98.0%)	208 (98.1%)	979 (98.0%)	
	Present	16 (2.0%)	4 (1.9%)	20 (2.0%)	
**Amniotic fluid abnormalities: recorded type**					**1.000†**
	Absent	771 (98.0%)	208 (98.1%)	979 (98.0%)	
	Oligohydramnios	12 (1.5%)	3 (1.4%)	15 (1.5%)	
	Polyhydramnios	4 (0.5%)	1 (0.5%)	5 (0.5%)	
**Polyhydramnios**					**1.000***
	Absent	783 (99.5%)	211 (99.5%)	994 (99.5%)	
	Present	4 (0.5%)	1 (0.5%)	5 (0.5%)	
**Oligohydramnios**					**1.000***
	Absent	775 (98.5%)	209 (98.6%)	984 (98.5%)	
	Present	12 (1.5%)	3 (1.4%)	15 (1.5%)	
**Rupture of membranes**					**0.782***
	Absent	770 (97.8%)	209 (98.6%)	979 (98.0%)	

Markers	BMI recorded (N = 787)	BMI missing (N = 212)	Total (N = 999)	p-Value
Present	17 (2.2%)	3 (1.4%)	20 (2.0%)	
**Fetal congenital anomalies**				**0.410†**
Absent	781 (99.2%)	210 (99.1%)	991 (99.2%)	
Bilateral renal agenesis	0 (0.0%)	1 (0.5%)	1 (0.1%)	
Ventricular septal defect; congenital clubfoot	1 (0.1%)	0 (0.0%)	1 (0.1%)	
Fetal gastroschisis	1 (0.1%)	0 (0.0%)	1 (0.1%)	
Pulmonary malformation	0 (0.0%)	1 (0.5%)	1 (0.1%)	
Unspecified fetal malformations	2 (0.3%)	0 (0.0%)	2 (0.2%)	
Fetal omphalocele	1 (0.1%)	0 (0.0%)	1 (0.1%)	
Down syndrome	1 (0.1%)	0 (0.0%)	1 (0.1%)	
**Placental abnormalities**				**0.758†**
Absent	782 (99.4%)	212 (100.0%)	994 (99.5%)	
Placenta accreta	1 (0.1%)	0 (0.0%)	1 (0.1%)	
Placental abruption	3 (0.4%)	0 (0.0%)	3 (0.3%)	
Placenta previa	1 (0.1%)	0 (0.0%)	1 (0.1%)	
**Other obstetric, delivery, and puerperal conditions**				**1.000†**
Absent	781 (99.2%)	211 (99.5%)	992 (99.3%)	
Uterine atony	1 (0.1%)	0 (0.0%)	1 (0.1%)	
Labor progression dystocia	1 (0.1%)	0 (0.0%)	1 (0.1%)	
Hemorrhage	1 (0.1%)	0 (0.0%)	1 (0.1%)	
Uterine hypotonia	1 (0.1%)	0 (0.0%)	1 (0.1%)	
Cervical insufficiency	2 (0.3%)	1 (0.5%)	3 (0.3%)	
**Perinatal characteristics and outcomes**				
**Gestational age at birth**				**0.639‡,a**
Preterm (<37 weeks)	132 (16.8%)	42 (19.6%)	174 (17.4%)	
Term (37 weeks to 41 weeks and 6 days)	651 (82.7%)	169 (79.9%)	820 (82.1%)	
Post-term (≥42 completed weeks)	4 (0.5%)	1 (0.5%)	5 (0.5%)	
**Birth weight classification**				**0.550‡,a**
Extremely low birth weight (<1000 g)	9 (1.1%)	5 (2.4%)	14 (1.4%)	
Very low birth weight (1000–1499 g)	12 (1.5%)	5 (2.4%)	17 (1.7%)	
Low birth weight (1500–2499 g)	89 (11.3%)	24 (11.3%)	113 (11.3%)	
Normal birth weight (2500–3999 g)	638 (81.1%)	170 (80.2%)	808 (80.9%)	
Macrosomia (≥4000 g)	39 (5.0%)	8 (3.8%)	47 (4.7%)	
**Birth weight for gestational age**				**0.452‡**
Small for gestational age	55 (6.9%)	20 (9.4%)	75 (7.5%)	
Appropriate for gestational age	662 (84.1%)	175 (82.5%)	837 (83.8%)	
Large for gestational age	70 (8.9%)	17 (8.0%)	87 (8.7%)	
**Apgar score category at 5 minutes**				**0.244‡,a**
Low (0–3)	4 (0.5%)	2 (1.2%)	6 (0.7%)	
Moderately abnormal (4–6)	15 (1.9%)	8 (3.6%)	23 (2.3%)	
Reassuring (7–10)	760 (96.5%)	198 (93.3%)	958 (95.8%)	
Not applicable	8 (1.0%)	4 (1.9%)	12 (1.2%)	
**Composite adverse perinatal outcome**				**0.012‡**
No	753 (95.6%)	194 (91.3%)	947 (94.7%)	
Yes	34 (4.4%)	18 (8.7%)	52 (5.3%)	
**NICU admission or death**				**0.020***
No	769 (97.7%)	200 (94.3%)	969 (97.0%)	
Yes	18 (2.3%)	12 (5.7%)	30 (3.0%)	
**NICU admission**				**0.035***
No	777 (98.7%)	204 (96.2%)	981 (98.2%)	
Yes	10 (1.3%)	8 (3.8%)	18 (1.8%)	
**Death**				**0.195†**
No	779 (99.0%)	208 (98.1%)	987 (98.8%)	
Yes (fetal death)	8 (1.0%)	3 (1.4%)	11 (1.1%)	
Yes (stillbirth)	0 (0.0%)	1 (0.5%)	1 (0.1%)	
**Previous pregnancy losses**				**0.672†**
1 pregnancy loss	9 (1.1%)	1 (0.5%)	10 (1.0%)	
2 pregnancy losses	2 (0.3%)	0 (0.0%)	2 (0.2%)	

Markers	BMI recorded (*N* = 787)	BMI missing (*N* = 212)	Total (*N* = 999)	*p*-Value
3 pregnancy losses	2 (0.3%)	0 (0.0%)	2 (0.2%)	
No	774 (98.3%)	211 (99.5%)	985 (98.6%)	

BMI, body mass index; HELLP, hemolysis, elevated liver enzymes, and low platelet count; HIV, human immunodeficiency virus; HPV, human papillomavirus; NICU, neonatal intensive care unit; PhD, Doctor of Philosophy. n, category frequency or number of events, as specified; *N*, group size or analysis denominator. Rounding was performed separately within the groups displayed in each table. Consequently, aggregated rounded counts may differ by one participant between tables that use different grouping structures; percentages and statistical tests are based on unrounded data. One global *p*-Value per marker. Unchanged markers retain the test and *p*-Value from Table S8: [*], two-sided Fisher exact; [†], conditional Monte Carlo Pearson (9999 tables; fixed margins; seed 20261001). [‡], Pearson chi-square statistics pooled across 50 imputations using the D2 procedure (Enders formulation; approximate F distribution). [a], At least one expected cell count was <5 in at least one imputation; sparse-category pooled tests should be interpreted cautiously. Tests are exploratory and unadjusted for multiple comparisons.

Not applicable remains in denominators; combined diagnoses and death labels are retained as recorded. The composite comprises NICU admission, recorded fetal death or stillbirth, or a 5-min Apgar score <7 among liveborn infants. Imputation preceded exclusion of 13 twin pregnancies and preserved structural non-applicability. These comparisons cannot exclude selection bias from missing BMI.

Table S10. Exploratory binary associations of recorded maternal body mass index (BMI) with all applicable indicators, including less frequent diagnoses and outcome components.
The association population contains 760 women unless an applicable subgroup is specified. Normal weight is the reference. Broad heterogeneous diagnostic groups are compared as any recorded condition versus absent solely for exploratory analysis; diagnoses retain their individual labels in Tables S6–S7. Percentages use the mean of completed-dataset proportions.

Marker or comparison	Normal weight *n* / *N* (%)	Overweight *n* / *N* (%)	Obesity *n* / *N* (%)	Overweight OR (95% CI)	Obesity OR (95% CI)	Global *p*-Value
Smoking	24 / 282 (8.5)	12 / 210 (5.7)	17 / 268 (6.3)	0.65 (0.32–1.33)	0.73 (0.38–1.39)	0.426[#]
Alcohol consumption	12 / 282 (4.3)	3 / 210 (1.4)	6 / 268 (2.2)	0.33 (0.09–1.17)	0.52 (0.19–1.39)	0.135[#]
Illicit drug use	5 / 282 (1.8)	1 / 210 (0.5)	1 / 268 (0.4)	0.27 (0.03–2.29)	0.21 (0.02–1.79)	0.190[†]
Maternal clinical conditions	55 / 282 (19.5)	59 / 210 (28.1)	113 / 268 (42.2)	1.61 (1.06–2.46)	3.01 (2.05–4.41)	<0.001[#]
Chronic hypertension	8 / 282 (2.8)	10 / 210 (4.8)	58 / 268 (21.6)	1.71 (0.66–4.42)	9.46 (4.42–20.24)	<0.001[#]
Hypothyroidism	11 / 282 (3.9)	11 / 210 (5.2)	23 / 268 (8.6)	1.36 (0.58–3.20)	2.31 (1.10–4.84)	0.059[#]
Urinary tract infection	9 / 282 (3.2)	12 / 210 (5.7)	10 / 268 (3.7)	1.84 (0.76–4.45)	1.18 (0.47–2.94)	0.352[#]
Asthma	8 / 282 (2.8)	9 / 210 (4.3)	12 / 268 (4.5)	1.53 (0.58–4.04)	1.61 (0.65–3.99)	0.554[#]
Pre-existing diabetes mellitus	2 / 282 (0.7)	5 / 210 (2.4)	7 / 268 (2.6)	3.41 (0.66–17.77)	3.75 (0.77–18.24)	0.203[†]
Syphilis	4 / 282 (1.4)	1 / 210 (0.5)	2 / 268 (0.7)	0.33 (0.04–3.00)	0.52 (0.09–2.88)	0.629[†]
Depression	0 / 282 (0.0)	6 / 210 (2.9)	2 / 268 (0.7)	17.96 (1.01–320.58)[¶]	5.30 (0.25–110.91)[¶]	0.008[†]
Obstetric complications	64 / 282 (22.7)	81 / 210 (38.6)	147 / 268 (54.9)	2.14 (1.44–3.17)	4.14 (2.86–5.98)	<0.001[#]
Gestational diabetes mellitus	28 / 282 (9.9)	37 / 210 (17.6)	59 / 268 (22.0)	1.94 (1.14–3.29)	2.56 (1.58–4.16)	<0.001[#]
Hypertensive disorders of pregnancy	20 / 282 (7.1)	25 / 210 (11.9)	84 / 268 (31.3)	1.77 (0.95–3.28)	5.98 (3.55–10.09)	<0.001[#]
Fetal growth abnormalities	0 / 282 (0.0)	3 / 210 (1.4)	6 / 268 (2.2)	9.53 (0.49–185.50)[¶]	13.99 (0.78–249.57)[¶]	0.053[†]
Intrauterine growth restriction	0 / 282 (0.0)	3 / 210 (1.4)	3 / 268 (1.1)	9.53 (0.49–185.50)[¶]	7.45 (0.38–144.88)[¶]	0.144[†]
Fetal macrosomia	0 / 282 (0.0)	0 / 210 (0.0)	3 / 268 (1.1)	1.34 (0.03–67.91)[¶]	7.45 (0.38–144.88)[¶]	0.066[†]
Fetal status abnormalities	11 / 282 (3.9)	6 / 210 (2.9)	2 / 268 (0.7)	0.72 (0.26–1.99)	0.19 (0.04–0.84)	0.056[#]
Fetal distress	10 / 282 (3.5)	5 / 210 (2.4)	2 / 268 (0.7)	0.66 (0.22–1.97)	0.20 (0.04–0.94)	0.079[†]
Amniotic fluid abnormalities	3 / 282 (1.1)	6 / 210 (2.9)	5 / 268 (1.9)	2.74 (0.68–11.07)	1.77 (0.42–7.47)	0.344[†]
Polyhydramnios	1 / 282 (0.4)	0 / 210 (0.0)	2 / 268 (0.7)	0.45 (0.02–11.00)[¶]	2.11 (0.19–23.44)	0.638[†]
Oligohydramnios	2 / 282 (0.7)	6 / 210 (2.9)	3 / 268 (1.1)	4.12 (0.82–20.61)	1.58 (0.26–9.56)	0.132[†]
Rupture of membranes	5 / 282 (1.8)	5 / 210 (2.4)	6 / 268 (2.2)	1.35 (0.39–4.73)	1.27 (0.38–4.21)	0.903[†]
Composite adverse perinatal outcome	13 / 282 (4.7)	6 / 210 (2.9)	15 / 268 (5.6)	0.60 (0.23–1.62)	1.21 (0.56–2.60)	0.354[‡]
NICU admission or death	6 / 282 (2.1)	4 / 210 (1.9)	8 / 268 (3.0)	0.89 (0.25–3.21)	1.42 (0.48–4.13)	0.726[†]
NICU admission	3 / 282 (1.1)	3 / 210 (1.4)	4 / 268 (1.5)	1.35 (0.27–6.75)	1.41 (0.31–6.36)	0.925[†]
Noninfectious renal and urological conditions (any recorded condition)	2 / 282 (0.7)	3 / 210 (1.4)	1 / 268 (0.4)	2.03 (0.34–12.25)	0.52 (0.05–5.82)	0.515[†]
Other cardiovascular conditions (any recorded condition)	3 / 282 (1.1)	1 / 210 (0.5)	2 / 268 (0.7)	0.44 (0.05–4.31)	0.70 (0.12–4.22)	0.883[†]
Hematological conditions and thrombophilias (any recorded condition)	4 / 282 (1.4)	3 / 210 (1.4)	1 / 268 (0.4)	1.01 (0.22–4.55)	0.26 (0.03–2.34)	0.486[†]
Autoimmune conditions (any recorded condition)	0 / 282 (0.0)	2 / 210 (1.0)	1 / 268 (0.4)	6.77 (0.32–141.86)[¶]	3.17 (0.13–78.11)[¶]	0.194[†]
Gastrointestinal and hepatobiliary conditions and hernias (any recorded condition)	2 / 282 (0.7)	1 / 210 (0.5)	4 / 268 (1.5)	0.67 (0.06–7.44)	2.12 (0.39–11.68)	0.513[†]
Neurological conditions and other mental disorders (any recorded condition)	1 / 282 (0.4)	3 / 210 (1.4)	3 / 268 (1.1)	4.07 (0.42–39.43)	3.18 (0.33–30.77)	0.397[†]
Gynecological conditions, neoplasms, and masses of undetermined nature (any recorded condition)	3 / 282 (1.1)	1 / 210 (0.5)	3 / 268 (1.1)	0.44 (0.05–4.31)	1.05 (0.21–5.26)	0.797[†]
Other maternal infections (any recorded condition)	2 / 282 (0.7)	5 / 210 (2.4)	4 / 268 (1.5)	3.41 (0.66–17.77)	2.12 (0.39–11.68)	0.269[†]
Other specified clinical conditions and medical history (any recorded condition)	3 / 282 (1.1)	2 / 210 (1.0)	3 / 268 (1.1)	0.89 (0.15–5.40)	1.05 (0.21–5.26)	1.000[†]

Marker or comparison	Normal weight *n* / *N* (%)	Overweight *n* / *N* (%)	Obesity *n* / *N* (%)	Overweight OR (95% CI)	Obesity OR (95% CI)	Global *p*-Value
Other fetal heart rate abnormalities (any recorded condition)	1 / 282 (0.4)	1 / 210 (0.5)	0 / 268 (0.0)	1.34 (0.08–21.62)	0.35 (0.01–8.62)[¶]	0.733[†]
Fetal congenital anomalies (any recorded condition)	1 / 282 (0.4)	2 / 210 (1.0)	2 / 268 (0.7)	2.70 (0.24–30.00)	2.11 (0.19–23.44)	0.742[†]
Placental abnormalities (any recorded condition)	2 / 282 (0.7)	1 / 210 (0.5)	2 / 268 (0.7)	0.67 (0.06–7.44)	1.05 (0.15–7.53)	1.000[†]
Other obstetric, delivery, and puerperal conditions (any recorded condition)	1 / 282 (0.4)	4 / 210 (1.9)	1 / 268 (0.4)	5.46 (0.61–49.18)	1.05 (0.07–16.91)	0.101[†]
Cesarean delivery	170 / 282 (60.4)	170 / 210 (81.0)	221 / 268 (82.5)	2.78 (1.83–4.23)	3.08 (2.07–4.57)	<0.001[‡]
Preterm birth (<37 weeks)	46 / 282 (16.3)	35 / 210 (16.7)	45 / 268 (16.9)	1.03 (0.63–1.66)	1.04 (0.66–1.63)	0.989[‡]
Low birth weight (<2500 g)	40 / 282 (14.4)	34 / 210 (16.2)	28 / 268 (10.6)	1.15 (0.70–1.89)	0.70 (0.42–1.18)	0.182[‡]
Birth weight ≥4000 g	4 / 282 (1.5)	9 / 210 (4.3)	26 / 268 (9.7)	3.04 (0.92–10.01)	7.30 (2.51–21.20)	<0.001[‡]
Small for gestational age	23 / 282 (8.1)	11 / 210 (5.2)	18 / 268 (6.6)	0.63 (0.30–1.32)	0.80 (0.42–1.53)	0.451[‡]
Large for gestational age	8 / 282 (2.9)	21 / 210 (10.0)	41 / 268 (15.3)	3.75 (1.63–8.64)	6.10 (2.80–13.27)	<0.001[‡]
5-minute Apgar <7 (applicable scores)	8 / 279 (2.9)	3 / 209 (1.5)	8 / 264 (3.0)	0.49 (0.13–1.86)	1.04 (0.38–2.81)	0.492[‡]
Fetal death or stillbirth	3 / 282 (1.1)	1 / 210 (0.5)	4 / 268 (1.5)	0.44 (0.05–4.31)	1.41 (0.31–6.36)	0.544[†]
Episiotomy (vaginal deliveries)	44 / 112 (39.6)	21 / 40 (52.5)	16 / 47 (33.2)	1.68 (0.81–3.48)	0.76 (0.36–1.58)	0.185[‡]
Any perineal tear (vaginal deliveries)	27 / 112 (24.4)	7 / 40 (18.1)	19 / 47 (40.8)	0.69 (0.27–1.73)	2.13 (1.01–4.49)	0.049[‡]
Any previous pregnancy loss	3 / 282 (1.1)	2 / 210 (1.0)	8 / 268 (3.0)	0.89 (0.15–5.40)	2.86 (0.75–10.90)	0.145[†]
Maternal age ≥35 years	48 / 282 (17.0)	48 / 210 (22.9)	63 / 268 (23.5)	1.44 (0.92–2.26)	1.50 (0.98–2.28)	0.125[#]
Parity ≥2 deliveries	124 / 282 (44.0)	132 / 210 (62.8)	166 / 268 (61.8)	2.15 (1.49–3.10)	2.06 (1.46–2.90)	<0.001[‡]
Recorded surgery/labor duration >120 min (cesarean)	2 / 170 (1.2)	3 / 170 (1.8)	13 / 221 (5.9)	1.47 (0.24–8.93)	5.15 (1.15–23.15)	0.015[‡]
Recorded surgery/labor duration >120 min (vaginal)	1 / 112 (1.0)	0 / 40 (0.0)	2 / 47 (4.4)	0.88 (0.04–21.96)[¶]	4.77 (0.43–53.44)	0.203[‡]

BMI, body mass index; CI, confidence interval; NICU, neonatal intensive care unit; OR, odds ratio. *n*, category frequency or number of events, as specified; *N*, group size or analysis denominator. ORs are crude estimates, without adjustment for maternal age, parity, or other covariates. Binary counts are rounded means; nonevents equal the rounded denominator minus events. Subgroup denominators can vary across imputations. Displayed event counts and applicable denominators are rounded summaries across imputations. Percentages are based on unrounded completed-dataset proportions and may therefore differ from percentages calculated directly from the displayed integers. [#], Pearson chi-square without continuity correction. [†], Conditional Monte Carlo with fixed margins and 9999 replicates. [‡], D2 pooling of Pearson statistics; sparse-cell results are approximate. Unchanged markers retain observed-data tests. [¶], A continuity correction of 0.5 was added to all four cells of a 2 × 2 table containing a zero cell before pooling log odds ratios. These estimates are exploratory and may be highly imprecise. No multiplicity adjustment was applied. Recorded fetal macrosomia is distinct from birth weight ≥4000 g.

Table S11. Expanded multivariable sensitivity analyses and full coefficients of the core and expanded models.
Normal weight is the reference category. Core models include body mass index (BMI) category, age ≥35 versus <35 years, and parity ≥2 versus 1 delivery (four nonintercept coefficients). Estimates and covariance matrices were pooled using Rubin's rules with Barnard–Rubin degrees of freedom. Global BMI *p*-Values use the pooled D1 multivariate Wald test with a finite-sample correction. Adjustment variables were selected on clinical grounds, without bivariate *p*-Value screening. Expanded models additionally include smoking, chronic hypertension, and pre-existing diabetes mellitus (seven nonintercept coefficients). They were attempted only with ≥70 events and ≥70 nonevents overall. Expanded models that had separation, a rank-deficient design, or unstable estimates were not pooled.

Outcome and model	Term	aOR (95% CI)	*p*-Value	Global BMI *p*-Value
**Maternal clinical conditions**	***n* = 760**			**<0.001**
**Core model**	Overweight vs normal weight	1.62 (1.06–2.49)	0.026	
	Obesity vs normal weight	3.04 (2.06–4.49)	<0.001	
	Age ≥35 vs <35 years	1.77 (1.20–2.62)	0.004	
	Parity ≥2 vs 1 delivery	0.81 (0.58–1.14)	0.221	
**Chronic hypertension**	***n* = 760**			**<0.001**
**Core model**	Overweight vs normal weight	1.54 (0.59–4.03)	0.374	
	Obesity vs normal weight	8.96 (4.14–19.38)	<0.001	
	Age ≥35 vs <35 years	3.03 (1.75–5.25)	<0.001	
	Parity ≥2 vs 1 delivery	1.18 (0.67–2.06)	0.564	
**Hypothyroidism**	***n* = 760**			**0.047**
**Core model**	Overweight vs normal weight	1.47 (0.62–3.50)	0.381	
	Obesity vs normal weight	2.50 (1.18–5.28)	0.017	
	Age ≥35 vs <35 years	0.85 (0.38–1.92)	0.695	
	Parity ≥2 vs 1 delivery	0.70 (0.37–1.32)	0.266	
**Urinary tract infection**	***n* = 760**			**0.399**
**Core model**	Overweight vs normal weight	1.77 (0.72–4.34)	0.209	
	Obesity vs normal weight	1.14 (0.45–2.87)	0.787	
	Age ≥35 vs <35 years	1.02 (0.42–2.49)	0.958	
	Parity ≥2 vs 1 delivery	1.20 (0.56–2.60)	0.638	
**Asthma**	***n* = 760**			**0.483**
**Core model**	Overweight vs normal weight	1.64 (0.61–4.37)	0.325	
	Obesity vs normal weight	1.71 (0.68–4.29)	0.257	
	Age ≥35 vs <35 years	1.34 (0.54–3.32)	0.530	
	Parity ≥2 vs 1 delivery	0.64 (0.29–1.40)	0.262	
**Obstetric complications**	***n* = 760**			**<0.001**
**Core model**	Overweight vs normal weight	2.12 (1.42–3.16)	<0.001	
	Obesity vs normal weight	4.12 (2.83–5.99)	<0.001	
	Age ≥35 vs <35 years	1.75 (1.20–2.56)	0.004	
	Parity ≥2 vs 1 delivery	0.91 (0.66–1.26)	0.577	
**Obstetric complications**	***n* = 760**			**<0.001**
**Expanded model**	Overweight vs normal weight	2.09 (1.40–3.13)	<0.001	
	Obesity vs normal weight	3.90 (2.65–5.73)	<0.001	
	Age ≥35 vs <35 years	1.70 (1.16–2.50)	0.007	
	Parity ≥2 vs 1 delivery	0.92 (0.66–1.27)	0.597	
	Smoking	0.83 (0.44–1.58)	0.577	
	Chronic hypertension	1.30 (0.78–2.19)	0.313	
	Pre-existing diabetes mellitus	1.29 (0.43–3.93)	0.648	
**Gestational diabetes mellitus**	***n* = 760**			**0.002**
**Core model**	Overweight vs normal weight	1.84 (1.08–3.14)	0.025	
	Obesity vs normal weight	2.43 (1.48–3.97)	<0.001	
	Age ≥35 vs <35 years	1.63 (1.04–2.57)	0.034	
	Parity ≥2 vs 1 delivery	1.15 (0.76–1.76)	0.504	
**Hypertensive disorders of pregnancy**	***n* = 760**			**<0.001**
**Core model**	Overweight vs normal weight	1.81 (0.97–3.37)	0.063	
	Obesity vs normal weight	6.12 (3.60–10.40)	<0.001	
	Age ≥35 vs <35 years	1.43 (0.89–2.31)	0.139	
	Parity ≥2 vs 1 delivery	0.79 (0.52–1.21)	0.281	
	***n* = 760**			**<0.001**

Outcome and model	Term	aOR (95% CI)	*p*-Value	Global BMI *p*-Value
**Hypertensive disorders of pregnancy**	Overweight vs normal weight	1.83 (0.98–3.43)	0.057	
**Expanded model**	Obesity vs normal weight	6.82 (3.96–11.73)	<0.001	
	Age ≥35 vs <35 years	1.54 (0.95–2.49)	0.083	
	Parity ≥2 vs 1 delivery	0.79 (0.52–1.22)	0.292	
	Smoking	1.21 (0.54–2.68)	0.645	
	Chronic hypertension	0.57 (0.30–1.09)	0.091	
	Pre-existing diabetes mellitus	0.58 (0.12–2.82)	0.495	
**Composite adverse perinatal outcome**	***n* = 760**			**0.368**
**Core model**	Overweight vs normal weight	0.58 (0.21–1.57)	0.284	
	Obesity vs normal weight	1.16 (0.54–2.53)	0.703	
	Age ≥35 vs <35 years	1.64 (0.74–3.64)	0.225	
	Parity ≥2 vs 1 delivery	1.03 (0.49–2.17)	0.941	
**Cesarean delivery**	***n* = 760**			**<0.001**
**Core model**	Overweight vs normal weight	2.87 (1.87–4.39)	<0.001	
	Obesity vs normal weight	3.18 (2.12–4.75)	<0.001	
	Age ≥35 vs <35 years	0.77 (0.51–1.17)	0.223	
	Parity ≥2 vs 1 delivery	0.94 (0.66–1.34)	0.744	
**Preterm birth (<37 weeks)**	***n* = 760**			**0.981**
**Core model**	Overweight vs normal weight	0.95 (0.58–1.56)	0.852	
	Obesity vs normal weight	0.97 (0.61–1.53)	0.893	
	Age ≥35 vs <35 years	1.26 (0.79–2.00)	0.331	
	Parity ≥2 vs 1 delivery	1.37 (0.90–2.08)	0.138	
**Preterm birth (<37 weeks)**	***n* = 760**			**0.766**
**Expanded model**	Overweight vs normal weight	0.95 (0.58–1.57)	0.849	
	Obesity vs normal weight	0.84 (0.51–1.37)	0.476	
	Age ≥35 vs <35 years	1.16 (0.72–1.88)	0.543	
	Parity ≥2 vs 1 delivery	1.22 (0.79–1.87)	0.366	
	Smoking	2.25 (1.19–4.25)	0.013	
	Chronic hypertension	1.92 (1.05–3.54)	0.036	
	Pre-existing diabetes mellitus	4.56 (1.52–13.74)	0.007	
**Low birth weight (<2500 g)**	***n* = 760**			**0.143**
**Core model**	Overweight vs normal weight	1.05 (0.63–1.74)	0.845	
	Obesity vs normal weight	0.64 (0.38–1.08)	0.097	
	Age ≥35 vs <35 years	1.48 (0.90–2.41)	0.120	
	Parity ≥2 vs 1 delivery	1.46 (0.92–2.31)	0.109	
**Low birth weight (<2500 g)**	***n* = 760**			**0.024**
**Expanded model**	Overweight vs normal weight	1.06 (0.63–1.77)	0.838	
	Obesity vs normal weight	0.50 (0.28–0.89)	0.018	
	Age ≥35 vs <35 years	1.33 (0.80–2.21)	0.279	
	Parity ≥2 vs 1 delivery	1.26 (0.79–2.03)	0.332	
	Smoking	2.67 (1.38–5.17)	0.004	
	Chronic hypertension	2.58 (1.32–5.06)	0.006	
	Pre-existing diabetes mellitus	4.64 (1.48–14.54)	0.008	
**Small for gestational age**	***n* = 760**			**0.478**
**Core model**	Overweight vs normal weight	0.63 (0.30–1.34)	0.227	
	Obesity vs normal weight	0.81 (0.42–1.57)	0.529	
	Age ≥35 vs <35 years	0.58 (0.25–1.35)	0.208	
	Parity ≥2 vs 1 delivery	1.12 (0.61–2.03)	0.716	
**Large for gestational age**	***n* = 760**			**<0.001**
**Core model**	Overweight vs normal weight	3.68 (1.59–8.54)	0.002	
	Obesity vs normal weight	5.99 (2.74–13.13)	<0.001	
	Age ≥35 vs <35 years	0.90 (0.48–1.69)	0.747	
	Parity ≥2 vs 1 delivery	1.15 (0.67–1.96)	0.618	
**Large for gestational age**	***n* = 760**			**<0.001**
**Expanded model**	Overweight vs normal weight	3.58 (1.54–8.33)	0.003	
	Obesity vs normal weight	5.58 (2.51–12.39)	<0.001	
	Age ≥35 vs <35 years	0.88 (0.47–1.67)	0.699	

Outcome and model	Term	aOR (95% CI)	*p*-Value	Global BMI *p*-Value
	Parity ≥2 vs 1 delivery	1.21 (0.71–2.08)	0.485	
	Smoking	0.38 (0.09–1.64)	0.194	
	Chronic hypertension	1.35 (0.66–2.76)	0.416	
	Pre-existing diabetes mellitus	0.63 (0.08–5.06)	0.663	
**Episiotomy (vaginal deliveries)**	***n* = 198–199**			**0.122**
**Core model**	Overweight vs normal weight	2.44 (1.06–5.65)	0.037	
	Obesity vs normal weight	1.33 (0.56–3.15)	0.512	
	Age ≥35 vs <35 years	1.20 (0.51–2.82)	0.674	
	Parity ≥2 vs 1 delivery	0.15 (0.07–0.31)	<0.001	
**Any perineal tear (vaginal deliveries)**	***n* = 198–199**			**0.132**
**Core model**	Overweight vs normal weight	0.62 (0.24–1.61)	0.320	
	Obesity vs normal weight	1.73 (0.78–3.83)	0.176	
	Age ≥35 vs <35 years	0.80 (0.35–1.83)	0.588	
	Parity ≥2 vs 1 delivery	2.67 (1.23–5.78)	0.013	

aOR, adjusted odds ratio; BMI, body mass index; CI, confidence interval; n, category frequency or number of events, as specified. Intercepts are omitted. *p*-Values for individual coefficients were obtained from pooled t-tests using Barnard–Rubin degrees of freedom. Global BMI *p*-Values were obtained from the pooled D1 multivariate Wald test with a finite-sample correction. All reported odds ratios are adjusted estimates from the specified core or expanded model. No model included gestational complications, preterm birth, or delivery route as routine adjustment covariates because these may be downstream of BMI. Gravidity and parity were not entered together. The observed overall clinical-condition marker was not treated as a verified baseline confounder. Expanded gestational diabetes and cesarean models were unstable; the expanded episiotomy model had a rank-deficient design. Their core models remained estimable and are reported.

Table S12. Complete-case sensitivity analyses of the core age- and parity-adjusted body mass index (BMI) associations. Each complete-case model excludes women with missing outcome, age, or parity for that specific comparison. BMI was observed in all included participants, and underweight was excluded. For individual coefficients, two-sided *p*-Values and 95% confidence intervals were calculated using a t distribution with adjusted complete-data degrees of freedom, $\nu = d(d + 1)/(d + 3)$, where $d = n - p$ and p is the number of estimated regression parameters, including the intercept. Confidence intervals were calculated on the log-odds scale and exponentiated. Global BMI *p*-Values were obtained from a conventional Wald chi-square test with two degrees of freedom. The crude and adjusted multiple-imputation estimates in the main article remain the primary results.

**Outcome**	**Complete cases *n* / events**	**BMI contrast**	**aOR (95% CI)**	***p*-Value contrast**	**Global BMI *p*-Value**
Maternal clinical conditions	745 / 225	Overweight	1.60 (1.04–2.46)	0.032	<0.001
		Obesity	3.04 (2.05–4.50)	<0.001	
Chronic hypertension	745 / 75	Overweight	1.54 (0.59–4.01)	0.379	<0.001
		Obesity	8.93 (4.12–19.35)	<0.001	
Hypothyroidism	745 / 44	Overweight	1.44 (0.60–3.42)	0.412	0.064
		Obesity	2.39 (1.12–5.10)	0.024	
Urinary tract infection	745 / 31	Overweight	1.75 (0.71–4.28)	0.222	0.425
		Obesity	1.15 (0.45–2.90)	0.772	
Asthma	745 / 29	Overweight	1.61 (0.60–4.31)	0.339	0.474
		Obesity	1.74 (0.69–4.38)	0.243	
Obstetric complications	745 / 288	Overweight	2.04 (1.37–3.05)	<0.001	<0.001
		Obesity	4.11 (2.81–6.00)	<0.001	
Gestational diabetes mellitus	745 / 124	Overweight	1.81 (1.06–3.10)	0.029	0.002
		Obesity	2.47 (1.51–4.05)	<0.001	
Hypertensive disorders of pregnancy	745 / 125	Overweight	1.70 (0.90–3.19)	0.099	<0.001
		Obesity	5.95 (3.49–10.14)	<0.001	
Composite adverse perinatal outcome	740 / 32	Overweight	0.58 (0.22–1.58)	0.287	0.483
		Obesity	1.03 (0.46–2.30)	0.940	
Cesarean delivery	743 / 553	Overweight	2.90 (1.88–4.46)	<0.001	<0.001
		Obesity	3.38 (2.23–5.12)	<0.001	
Preterm birth (<37 weeks)	739 / 122	Overweight	0.96 (0.59–1.57)	0.868	0.973
		Obesity	0.95 (0.59–1.51)	0.821	
Low birth weight (<2500 g)	737 / 99	Overweight	1.07 (0.64–1.79)	0.792	0.130
		Obesity	0.64 (0.37–1.09)	0.098	
Small for gestational age	742 / 49	Overweight	0.66 (0.31–1.42)	0.288	0.566
		Obesity	0.84 (0.43–1.65)	0.614	
Large for gestational age	742 / 68	Overweight	3.64 (1.57–8.44)	0.003	<0.001
		Obesity	5.78 (2.62–12.71)	<0.001	
Episiotomy (vaginal deliveries)	188 / 75	Overweight	2.52 (1.07–5.90)	0.034	0.079
		Obesity	1.01 (0.41–2.49)	0.985	
Any perineal tear (vaginal deliveries)	185 / 51	Overweight	0.64 (0.24–1.67)	0.357	0.071
		Obesity	2.04 (0.90–4.62)	0.085	

aOR, adjusted odds ratio; BMI, body mass index; CI, confidence interval; n, category frequency or number of events, as specified. Normal weight is the reference. Models adjust for age ≥35 versus <35 years and parity ≥2 versus 1 delivery. Global BMI *p*-Values use a conventional two-degree-of-freedom Wald chi-square test. Complete-case denominators are marker specific and should not be interpreted as a different eligibility cohort.

Table S13. Analytical feasibility and departures from the expanded analysis specification.

Analysis	Disposition	Reason or reporting consequence
Pre-existing diabetes mellitus (core model)	Not fitted	Insufficient event information for adjusted model. Reported descriptively and through exploratory bivariate comparisons.
Syphilis (core model)	Not fitted	Insufficient event information for adjusted model. Reported descriptively and through exploratory bivariate comparisons.
Depression (core model)	Not fitted	Insufficient event information for adjusted model. Reported descriptively and through exploratory bivariate comparisons.
Gestational diabetes mellitus (expanded model)	Not estimable	Separation or unstable/nonconvergent estimate. Retained the estimable core model; no unstable expanded estimate was pooled.
Fetal growth abnormalities (core model)	Not fitted	Insufficient event information for adjusted model. Reported descriptively and through exploratory bivariate comparisons.
Intrauterine growth restriction (core model)	Not fitted	Insufficient event information for adjusted model. Reported descriptively and through exploratory bivariate comparisons.
Fetal macrosomia (core model)	Not fitted	Insufficient event information for adjusted model. Reported descriptively and through exploratory bivariate comparisons.
Fetal status abnormalities (core model)	Not fitted	Insufficient event information for adjusted model. Reported descriptively and through exploratory bivariate comparisons.
Fetal distress (core model)	Not fitted	Insufficient event information for adjusted model. Reported descriptively and through exploratory bivariate comparisons.
Amniotic fluid abnormalities (core model)	Not fitted	Insufficient event information for adjusted model. Reported descriptively and through exploratory bivariate comparisons.
Polyhydramnios (core model)	Not fitted	Insufficient event information for adjusted model. Reported descriptively and through exploratory bivariate comparisons.
Oligohydramnios (core model)	Not fitted	Insufficient event information for adjusted model. Reported descriptively and through exploratory bivariate comparisons.
Rupture of membranes (core model)	Not fitted	Insufficient event information for adjusted model. Reported descriptively and through exploratory bivariate comparisons.
NICU admission or death (core model)	Not fitted	Insufficient event information for adjusted model. Reported descriptively and through exploratory bivariate comparisons.
NICU admission (core model)	Not fitted	Insufficient event information for adjusted model. Reported descriptively and through exploratory bivariate comparisons.
Cesarean delivery (expanded model)	Not estimable	Separation or unstable/nonconvergent estimate. Retained the estimable core model; no unstable expanded estimate was pooled.
Birth weight ≥4000 g (core model)	Not fitted	Insufficient event information for adjusted model. Reported descriptively and through exploratory bivariate comparisons.
5-minute Apgar <7 (applicable scores) (core model)	Not fitted	Insufficient event information for adjusted model. Reported descriptively and through exploratory bivariate comparisons.
Fetal death or stillbirth (core model)	Not fitted	Insufficient event information for adjusted model. Reported descriptively and through exploratory bivariate comparisons.
Episiotomy (vaginal deliveries) (expanded model)	Not estimable	Rank-deficient design. Retained the estimable core model; no unstable expanded estimate was pooled.
Recorded surgery/labor duration >120 min (cesarean) (core model)	Not fitted	Insufficient event information for adjusted model. Reported descriptively and through exploratory bivariate comparisons.
Recorded surgery/labor duration >120 min (vaginal) (core model)	Not fitted	Insufficient event information for adjusted model. Reported descriptively and through exploratory bivariate comparisons.
Continuous BMI, obesity classes, or gestational weight gain	Unavailable	Only BMI categories were supplied; these analyses were not performed.
Adjustment for previous cesarean delivery	Unavailable	A validated previous-cesarean field was unavailable. Generic previous surgery and parity were not substituted for it.
Restriction to verified pre-pregnancy / ≤13-week BMI	Unavailable	Record-level timing and measured versus reported anthropometry were not supplied.
Numerical Apgar global MI comparison	Not pooled	Extremely rare numerical-score category support varied across imputations. The grouped Apgar comparison was used.
Penalized regression, BMI interactions, or outcome-specific power calculation	Not performed	These procedures were not included in the executed analysis. Sparse components were not subjected to forced multivariable modeling.

BMI, body mass index; MI, multiple imputation; NICU, neonatal intensive care unit. Core models required ≥20 events overall and ≥5 events and nonevents within each BMI group, with a stated exception for the primary composite. Expanded models used the same BMI/age/parity specification plus smoking, chronic hypertension, and pre-existing diabetes, and were attempted only

when there were at least 70 events and 70 nonevents. No *p*-Value was averaged across imputations and no stacked-imputation file was treated as a sample 50 times larger. Confidence intervals can remain wide even in a successfully fitted model.

D1 denotes the multivariate Wald pooling procedure, which combines regression coefficient estimates and their covariance matrices across imputations; it was used for joint tests of the BMI coefficients. D2 denotes the procedure used to combine Pearson chi-square statistics across imputations for global categorical comparisons (Enders formulation; approximate F distribution). Neither procedure averages *p*-Values.

Pooling references: Li, Raghunathan, and Rubin (1991), multivariate D1 Wald pooling; Li, Meng, Raghunathan, and Rubin (1991), D2 pooling; Rubin (1987), coefficient pooling; Barnard and Rubin (1999), scalar degrees of freedom; Reiter (2007), finite-sample D1 correction. Formula implementation was checked against the documented mitml source (https://rdrr.io/cran/mitml/src/R/internal-pool.R) and Flexible Imputation of Missing Data (https://stefvanbuuren.name/fimd/sec-multiparameter.html).
